# Supplementary material for: What treatments work for anxiety and depression in children and adolescents with chronic fatigue syndrome? An updated systematic review
Source: BMJ Open. 2022 Jan 31;12(1):e051358. doi: 10.1136/bmjopen-2021-051358 (PMC8808375; doi:10.1136/bmjopen-2021-051358)
Supplement: Supplementary data [file bmjopen-2021-051358supp001.pdf]

Supplementary Material

Appendix 1: Search Strategies

Search strategy for Anxiety searches:

1. (adolesc* or preadolesc* or pre-adolesc* or boy* or girl* or child* or infan* or preschool* or pre-school* or juvenil* or minor* or pe?diatri* or pubescen* or pre-pubescen* or prepubescen* or puberty or teen* or young* or youth* or school* or high-school* or highschool* or sibling* or schoolchild* or school child* or children).tw.
2. exp Adolescent/ or exp Child/ or exp Child, Preschool/ or exp Infant/ or exp Minors/ or exp Pediatrics/
3. 1 or 2
4. Chronic Fatigue Syndrome.tw
5. myalgic encephal*.tw.
6. chronic fatigue syndrome*.mp.
7. myalgic encephal*.mp.
8. anxiety disorder/
9. exp anxiety disorder
10. exp obsessive-compulsive disorder
11. exp panic
12. anxi*.tw
13. generali#ed anxiety disorder.tw
14. obsessive compulsive.tw
15. OCD.tw
16. Phobia*.tw
17. Social anxiety.tw
18. Separation anxiety.tw

19. Panic.tw
20. exp Chronic Fatigue Syndrome/
21. exp Anxiety Disorders/ or exp Social Phobia/ or exp Panic Disorder/ or exp Anxiety/ or exp Social Anxiety
22. exp Separation Anxiety Disorder/ or Separation Anxiety/
23. exp Generalized Anxiety Disorder
24. exp Obsessive Compulsive Disorder
25. exp Phobias/
26. 4 or 5 or 6 or 7 or 20
27. 8 or 9 or 10 or 11 or 12 or 13 or 14 or 15 or 16 or 17 or 18 or 19 or 21 or 22 or 23 or 24 or 25
28. 3 and 26 and 27
29. Limit 28 to yr="2016-current"

Search strategy for Depression searches:

1. (adolesc* or preadolesc* or pre-adolesc* or boy* or girl* or child* or infan* or preschool* or pre-school* or juvenil* or minor* or pe?diatri* or pubescen* or pre-pubescen* or prepubescen* or puberty or teen* or young* or youth* or school* or high-school* or highschool* or sibling* or schoolchild* or school child* or children).tw.
2. exp Adolescent/ or exp Child/ or exp Child, Preschool/ or exp Infant/ or exp Minors/ or exp Pediatrics/
3. 1 or 2
4. chronic fatigue syndrome*.mp.
5. exp Chronic Fatigue Syndrome
6. Chronic Fatigue Syndrome.tw
7. myalgic encephal*.mp.

8. myalgic encephal*.tw.
9. 4 or 5 or 6 or 7 or 8
10. depressive disorder.mp.
11. exp depression/
12. depress*.tw
13. dysthymi*.tw
14. exp adjustment disorders/
15. adjustment disorder* .mp.
16. low mood.tw.
17. 10 or 11 or 12 or 14 or 14 or 15 or 16
18. 3 and 9 and 17
19. Limit 18 to yr = “2015 – current

Clery P, *et al. BMJ Open* 2022; 12:e051358. doi: 10.1136/bmjopen-2021-051358

Appendix 2: Quality Assessment

Supplementary Table 1: Quality Assessment of all studies included in this updated review, using Cochrane Risk of Bias scales ROBINS-I and RoB-2

(a) Observational Studies

Authors (year)	Did the study address a clearly focused issue? Was this the outcome of interest to this review?	Was the cohort recruited in an acceptable way?	Was the exposure accurately measured to minimise bias?	Was the outcome accurately measured to minimise bias?	Confounding factors?	Follow-up of subjects complete enough and long enough?	Overall Rating using Cochrane risk of bias scale ROBINS-I (low/unclear/high)
**Chalder et al (2002)**	Yes, No.	Yes	Yes	Yes	Can't tell	Can't tell, yes	Unclear
**Diaz-Caneja et al (2007)**	Can't tell, No	Can't tell	Can't tell	Can't tell	Yes	Yes, no	High
**Lloyd et al (2012); Rimes et al (2014)**	Yes, No	Yes	Yes	Yes	Can't tell	Can't tell, yes	Unclear
**Rimes et al (2007)**	Yes, No	Yes	Yes	Yes	Can't tell	Can't tell, yes	Unclear
**Van de Putte et al (2007)**	Yes, No	Yes	Yes	Yes	Can't tell	Can't tell, yes	Unclear
**Kawatani et al (2011)**	Yes, No	Yes (Case control)	Yes	Yes	Can't tell	No, Yes	High
**Gordon and Lubitz (2009)**	Yes, No	Yes (Case series)	No	No	Can't tell	Yes, No	High*
**Henderson (2014)**	Yes, No	No (Case series)	No	No	Can't tell	No, Yes	High*
**Denborough et al (2003)**	Yes, No	Yes (Case series)	No	Yes	Can't tell	Yes, Yes	High*
**Rowe (2019)**	No, No	No	Yes	No	No	Yes, Yes	Unclear

(b) Randomised controlled trials

Authors (year)	Did the trial address a clearly	Was the assignment of	Were patients, healthcare	Were the groups	Aside from the experimental	Were all of the patients who	Overall Rating using Cochrane

	focused issue? Was this the outcome of interest to this review?	patients to treatments randomised?	professionals and research staff blinded?	similar at the start of the trial?	investigation, were the groups treated equally?	entered the trial properly accounted for at its conclusion?	risk of bias scale RoB 2 (low/unclear/high)
**Nijhof et al (2012); Nijhof et al (2013)**	Yes, no	Yes	No	Yes	Yes	Can't tell	Low
**Rowe (1997)**	Yes, no	Yes	Yes	Yes	Yes	Yes	Low
**Wright et al (2005)**	Yes. no	Yes	No	Yes	Yes	Can't tell	Low
**Gordon et al (2010)**	Yes, no	Yes	No (pts), No (HCPs), Yes (assessors)	Yes	Yes	Yes	Low
**Crawley et al (2018)**	Yes, no	Yes	No	Yes	Yes	Yes	Low
