# Supplementary material for: What treatments work for anxiety and depression in children and adolescents with chronic fatigue syndrome? An updated systematic review
Source: BMJ Open. 2022 Jan 31;12(1):e051358. doi: 10.1136/bmjopen-2021-051358 (PMC8808375; doi:10.1136/bmjopen-2021-051358)
Supplement: Author's manuscript [file bmjopen-2021-051358.draft_revisions.pdf]

BMJ Open is committed to open peer review. As part of this commitment we make the peer review history of every article we publish publicly available.

When an article is published we post the peer reviewers' comments and the authors' responses online. We also post the versions of the paper that were used during peer review. These are the versions that the peer review comments apply to.

The versions of the paper that follow are the versions that were submitted during the peer review process. They are not the versions of record or the final published versions. They should not be cited or distributed as the published version of this manuscript.

BMJ Open is an open access journal and the full, final, typeset and author-corrected version of record of the manuscript is available on our site with no access controls, subscription charges or pay-per-view fees (http://bmjopen.bmj.com).

If you have any questions on BMJ Open's open peer review process please email info.bmjopen@bmj.com

BMJ Open

What treatments work for anxiety and depression in children and adolescents with Chronic Fatigue Syndrome? An updated systematic review

Journal:	*BMJ Open*
Manuscript ID	bmjopen-2021-051358
Article Type:	Original research
Date Submitted by the Author:	18-Mar-2021
Complete List of Authors:	Clery, Philippa; University of Bristol, Centre for Academic Child Health<br>Royston, Alexander; University of Bristol, Centre for Academic Child Health<br>Driver, Katie; University of Bristol, Centre for Academic Child Health<br>Bailey, Jasmine; University of Bristol, Centre for Academic Child Health<br>Crawley, Esther; University of Bristol, Centre for Academic Child Health; Royal United Hospitals Bath NHS Foundation Trust, Paediatric Chronic Fatigue Syndrome Specialist Service<br>Loades, Maria; University of Bristol, Centre for Academic Child Health; University of Bath, Department of Psychology
Keywords:	PAEDIATRICS, Anxiety disorders < PSYCHIATRY, Depression & mood disorders < PSYCHIATRY, MENTAL HEALTH, Child & adolescent psychiatry < PSYCHIATRY

SCHOLARONE™
Manuscripts

I, the Submitting Author has the right to grant and does grant on behalf of all authors of the Work (as defined in the below author licence), an exclusive licence and/or a non-exclusive licence for contributions from authors who are: i) UK Crown employees; ii) where BMJ has agreed a CC-BY licence shall apply, and/or iii) in accordance with the terms applicable for US Federal Government officers or employees acting as part of their official duties; on a worldwide, perpetual, irrevocable, royalty-free basis to BMJ Publishing Group Ltd ("BMJ") its licensees and where the relevant Journal is co-owned by BMJ to the co-owners of the Journal, to publish the Work in this journal and any other BMJ products and to exploit all rights, as set out in our licence.

The Submitting Author accepts and understands that any supply made under these terms is made by BMJ to the Submitting Author unless you are acting as an employee on behalf of your employer or a postgraduate student of an affiliated institution which is paying any applicable article publishing charge ("APC") for Open Access articles. Where the Submitting Author wishes to make the Work available on an Open Access basis (and intends to pay the relevant APC), the terms of reuse of such Open Access shall be governed by a Creative Commons licence – details of these licences and which Creative Commons licence will apply to this Work are set out in our licence referred to above.

Other than as permitted in any relevant BMJ Author's Self Archiving Policies, I confirm this Work has not been accepted for publication elsewhere, is not being considered for publication elsewhere and does not duplicate material already published. I confirm all authors consent to publication of this Work and authorise the granting of this licence.

For peer review only

What treatments work for anxiety and depression in children and adolescents with Chronic Fatigue Syndrome? An updated systematic review

Philippa Clery[1], Alexander Royston[1], Katie Driver[1], Jasmine Bailey[1], Esther Crawley[1,2], Maria Loades[1,3]

[1] Centre for Academic Child Health, Bristol Medical School, Bristol, UK

[2] Paediatric Chronic Fatigue Syndrome Specialist Service, Royal United Hospitals Bath NHS Foundation Trust, Bath, UK

[3] Department of Psychology, University of Bath, Bath, UK

Corresponding author:

Dr Philippa Clery

Centre for Academic Child Health, 1-5 Whiteladies Road, Bristol, BS8 1NU, UK.

Philippa.clery@bristol.ac.uk

Word count: 2711

For peer review only

ABSTRACT

Objectives

Children with Chronic Fatigue Syndrome/Myalgic Encephalomyelitis (CFS/ME) experience a higher prevalence of depression and anxiety compared to age-matched controls. Our previous systematic reviews in 2015/16 found little evidence for effective treatment for children with CFS/ME with comorbid depression and/or anxiety. This review updates these findings.

Design

A systematic review. We searched Cochrane library, Medline, Embase and PsychINFO databases from 2015 to 2020.

Participants

Inclusion criteria: 1) < 18 years old; 2) diagnosed with CFS/ME (using Centre for Disease Control and Prevention, National Institute for Health and Care Excellence, or Oxford criteria); 3) validated measures of depression and/or anxiety.

Interventions

Observational studies or randomised controlled trials (RCT).

Comparison

Any or none.

Outcomes

Studies with outcome measures of anxiety, depression, or fatigue on validated assessments.

Results

Of 1040 papers identified, seven were paediatric CFS/ME intervention studies, of which two measured depression and/or anxiety outcomes. One study was an RCT, suggesting the Lightening

For peer review only - http://bmjopen.bmj.com/site/about/guidelines.xhtml

Process intervention in addition to specialist medical care (SMC) was more effective at reducing depressive and anxiety symptoms compared to SMC alone. The other was a retrospective observational cohort study evaluating routine specialist care. It measured anxiety and depression at baseline but not at follow-up. Neither study specifically targeted depression nor anxiety.

Conclusion

Very few paediatric CFS/ME intervention studies have been conducted in the last five years. Even fewer measured depression and/or anxiety outcomes (one of which was conducted by our own research team). There is continued lack of evidence identifying effective treatments for comorbid depression and/or anxiety in paediatric CFS/ME. We still do not know what treatment should be offered for these children.

Trial registration number

This review was an update of two previous reviews registered on the Prospective Register of Systematic Review Protocols (PROSPERO):

http://www.crd.york.ac.uk/PROSPERO/display_record.asp?ID=CRD42016043488 ;

http://www.crd.york.ac.uk/PROSPERO/display_record.asp?ID=CRD42015016813.

Key words

Paediatric, CFS/ME, chronic fatigue syndrome, anxiety, depression

ARTICLE SUMMARY

Strengths and limitations of study

- This updated review used a systematic approach to identify evidence for treatment approaches for comorbid anxiety and/or depression in paediatric CFS/ME.
- Non-English language articles were included.

For peer review only - http://bmjopen.bmj.com/site/about/guidelines.xhtml

- Authors were contacted and sub-group data obtained when available.
- Grey literature and unpublished material was not included.
- There was insufficient data to carry out a meta-analysis.

INTRODUCTION

Chronic fatigue syndrome (CFS)/myalgic encephalomyelitis (ME) is a common but poorly understood condition causing disabling fatigue, malaise, myalgia, sleep difficulties, and problems concentrating[1]. In children and adolescents (henceforth referred to as children), prevalence is estimated at 0.55% (95%CI 0.22-1.35) across community, primary care and hospital populations[2]. CFS/ME has long-term impacts on children's physical, cognitive, emotional and social functioning[3, 4].

Children with CFS/ME suffer from higher rates of both depression and anxiety than age-matched population samples. The prevalence estimates of comorbid depression and anxiety are 20%[5] and 29%[6], respectively, compared to 2.1% and 7.2%[7] in adolescents without CFS/ME. In those attending a specialist CFS/ME service, 61% who meet diagnostic criteria for depression also have an anxiety disorder[5]. Having comorbid depression and/or anxiety is associated with less favourable outcomes and may impact on engaging with treatment. Comorbid depression in paediatric CFS/ME is associated with greater functional disability, worse fatigue and more pain compared with those without depression[8, 9]. Low mood, anergia and anhedonia could be barriers to motivation to engage in behavioural treatment approaches and Cognitive Behavioural Therapy-for-fatigue (CBT-f). Depressive symptoms are therefore likely to require tailored treatment[9]. The impact of anxiety on outcomes is less clear. Given that most children with CFS/ME who have anxiety also have depression[5], it is important to explore treatments for both.

For peer review only - http://bmjopen.bmj.com/site/about/guidelines.xhtml

Despite the high prevalence of comorbid mental health problems, there is little evidence about the effectiveness of treatments. Our two previous systematic reviews looking at depression and anxiety outcomes in existing CFS/ME intervention studies found that no specifically adapted treatments had been trialled to target depression and anxiety in paediatric CFS/ME[10, 11]. Although CBT-f and a multicomponent inpatient programme showed promise in reducing depressive[10] and anxiety[11] symptoms, there was no consistent treatment approach for children with CFS/ME and comorbid depression or anxiety. Since conducting these reviews in 2015/16, further intervention studies may have been published. It is important and timely to review the current evidence to provide an update on what treatments should be offered to this population. Further, it is important to consider anxiety and depression together given their overlap, whereas previous reviews considered them separately.

We conducted an updated systematic review by synthesizing the evidence regarding treatments for paediatric CFS/ME and comorbid depression and anxiety since 2015. Specifically, we aimed to address the following:

1. What treatment approaches are there for depression and anxiety in children with CFS/ME?
2. What is known about the treatment efficacy of these approaches for treating depression and anxiety in CFS/ME? Do different approaches have different outcomes?

METHODS

Data sources and search strategy

We conducted searches on Medline, Embase, PsychINFO and Cochrane Library databases. We used the same search strategies from the previous systematic reviews (registered on Prospero: CRD42015016813; CRD42016043488) to repeat the depression and anxiety searches separately.

For peer review only - http://bmjopen.bmj.com/site/about/guidelines.xhtml

Searches were designed with input from an information specialist to include the concepts: paediatric; CFS/ME; anxiety and depression (search strategies are in supplementary material). We updated the searches from when they had last been run (February 2015 for depression search and July 2016 for anxiety search) up until September 2020. The two searches were carried out by different reviewer teams: anxiety search (PC, AR); depression search (KD, JB). Grey literature was not searched. Reference lists of articles for full-text screening were hand-searched.

Inclusion and exclusion Criteria

Studies were included if they met inclusion criteria (Table 1).

Table 1: Inclusion criteria

	Anxiety Review	Depression Review
**Participants**	1. Children <18 years of age<br>2. Diagnosed with CFS/ME defined using one of these criteria:<br>- CDC[12]<br>- NICE[1]<br>- Oxford[13]	
**Interventions**	Observational cohort studies<br>Any study with intervention – e.g., observational clinical cohorts, clinical trials, etc.	
**Baseline measure**	Validated assessment of anxiety	Validated assessment of depression
**Outcome measure**	Repeated measures of either anxiety and/or fatigue on psychometrically validated assessments or validated diagnostic interviews.	Repeated measures of either depression and/or fatigue on psychometrically validated assessments or validated diagnostic interviews.
**Language**	Non-English language papers were considered for inclusion.	

For peer review only - http://bmjopen.bmj.com/site/about/guidelines.xhtml

Study selection

Articles returned from database searches were inputted into Endnote and duplicates removed. Each reviewer conducted title and abstract screening independently. Full texts of potentially eligible articles were screened against specifically created eligibility checklists. The final articles for inclusion were cross-checked between all four reviewers and any conflicts discussed and resolved with input from the senior author (ML) if necessary. Where information from the paper was insufficient to determine eligibility, authors were contacted by email for additional information. If authors did not reply after two follow-up emails, the study was excluded. Figure 1 presents the PRISMA[12] flowchart.

Data extraction

For all included articles, data were extracted independently by two reviewers (PC, AR) using a purpose-designed data extraction form to collect information about: study design; setting; recruitment; participant characteristics; CFS/ME definition used for diagnosis; assessment of depression and anxiety; other outcomes; treatment and interventions provided; definition of response and treatment/intervention outcomes.

Quality assessment

PC and AR used Risk of Bias assessment tools[13, 14] to assess methodological quality of the included studies.

Data synthesis

For peer review only - http://bmjopen.bmj.com/site/about/guidelines.xhtml

We conducted a narrative synthesis[15] because there was insufficient comparable data to conduct a meta-analysis as interventions were heterogeneous and a range of outcome measures were reported. For each study, we compared the effects of interventions on outcomes, using mean differences. Different measures of anxiety and depression were used in each study, and one study did not have follow-up data, which limited direct comparison.

Patient and public involvement

No patients were involved.

Ethics approval

This study did not involve human participants.

RESULTS

Studies included

A total of 625 and 415 references were found by database searching for the depression and anxiety searches, respectively. After full-text screening, both searches returned the same two eligible studies[16, 17]. Study 1 was an RCT and study 2 was a retrospective observational cohort study. The PRISMA[12] flowchart is in Figure 1.

[Figure 1 here]

Quality assessment

For peer review only

For peer review only - http://bmjopen.bmj.com/site/about/guidelines.xhtml

The RCT (study 1)[17] was conducted by members of our CFS/ME research team (EC). The study has a low risk of bias from the concealed allocation randomisation process, minimal deviation from how interventions were intended to be delivered, and appropriate intention-to-treat analysis. Outcome measurement is biased because of self-reported measures, but this is standard for behavioural treatments. It is also biased due to loss to follow-up. In the control arm at 3 months, 13 of 49 (27%) were lost to follow-up and at the primary outcome of 6 months, 12 of 49 (24%) were not included in analysis. In the intervention arm 8 of 51 (16%) were lost to follow-up at 3 months and 7 of 51 (14%) were not included in primary analysis at 6 months. Although baseline characteristics between those who did and did not provide primary outcome data were similar, it is possible that missingness was related to the outcome. The retrospective observational study (study 2)[16] is also biased due to poor follow-up rates at any one time point (making comparison difficult), and no pre-published analysis plan. In the cohort, there are two samples; one with baseline data for anxiety and depression and one without. Follow-up questionnaires were mailed to all participants on a number of occasions between January 2008 and June 2011. This produced a range of follow-up time points (1-21 years) after illness onset, meaning some patients would not have had contact with the clinic for a long time when they were sent the questionnaire, so it is likely that both disease status and time since illness influenced outcome data. Of the 489 patients who were sent baseline questionnaires, 74% returned a follow-up questionnaire on at least one occasion (range one to seven). For the sample of 366 without baseline data for anxiety and depression, 76% returned a follow-up questionnaire on one occasion, whilst only 8% returned a questionnaire on more than one occasion. Outcome measures were also self-reported, and many participants did not complete all measures.

Participant and study characteristics

For peer review only - http://bmjopen.bmj.com/site/about/guidelines.xhtml

Study 1[17] evaluated the effectiveness of the Lightning Process (LP) intervention alongside 'specialist medical care' (SMC) compared with SMC only. Participants were 100 children (mean age of 14) from a UK specialist centre. Study 2[16] sent questionnaires to over 700 patients who had visited the authors' CFS/ME clinic in Australia in the last 20 years, to assess the outcomes of 'routine specialist care'. Table 2 shows participant characteristics.

Both studies measured anxiety and depression, but neither were primary outcomes. Table 2 summarises study characteristics. Study 1 used the Hospital Anxiety and Depression Scale (HADS)[18] and Spence Children's Anxiety Scale (SCAS)[19] to measure anxiety and depression as secondary outcomes. Study 2 measured anxiety and depression at baseline using the State-Trait Anxiety Inventory (STAI)[20] and Beck Depression Inventory (BDI)[21] scales, respectively but there was no repeated measure of anxiety or depression at follow-up points during or after the intervention. Rather, it investigated whether depression and anxiety scores at baseline differed between participants that reported their main outcome of recovery.

In both studies, there were participants who met the criteria for a clinical diagnosis of depression (HADS score > 8[22] or BDI > 20[21]) and anxiety (STAI score > 39[23], HADS score >8) at baseline.

For peer review only

For peer review only - http://bmjopen.bmj.com/site/about/guidelines.xhtml

Table 2: Participant and study characteristics

Author, year, country	Study design	Setting	Sample size		Mean age, years (SD)		Gender (Female %)		CFS/ME diagnostic criteria	Primary Outcome	Secondary Outcomes	Measure of anxiety/ depression	Treatment specifically targeted to anxiety or depression?	Were the outcomes stratified by those with anxiety/ depression?	Intervention	Control	Length of follow up
			Control	Intervention	Control	Intervention	Control	Intervention									
Crawley et al, 2018, UK	RCT	Outpatient, secondary care	49	51	14.5 (1.6)	14.7 (1.4)	78	75	NICE	SF-36 PFS at 6 months	SF-36 PFS at 3 and 12 months; Chalder Fatigue Scale; pain (VAS); anxiety (SCAS and HADS); depression (HADS); school attendance (%); QALY; cost-effectiveness	SCAS, HADS	No	No	Specialist medical care + Lightning Process®	Specialist medical care only	3, 6, 12 months
Rowe et al, 2019, Australia	Observational retrospective	Outpatient, secondary care	418 (789 recruited but 366 did not have baseline questionnaire)		N/A	14.8	N/A	77	CDC/Fukuda	Reported recovery‡ and duration of illness	Bell CFIDS disability scale; global rating*; educational outcomes (proportion of work/school attended, use of educational support, visiting teacher service, educational level achieved); illnesses experiences and exacerbations of CFS/ME; and qualitative feedback†	STAI, BDI	No	No	Routine specialist medical care provided in the outpatient clinic	Nil	Mean: 8 years; Range 1- 21 years

Note: SCAS, Spence Children's Anxiety Scale; HADS, Hospital Anxiety and Depression Scale; STAI, State-Trait Anxiety Inventory; SF-36 PFS, Short-form-36 physical function subscale [27]; VAS, visual analogue scale; QALY, quality-adjusted life-years derived from EQ-5D-Y; Global rating was measured on multiple scales of functioning (incl. school/work, stamina, recovery, social and symptomatology) from 1-10, with 10 being "back to normal"; † qualitative feedback included: what was useful/helpful in treatment, their perceived effectiveness, and whether anything could have been handled better; ‡reported recovery was based on the question "Do you feel you are no longer suffering from CFS?" (yes/no).

For peer review only

Treatment approaches

The LP intervention (https://lightningprocess.com) evaluated in study 1 is developed from osteopathy, life coaching and neurolinguistic programming and more than 250 children use it for their CFS/ME each year in the UK[24]. The intervention in study 2 was routine specialist care delivered at the authors' CFS/ME clinic. Details of the interventions are in Table 3. Neither study offered an intervention that was specifically designed to target anxiety or depression in CFS/ME. Whilst CBT was an element of the 'SMC' and 'routine specialist care' in both studies, we do not know how many children received CBT-f. The differences and similarities between LP and CBT-f are also unclear [25].

For peer review only

For peer review only - http://bmjopen.bmj.com/site/about/guidelines.xhtml

Table 3: Descriptions of treatments in the studies

Crawley et al 2018		Rowe et al 2019
**Lightning Process ®**	**Specialist Medical Care**	**Routine specialist care**
• Three 4-hour sessions on consecutive days run with groups of two to five young people.<br>• Each had a theory session with taught elements on the stress response, how the mind and body interact and how though processes can be either helpful or negative.<br>• Sessions were followed by group discussions where the language used was discussed, and in some cases, challenged, and where participants were encouraged to think about what they could take responsibility for and change.<br>• In the practical session, participants identified a goal they wished to achieve (such as being able to stand up for a longer period of time) and were given different cognitive strategies before and while the goal was attempted.<br>• Participants were also asked to identify a goal to attempt at home.<br>• After the course of sessions, young people were offered at least two follow-up phone calls with a Lighting Process practitioner.	• Based on NICE guidance [1].<br>• Focused on improving sleep and using activity management to establish a baseline level of activity (school, exercise and social) which is then gradually increased.<br>• Sessions delivered by a range of professionals including doctors, psychologists, physiotherapists and occupational therapists in family-based rehabilitation consultations.<br>• The number and timing of sessions were agreed with the family depending on each adolescents' needs and goals.<br>• Those with significant anxiety or low mood were offered additional CBT.	A person-centred goal-oriented holistic program which targets educational, physical, social and emotional aspects of life. It included:<br>• An initial appointment where the young person identifies and rates symptoms they would like help with, outlines their aspirations and is given explanations of illness and management plans available.<br>• Development of a management plan in collaboration with parent, child and clinician which aims to minimise impact of chronic illness while accommodating for specifics of CFS.<br>• A focus on physical social and emotional aspects including proactive social contact, academic input, physical activity and a commitment to something enjoyable outside the home on a regular basis.<br>• An explanation that the consequence of illness can be more damaging than the illness itself and tools on how to navigate this.<br>• Symptom management e.g. sleep, migraine, dizziness, nausea, orthostatic intolerance, concentration difficulties.<br>• 6-week review appointment to review management plans and change if necessary.

Key: CFS, chronic fatigue syndrome; CBT, cognitive behavioural therapy

For peer review only

For peer review only - http://bmjopen.bmj.com/site/about/guidelines.xhtml

Treatment efficacy

Study 1 showed the LP resulted in a reduction in depression symptoms across *both* the intervention (LP+SMC) and control (SMC) groups, but there was a greater difference reduction in symptoms (based on adjusted mean differences) among participants allocated to the LP+SMC intervention than those allocated to the SMC control. This difference was only statistically significant at the later follow-up time-point of 12 months, not earlier at 6 months. The study showed LP was more effective at reducing anxiety symptoms compared with depression (at both 6 and 12 months follow-up). However, the reduction in anxiety symptoms differed depending on whether they were measured using the HADS or SCAS: at 6 months follow-up, there was a reduction in anxiety symptomatology as measured by both HADS and SCAS, but at the full 12 months follow-up, the improvements in the HADS anxiety score were smaller than when measured by SCAS. But, at both time-points participant numbers were small (43/51 participants at 12 months; 46/51 at 6 months). Outcomes in this study were not stratified by those with depression or anxiety, so we cannot comment on other CFS/ME outcomes in context of comorbid depression or anxiety.

Study 2 measured depressive and anxiety symptomatology at baseline but not post-treatment, so we cannot comment on the effectiveness of their intervention at reducing depression or anxiety. Instead, they compared mean baseline depression and anxiety scores between those who had self-reported 'recovery', defined as answering "yes" to the question "Do you feel you are no longer suffering from CFS?" measured at a mean length of follow-up of 8 years (range 1-21). There was no difference in depression or anxiety at baseline between those who reported that they had recovered and those who had not i.e. depression nor anxiety were found to be associated with recovery.

For peer review only - http://bmjopen.bmj.com/site/about/guidelines.xhtml

Both studies reported improvements in other CFS/ME outcomes following intervention, including physical function, fatigue, and self-reported “recovery”. Table 4 shows the summary of outcomes of depression and anxiety and other relevant findings for each included study.

For peer review only

Table 4: Summary of outcomes for symptoms of depression and anxiety and other relevant findings for included studies

Author, Year, Country	Measure of Depression and Anxiety	Pre treatment: depression, mean (SD)		Pre treatment: anxiety, mean (SD)		Post treatment: depression, mean		Post treatment: anxiety, mean		Statistical analysis of change in depression/anxiety symptomatology		Summary of other relevant findings
		Intervention	Control	Intervention	Control	Intervention	Control	Intervention	Control	Depression	Anxiety	
**Crawley et al. 2018. UK**	HADS* (depression and anxiety scales),<br><br>SCAS* (anxiety scale)	7.5 (3.1)	8.1 (4.4)	HADS: 8.8 (4.5)<br><br>SCAS: 29.8 (16.9)	HADS: 10.4 (4.4)<br><br>SCAS: 40.3 (20.1)	6 months: 4.2<br><br>12 months: 2.8	6 months: 5.9<br><br>12 months: 4.6	HADS<br>6 months: 6.1<br>12 months: 5.3<br><br>SCAS<br>6 months: 24.7<br>12 months: 19.6	HADS<br>6 months: 9.7<br>12 months: 8.3<br><br>SCAS<br>6 months: 37.4<br>12 months: 36.3	Adjusted difference in means† (95%CI, pvalue):<br><br>6 months:<br>-1.5 (-3.5 to 0.5, p=0.1)<br><br>12 months:<br>-1.8 (-3.4 to -0.1, p=0.04)	Adjusted difference in means† (95%CI, pvalue):<br><br>HADS at 6 months:<br>-3.5 (-5.6 to -1.5, p=0.001)<br><br>SCAS at 6 months:<br>-10.0 (-18.5 to -1.5, p=0.02)<br><br>HADS at 12 months:<br>-2.6 (-4.7 to -0.4, p=0.019);<br><br>SCAS at 12 months:<br>14.5 (-22.4 to -6.7, p=)	At 6 months, participants allocated to LP in addition to SMC (intervention) had better physical function and fatigue at than those allocated to SMC (control).<br><br>At 12 months, participants allocated to LP in addition to SMC (intervention) had better fatigue and school attendance than those in SMC (control).<br><br>Adding LP to SMC is cost-effective.
**Rowe et al. 2019. Australia**	BDI* (depression scale),<br><br>STAI* (anxiety scale)	13.8 (8.9)	N/A	88.9 (24.9)	N/A	N/A	N/A	N/A	N/A	No statistical change because post-treatment scores were not measured. Instead, mean baseline depression and anxiety scores were compared between those who reported recovery‡ and those who did not, using the student's t-test.		Overall, 46.5% reported recovery; participants who were followed for >10 years, 68% reported recovery<br><br>Mean duration of illness was 5 years

Note: *higher score=more symptoms, poorer function; † adjusted for age, gender, baseline outcome, SCAS and visual analogue scale; ‡reported recovery was based on the question "Do you feel you are no longer suffering from CFS?" (yes/no).

HADS, Hospital Anxiety and Depression Scale (score >8 indicates a diagnosis of depression); SCAS, Spence Children's Anxiety Scale ; BDI, Beck's Depression Inventory (score >20 indicates moderate depression); STAI, State Trait Anxiety index; LP, Lightning Process; SMC, Specialist Medical Care

For peer review only

DISCUSSION

Our updated review of interventions for comorbid depression and/or anxiety in children with CFS/ME identified only two new studies published since 2015 (one of which was conducted by members of our own research team), exposing the lack of progress in this field. Neither study specifically targeted comorbid anxiety and/or depression. Study 1 showed adding LP to SMC was more effective than SMC alone at reducing both depressive and, to a greater extent, anxiety symptoms. Study 2 did not measure depression or anxiety at follow-up. Study 1 had a small sample size and both studies suffered from bias.

Strengths of this review include the systematic approach, the use of four reviewers, contacting authors for sub-group data, and not limiting results to English language. The limitations are the lack of eligible studies and insufficient data available for a meta-analysis. Only two papers were eligible for inclusion, of which one did not provide sufficient follow-up data to comment on the treatment efficacy of the intervention on depression and anxiety. Neither intervention was specifically designed to measure the impact on depression and anxiety and therefore studies were inadequately powered to measure this. Studies were not stratified by those who met criteria for clinical diagnoses of depression/anxiety reducing our ability to analyse effectiveness. Furthermore, neither study used diagnostic interviews for anxiety and depression, relying instead on questionnaires. Whilst HADS[26], SCAS[27], and STAI[20] questionnaires are validated for use in adolescents, only the RCADS (Revised Children’s Anxiety and Depression scale), which is derived from the SCAS, has been found to have sufficient discriminative accuracy against gold standard diagnostic interviews in paediatric CFS/ME populations[5].

For peer review only - http://bmjopen.bmj.com/site/about/guidelines.xhtml

To date, the intervention with the most evidence for improvement in anxiety and depressive symptoms in CFS/ME, when compared to other interventions, such as behavioural-only or pharmacological, is CBT-f[10, 11]. The mechanisms for why CBT-f is effective are unclear because no study targeted anxiety and depression. Our study does not further this debate as the only trial that measured anxiety and depression at follow-up (study 1) did not clearly report whether CBT-f was delivered in the control (SMC) arm.

Other cognitive and behavioural based approaches are being trialled in CFS/ME, but are limited in contributing to our understanding of their efficacy for anxiety and depressive symptoms in CFS/ME because of a failure to include paediatric CFS/ME populations or those diagnosed with CFS/ME using recognised criteria, or measure anxiety and depressive symptoms in the 20-30%[5, 6] of children that experience them. Three studies[28-30] were excluded from our review for these reasons. For example, studies evaluating Acceptance and Commitment Therapy[28] and Mindfulness-based therapies[29] show promising results in improving the physical health, symptom burden and 'emotional distress' in children with functional somatic syndromes including CFS/ME but were excluded from this review because data for adolescent participants with CFS/ME were aggregated with those with other somatic syndromes, and the studies only measured general wellbeing outcomes rather than specifically validated anxiety and/or depression outcomes.

There is a pressing need for more work in this area to identify efficacious treatments for anxiety and depressive symptoms in paediatric CFS/ME so they can be used in clinical practice. We call upon researchers to undertake paediatric CFS/ME interventions studies and use validated, diagnostic outcome measures of anxiety and depression.

For peer review only - http://bmjopen.bmj.com/site/about/guidelines.xhtml

CONCLUSION

This review highlights both the paucity of intervention studies in children with CFS/ME and the lack of forward movement in identifying effective treatments for paediatric CFS/ME and comorbid depression and anxiety over the last five years. Calls for paediatric CFS/ME intervention studies to target anxiety and depression, measure outcomes with validated scales, or report outcomes in subsets of patients with clinical diagnoses of anxiety and depression, have not been met. The LP in addition to SMC appears to be effective at reducing depressive and anxiety symptoms, but this is only one study and findings have not been replicated, and it is unclear whether changes are sustained long-term. Given that comorbid anxiety and depression in paediatric CFS/ME are associated with worse outcomes, unlikely to remit spontaneously without treatment, and can be incompatible with following standard CFS/ME treatment guidance, it remains a priority to focus on these outcomes in future research.

ACKNOWLEDGEMENTS

We would like to acknowledge the support from the CFS/ME teams at the Centre for Academic Child Health at the University of Bristol and the CFS/ME service at the Royal United Hospitals Bath NHS Foundation Trust.

AUTHOR CONTRIBUTIONS

ML and EC conceptualised this study. PC, AR, KD, and JB performed data collection, synthesis and interpretation. PC wrote the manuscript. All authors contributed to manuscript revisions, have read the final manuscript and approved it for publication. All authors agree to be accountable for all aspects of the work.

For peer review only - http://bmjopen.bmj.com/site/about/guidelines.xhtml

COMPETING INTERESTS STATEMENT

Professor Crawley acts as a non-paid medical advisor for the Sussex and Kent ME society.

FUNDING STATEMENT

Dr Loades is funded by the National Institute for Health Research (NIHR Doctoral Research Fellowship, DRF-2016-09-021). This report is independent research. The views expressed in this publication are those of the authors and not necessarily those of the NHS, NIHR or the Department of Health and Social Care.

DATA STATEMENT

Not applicable.

REFERENCES

1. NICE. Chronic fatigue syndrome/myalgic encephalomyelitis (or encephalopathy): diagnosis and management (CG53). 2007 [Accessed February 2020]. Available from: https://www.nice.org.uk/guidance/CG53.

2. Lim E, Ahn Y, Jang E, et al. Systematic review and meta-analysis of the prevalence of chronic fatigue syndrome/myalgic encephalomyelitis (CFS/ME). *J Transl Med*, 2020;18(100) doi:10.1186/s12967-020-02269-0

For peer review only - http://bmjopen.bmj.com/site/about/guidelines.xhtml

3. Crawley E and Sterne JA. Association between school absence and physical function in paediatric chronic fatigue syndrome/myalgic encephalopathy. *Arch Dis Child*, 2009;94(10):752-6 doi:10.1136/adc.2008.143537

4. Falk Hvidberg M, Brinth LS, Olesen AV, et al. The Health-Related Quality of Life for Patients with Myalgic Encephalomyelitis / Chronic Fatigue Syndrome (ME/CFS). *PloS One* 2015;10(7) doi:10.1371/journal.pone.0132421

5. Loades ME, Read R, Smith L, et al. How common are depression and anxiety in adolescents with chronic fatigue syndrome (CFS) and how should we screen for these mental health co-morbidities? A clinical cohort study. *Eur Child Adolesc Psychiatry* 2020 doi:10.1007/s00787-020-01646-w

6. Loades ME, Rimes K, Ali S, et al. The presence of co-morbid mental health problems in a cohort of adolescents with chronic fatigue syndrome. *Clin Child Psychol Psychiatry* 2018;23(3): 398-408 doi:10.1177/1359104517736357

7. Sadler K, Vizard T, Ford T, et al. Mental Health of Children and Young People in England, 2017: Trends and characteristics. [Accessed February 2020]. Available from: https://digital.nhs.uk/data-and-information/publications/statistical/mental-health-of-children-and-young-people-in-england/2017/2017

8. Bould H, Collin S, Lewis G, et al. Depression in paediatric chronic fatigue syndrome. *Arch Dis Child* 2013;98(6):425-8 doi:10.1136/archdischild-2012-303396

9. Loades ME, Rimes K, Ali S, et al. Depressive symptoms in adolescents with chronic fatigue syndrome (CFS): Are rates higher than in controls and do depressive symptoms affect outcome? *Clin Child Psychol Psychiatr*y 2019;24(3):580-592 doi:10.1177/1359104519838

10. Loades, ME, Sheils EA and Crawley E. Treatment for paediatric chronic fatigue syndrome or myalgic encephalomyelitis (CFS/ME) and comorbid depression: a systematic review. *BMJ Open* 2016;6(10) doi:10.1136/bmjopen-2016-012271

For peer review only

11. Stoll S, Crawley E, Richards V, et al. What treatments work for anxiety in children with chronic fatigue syndrome/myalgic encephalomyelitis (CFS/ME)? Systematic review. *BMJ open* 2017;7(9) doi:10.1136/bmjopen-2016-015481

12. Moher D, Liberati A, Tetzlaff J, et al. Preferred reporting items for systematic reviews and meta-analyses: the PRISMA statement. *PLoS Med* 2009;6(7) doi:10.1136/bmj.b2535

13. Sterne J, Hernan M, Reeves B, et al. ROBINS-I: a tool for assessing risk of bias in non-randomised studies of interventions. *BMJ* 2016;355 doi:10.1136/bmj.i4919

14. Sterne J, Savović J, Page M, et al. RoB 2: a revised tool for assessing risk of bias in randomised trials. *BMJ* 2019;366 doi:10.1136/bmj.l4898

15. Campbell M, McKenzie J, Sowden A, et al. Synthesis without meta-analysis (SWiM) in systematic reviews: reporting guideline. *BMJ* 2020;368 doi:10.1136/bmj.l6890

16. Rowe K. Long Term Follow up of Young People With Chronic Fatigue Syndrome Attending a Pediatric Outpatient Service. *Frontiers in pediatrics* 2019;7(21) doi:10.3389/fped.2019.00021

17. Crawley E, Gaunt D, Garfield K, et al. Clinical and cost-effectiveness of the Lightning Process in addition to specialist medical care for paediatric chronic fatigue syndrome: randomised controlled trial. *Arch Dis Child* 2018;103(2):155-164 doi:10.1136/archdischild-2017-313375

18. Zigmond AS and Snaith RP. The hospital anxiety and depression scale. *Acta Psychiatr Scand* 1983;67(6):361-70 doi:10.1111/j.1600-0447.1983.tb09716.x

19. Spence SH, Barrett PM and Turner CM. Psychometric properties of the Spence Children's Anxiety Scale with young adolescents. *J Anxiety Disord* 2003;17(6):605-625 doi:10.1016/s0887-6185(02)00236-0

20. Spielberger CD. Manual for the state trait anxiety inventory for children. Palo Alto: Consulting Psychologists Press 1973.

21. Beck AT, Steer RA and Carbin MG. Psychometric properties of the Beck Depression Inventory: Twenty-five years of evaluation. *Clin Psychol Rev* 1988;8(1):77-100 doi:10.1016/0272-7358(88)90050-5

For peer review only - http://bmjopen.bmj.com/site/about/guidelines.xhtml

22. Bjelland I, Dahl A, Haug T, et al. The validity of the Hospital Anxiety and Depression Scale: An updated literature review. *J Psychosom Res* 2002;52(2):69-77 doi:10.1016/s0022-3999(01)00296-3

23. Julian LJ. Measures of anxiety: State-Trait Anxiety Inventory (STAI), Beck Anxiety Inventory (BAI), and Hospital Anxiety and Depression Scale-Anxiety (HADS-A). *Arthritis Care & Research* 2011;63(S11):S467-472 doi:10.1002/acr.20561

24. Julian LJ. Measures of anxiety: State-Trait Anxiety Inventory (STAI), Beck Anxiety Inventory (BAI), and Hospital Anxiety and Depression Scale-Anxiety (HADS-A). *Arthritis Care & Research* 2011;63(S11):S467-472 doi:10.1002/acr.20561

25. Anderson E, Loades M, Starbuck J, et al. CBT repackaged or a novel treatment? The Lightning Process® compared with specialist medical care for paediatric Chronic Fatigue Syndrome. Submitted 2020.

26. White D, Leach C, Sims R, et al. Validation of the Hospital Anxiety and Depression Scale for use with adolescents. *Br J Psychiatry* 1999;(175):452-454 doi:10.1192/bjp.175.5.452

27. Orgilés M, Fernández-Martínez I, Guillén-Riquelme A, et al. A systematic review of the factor structure and reliability of the Spence Children's Anxiety Scale. *J Affect Disord* 2016(190):333-340 doi:10.1016/j.jad.2015.09.055

28. Kallesøe K, Schröder A, Wicksell R, et al. Feasibility of group-based acceptance and commitment therapy for adolescents (AHEAD) with multiple functional somatic syndromes: a pilot study. *BMC Psychiatry* 2020;20(1):457 doi:10.1186/s12888-020-02862-z

29. Ali A, Weiss T, Dutton A, et al. Mindfulness-Based Stress Reduction for Adolescents with Functional Somatic Syndromes: A Pilot Cohort Study. *J Pediatr* 2017;183:184-190 doi:10.1016/j.jpeds.2016.12.053

30. Kluck BN, Junghans-Rutelonis AN, Jones AE, et al. Adolescent Chronic Fatigue and Orthostatic Intolerance. *Clinical Pediatrics* 2017;56(1):85-89 doi:10.1177/0009922816644730

For peer review only - http://bmjopen.bmj.com/site/about/guidelines.xhtml

FIGURES AND TABLES LEGENDS

Figure 1: Flow chart for studies included in the systematic review; based on PRISMA guidelines

Table 1: Inclusion criteria

Table 2: Participant and study characteristics

Table 3: Descriptions of treatments in the studies

Table 4: Summary of outcomes for symptoms of depression and anxiety and other relevant findings for included studies

For peer review only

For peer review only - http://bmjopen.bmj.com/site/about/guidelines.xhtml

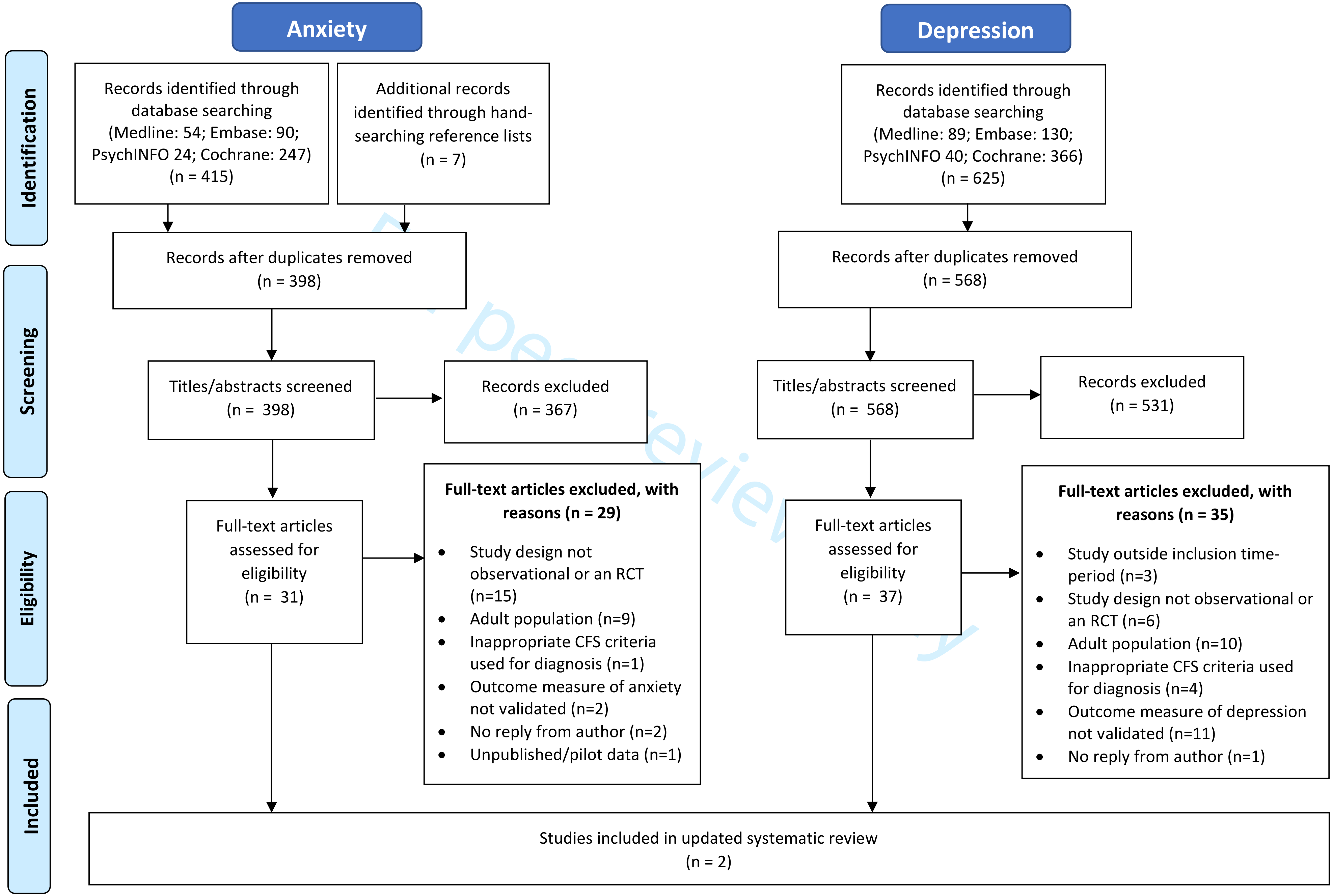

Key: CFS, chronic fatigue syndrome; ME, myalgic encephalomyelitis; PRISMA, Preferred Reporting Items for Systematic Reviews and Meta-Analyses; RCT, randomised controlled trials.

For peer review only - http://bmjopen.bmj.com/site/about/guidelines.xhtml

Search Strategies

Search strategy for Anxiety searches:

1. (adolesc* or preadolesc* or pre-adolesc* or boy* or girl* or child* or infan* or preschool* or pre-school* or juvenil* or minor* or pe?diatri* or pubescen* or pre-pubescen* or prepubescen* or puberty or teen* or young* or youth* or school* or high-school* or highschool* or sibling* or schoolchild* or school child* or children).tw.
2. exp Adolescent/ or exp Child/ or exp Child, Preschool/ or exp Infant/ or exp Minors/ or exp Pediatrics/
3. 1 or 2
4. Chronic Fatigue Syndrome.tw
5. myalgic encephal*.tw.
6. chronic fatigue syndrome*.mp.
7. myalgic encephal*.mp.
8. anxiety disorder/
9. exp anxiety disorder
10. exp obsessive-compulsive disorder
11. exp panic
12. anxi*.tw
13. generali#ed anxiety disorder.tw
14. obsessive compulsive.tw
15. OCD.tw
16. Phobia*.tw
17. Social anxiety.tw
18. Separation anxiety.tw
19. Panic.tw

For peer review only - http://bmjopen.bmj.com/site/about/guidelines.xhtml

20. exp Chronic Fatigue Syndrome/
21. exp Anxiety Disorders/ or exp Social Phobia/ or exp Panic Disorder/ or exp Anxiety/ or exp Social Anxiety
22. exp Separation Anxiety Disorder/ or Separation Anxiety/
23. exp Generalized Anxiety Disorder
24. exp Obsessive Compulsive Disorder
25. exp Phobias/
26. 4 or 5 or 6 or 7 or 20
27. 8 or 9 or 10 or 11 or 12 or 13 or 14 or 15 or 16 or 17 or 18 or 19 or 21 or 22 or 23 or 24 or 25
28. 3 and 26 and 27
29. Limit 28 to yr="2016-current"

Search strategy for Depression searches:

1. (adolesc* or preadolesc* or pre-adolesc* or boy* or girl* or child* or infan* or preschool* or pre-school* or juvenil* or minor* or pe?diatri* or pubescen* or pre-pubescen* or prepubescen* or puberty or teen* or young* or youth* or school* or high-school* or highschool* or sibling* or schoolchild* or school child* or children).tw.
2. exp Adolescent/ or exp Child/ or exp Child, Preschool/ or exp Infant/ or exp Minors/ or exp Pediatrics/
3. 1 or 2
4. chronic fatigue syndrome*.mp.
5. exp Chronic Fatigue Syndrome
6. Chronic Fatigue Syndrome.tw
7. myalgic encephal*.mp.
8. myalgic encephal*.tw.

For peer review only - http://bmjopen.bmj.com/site/about/guidelines.xhtml

9. 4 or 5 or 6 or 7 or 8
10. depressive disorder.mp.
11. exp depression/
12. depress*.tw
13. dysthymi*.tw
14. exp adjustment disorders/
15. adjustment disorder* .mp.
16. low mood.tw.
17. 10 or 11 or 12 or 14 or 14 or 15 or 16
18. 3 and 9 and 17
19. Limit 18 to yr = “2015 – current”

For peer review only

For peer review only - http://bmjopen.bmj.com/site/about/guidelines.xhtml

PRISMA 2009 Checklist

Section/topic	#	Checklist item	Reported on page #
**TITLE**			
Title	1	Identify the report as a systematic review, meta-analysis, or both.	1
**ABSTRACT**			
Structured summary	2	Provide a structured summary including, as applicable: background; objectives; data sources; study eligibility criteria, participants, and interventions; study appraisal and synthesis methods; results; limitations; conclusions and implications of key findings; systematic review registration number.	2-3
**INTRODUCTION**			
Rationale	3	Describe the rationale for the review in the context of what is already known.	4-5
Objectives	4	Provide an explicit statement of questions being addressed with reference to participants, interventions, comparisons, outcomes, and study design (PICOS).	5
**METHODS**			
Protocol and registration	5	Indicate if a review protocol exists, if and where it can be accessed (e.g., Web address), and, if available, provide registration information including registration number.	3
Eligibility criteria	6	Specify study characteristics (e.g., PICOS, length of follow-up) and report characteristics (e.g., years considered, language, publication status) used as criteria for eligibility, giving rationale.	Table 1 page 6
Information sources	7	Describe all information sources (e.g., databases with dates of coverage, contact with study authors to identify additional studies) in the search and date last searched.	5-6
Search	8	Present full electronic search strategy for at least one database, including any limits used, such that it could be repeated.	Supplementary
Study selection	9	State the process for selecting studies (i.e., screening, eligibility, included in systematic review, and, if applicable, included in the meta-analysis).	7
Data collection process	10	Describe method of data extraction from reports (e.g., piloted forms, independently, in duplicate) and any processes for obtaining and confirming data from investigators.	7
Data items	11	List and define all variables for which data were sought (e.g., PICOS, funding sources) and any assumptions and simplifications made.	6
Risk of bias in individual studies	12	Describe methods used for assessing risk of bias of individual studies (including specification of whether this was done at the study or outcome level), and how this information is to be used in any data synthesis.	8-9
Summary measures	13	State the principal summary measures (e.g., risk ratio, difference in means).	7

For peer review only

PRISMA 2009 Checklist

Synthesis of results	14	Describe the methods of handling data and combining results of studies, if done, including measures of consistency (e.g., I^2) for each meta-analysis.	N/A

Section/topic	#	Checklist item	Reported on page #
Risk of bias across studies	15	Specify any assessment of risk of bias that may affect the cumulative evidence (e.g., publication bias, selective reporting within studies).	9
Additional analyses	16	Describe methods of additional analyses (e.g., sensitivity or subgroup analyses, meta-regression), if done, indicating which were pre-specified.	N/A
**RESULTS**			
Study selection	17	Give numbers of studies screened, assessed for eligibility, and included in the review, with reasons for exclusions at each stage, ideally with a flow diagram.	8
Study characteristics	18	For each study, present characteristics for which data were extracted (e.g., study size, PICOS, follow-up period) and provide the citations.	9-10and Table 2
Risk of bias within studies	19	Present data on risk of bias of each study and, if available, any outcome level assessment (see item 12).	8-9
Results of individual studies	20	For all outcomes considered (benefits or harms), present, for each study: (a) simple summary data for each intervention group (b) effect estimates and confidence intervals, ideally with a forest plot.	Table 4
Synthesis of results	21	Present results of each meta-analysis done, including confidence intervals and measures of consistency.	N/A
Risk of bias across studies	22	Present results of any assessment of risk of bias across studies (see Item 15).	8-9
Additional analysis	23	Give results of additional analyses, if done (e.g., sensitivity or subgroup analyses, meta-regression [see Item 16]).	N/A
**DISCUSSION**			
Summary of evidence	24	Summarize the main findings including the strength of evidence for each main outcome; consider their relevance to key groups (e.g., healthcare providers, users, and policy makers).	17-18
Limitations	25	Discuss limitations at study and outcome level (e.g., risk of bias), and at review-level (e.g., incomplete retrieval of identified research, reporting bias).	17
Conclusions	26	Provide a general interpretation of the results in the context of other evidence, and implications for future research.	17-18
**FUNDING**			
Funding	27	Describe sources of funding for the systematic review and other support (e.g., supply of data); role of funders for the systematic review.	20

For peer review only

PRISMA 2009 Checklist

From: Moher D, Liberati A, Tetzlaff J, Altman DG, The PRISMA Group (2009). Preferred Reporting Items for Systematic Reviews and Meta-Analyses: The PRISMA Statement. PLoS Med 6(7): e1000097. doi:10.1371/journal.pmed1000097

For more information, visit: **www.prisma-statement.org**.

For peer review only
For peer review only - http://bmjopen.bmj.com/site/about/guidelines.xhtml

What treatments work for anxiety and depression in children and adolescents with Chronic Fatigue Syndrome? An updated systematic review

Journal:	*BMJ Open*
Manuscript ID	bmjopen-2021-051358.R1
Article Type:	Original research
Date Submitted by the Author:	24-Jul-2021
Complete List of Authors:	Clery, Philippa; University of Bristol, Centre for Academic Child Health<br>Royston, Alexander; University of Bristol, Centre for Academic Child Health<br>Driver, Katie; University of Bristol, Centre for Academic Child Health<br>Bailey, Jasmine; University of Bristol, Centre for Academic Child Health<br>Crawley, Esther; University of Bristol, Centre for Academic Child Health; Royal United Hospitals Bath NHS Foundation Trust, Paediatric Chronic Fatigue Syndrome Specialist Service<br>Loades, Maria; University of Bristol, Centre for Academic Child Health; University of Bath, Department of Psychology
**Primary Subject Heading**:	Paediatrics
Secondary Subject Heading:	Paediatrics
Keywords:	PAEDIATRICS, Anxiety disorders < PSYCHIATRY, Depression & mood disorders < PSYCHIATRY, MENTAL HEALTH, Child & adolescent psychiatry < PSYCHIATRY

SCHOLARONE™
Manuscripts

I, the Submitting Author has the right to grant and does grant on behalf of all authors of the Work (as defined in the below author licence), an exclusive licence and/or a non-exclusive licence for contributions from authors who are: i) UK Crown employees; ii) where BMJ has agreed a CC-BY licence shall apply, and/or iii) in accordance with the terms applicable for US Federal Government officers or employees acting as part of their official duties; on a worldwide, perpetual, irrevocable, royalty-free basis to BMJ Publishing Group Ltd ("BMJ") its licensees and where the relevant Journal is co-owned by BMJ to the co-owners of the Journal, to publish the Work in this journal and any other BMJ products and to exploit all rights, as set out in our licence.

The Submitting Author accepts and understands that any supply made under these terms is made by BMJ to the Submitting Author unless you are acting as an employee on behalf of your employer or a postgraduate student of an affiliated institution which is paying any applicable article publishing charge ("APC") for Open Access articles. Where the Submitting Author wishes to make the Work available on an Open Access basis (and intends to pay the relevant APC), the terms of reuse of such Open Access shall be governed by a Creative Commons licence – details of these licences and which Creative Commons licence will apply to this Work are set out in our licence referred to above.

Other than as permitted in any relevant BMJ Author's Self Archiving Policies, I confirm this Work has not been accepted for publication elsewhere, is not being considered for publication elsewhere and does not duplicate material already published. I confirm all authors consent to publication of this Work and authorise the granting of this licence.

For peer review only

What treatments work for anxiety and depression in children and adolescents with Chronic Fatigue Syndrome? An updated systematic review

Philippa Clery[1], Alexander Royston[1], Katie Driver[1], Jasmine Bailey[1], Esther Crawley[1,2], Maria Loades[1,3]

[1] Centre for Academic Child Health, Bristol Medical School, Bristol, UK

[2] Paediatric Chronic Fatigue Syndrome Specialist Service, Royal United Hospitals Bath NHS Foundation Trust, Bath, UK

[3] Department of Psychology, University of Bath, Bath, UK

Corresponding author:

Dr Philippa Clery

Centre for Academic Child Health, 1-5 Whiteladies Road, Bristol, BS8 1NU, UK.

Philippa.clery@bristol.ac.uk

Word count: 3545

For peer review only - http://bmjopen.bmj.com/site/about/guidelines.xhtml

ABSTRACT

Objectives

Children with Chronic Fatigue Syndrome/Myalgic Encephalomyelitis (CFS/ME) experience a higher prevalence of depression and anxiety compared to age-matched controls. Our previous systematic reviews in 2015/16 found little evidence for effective treatment for children with CFS/ME with comorbid depression and/or anxiety. This review updates these findings.

Design

A systematic review. We searched Cochrane library, Medline, Embase and PsychINFO databases from 2015-2020. We combined the updated results with our previous reviews in a narrative synthesis.

Participants

Inclusion criteria: <18 years old; diagnosed with CFS/ME (using Centre for Disease Control, National Institute for Health and Care Excellence, or Oxford criteria); validated measures of depression and/or anxiety.

Interventions

Observational studies or randomised controlled trials.

Comparison

Any or none.

Outcomes

Studies with outcome measures of anxiety, depression, or fatigue.

Results

For peer review only

The updated review identified two studies. This brings the total number of paediatric CFS/ME studies with a measure of anxiety and/or depression since 1991 to 16. None of the studies specifically targeted depression, nor anxiety. One new study showed the Lightning Process (in addition to specialist care) was more effective at reducing depressive and anxiety symptoms compared to specialist care alone. Previous studies evaluated cognitive behavioural therapy (CBT); pharmacological interventions; and behavioural approaches. CBT-type interventions had most evidence for improving comorbid anxiety and/or depressive symptoms but varied in delivery and modality. Other interventions showed promise but studies were small and have not been replicated.

Conclusion

Very few paediatric CFS/ME intervention studies have been conducted. This review update does not significantly add to what is known from previous reviews. The evidence is of poor quality and insufficient to conclude which interventions are effective at treating comorbid anxiety and/or depression in paediatric CFS/ME.

Trial registration number

Reviews are registered on the Prospective Register of Systematic Review Protocols:

http://www.crd.york.ac.uk/PROSPERO/display_record.asp?ID=CRD42016043488 ;

http://www.crd.york.ac.uk/PROSPERO/display_record.asp?ID=CRD42015016813.

Key words

Paediatric, CFS/ME, chronic fatigue syndrome, anxiety, depression

ARTICLE SUMMARY

Strengths and limitations of study

For peer review only

- This review used a systematic approach to identify updated evidence for treatment approaches for comorbid anxiety and/or depression in paediatric CFS/ME, and combined it with previous review results to provide a comprehensive synthesis of all evidence.
- Non-English language articles were included.
- Authors were contacted and sub-group data obtained when available.
- Grey literature and unpublished material was not included.
- There was insufficient data to carry out a meta-analysis.

INTRODUCTION

Chronic fatigue syndrome (CFS)/myalgic encephalomyelitis (ME) is a common but poorly understood condition causing disabling fatigue, malaise, myalgia, sleep difficulties, and problems concentrating[1]. In children and adolescents (henceforth referred to as children), prevalence is estimated at 0.55% (95%CI 0.22-1.35) across community, primary care and hospital populations[2]. CFS/ME has long-term impacts on children's physical, cognitive, emotional and social functioning[3, 4].

Children with CFS/ME suffer from higher rates of both depression and anxiety than age-matched population samples. The prevalence estimates of comorbid depression and anxiety are 20%[5] and 29%[6], respectively, compared to 2.1% and 7.2%[7] in adolescents without CFS/ME. In those attending a specialist CFS/ME service, 61% who meet diagnostic criteria for depression also have an anxiety disorder[5]. Having comorbid depression and/or anxiety is associated with less favourable outcomes and may impact on engaging with treatment. Comorbid depression in paediatric CFS/ME is associated with greater functional disability, worse fatigue and more pain compared with those without depression[8, 9]. Low mood, anergia and anhedonia could be barriers to motivation to

For peer review only

engage in behavioural treatment approaches and Cognitive Behavioural Therapy-for-fatigue (CBT-f). Depressive symptoms are therefore likely to require tailored treatment[9]. The impact of anxiety on outcomes is less clear. Given that most children with CFS/ME who have anxiety also have depression[5], it is important to explore treatments for both.

Despite the high prevalence of comorbid mental health problems, there is little evidence about the effectiveness of treatments. Our two previous systematic reviews looking at depression and anxiety outcomes in existing CFS/ME intervention studies found that no specifically adapted treatments had been trialled to target depression and anxiety in paediatric CFS/ME[10, 11]. Although CBT-f and a multicomponent inpatient programme showed promise in reducing depressive[10] and anxiety[11] symptoms, there was no consistent treatment approach for children with CFS/ME and comorbid depression or anxiety. Since conducting these reviews in 2015/16, further intervention studies may have been published. It is important and timely to review the current evidence to provide an update on what treatments should be offered to this population. Further, it is important to consider anxiety and depression together given their overlap, whereas our previous reviews considered them separately.

We conducted an updated systematic review by synthesizing the evidence regarding treatments for paediatric CFS/ME and comorbid depression and anxiety since 2015. We combined these findings with results from our previous systematic reviews (1991-2015) to give an overview of all interventions evaluated since 1991 (when CFS/ME was scientifically defined). Specifically, we aimed to address the following:

1. What treatment approaches are there for depression and anxiety in children with CFS/ME?

For peer review only - http://bmjopen.bmj.com/site/about/guidelines.xhtml

2. What is known about the treatment efficacy of these approaches for treating depression and anxiety in CFS/ME? Do different approaches have different outcomes?

METHODS

Data sources and search strategy

We conducted searches on Medline, Embase, PsychINFO and Cochrane Library databases. We used the same search strategies from the previous systematic reviews (registered on Prospero: CRD42015016813; CRD42016043488) to repeat the depression and anxiety searches separately. Searches were designed with input from an information specialist to include the concepts: paediatric; CFS/ME; anxiety and depression (search strategies are in supplementary material). We updated the searches from when they had last been run (February 2015 for depression search and July 2016 for anxiety search) up until September 2020. The two searches were carried out by different reviewer teams: anxiety search (PC, AR); depression search (KD, JB). Grey literature was not searched. Reference lists of articles for full-text screening were hand-searched.

Inclusion and exclusion Criteria

Studies were included if they met inclusion criteria (Table 1).

Table 1: Inclusion criteria

	Anxiety Review	Depression Review
**Participants**	1. Children <18 years of age<br>2. Diagnosed with CFS/ME defined using one of these criteria:<br>CDC aka Fukuda[12]<br>NICE[1]<br>Oxford aka Sharpe[13]	

For peer review only

**Interventions**	Observational cohort studies<br>Any study with intervention – e.g., observational clinical cohorts, clinical trials, etc.	
**Baseline measure**	Validated assessment of anxiety	Validated assessment of depression
**Outcome measure**	**Either** an anxiety **and/or** fatigue measure on psychometrically validated assessments or validated diagnostic interviews.	**Either** a depression **and/or** fatigue measure on psychometrically validated assessments or validated diagnostic interviews.
**Language**	Non-English language papers were considered for inclusion.	

Study selection

Articles returned from database searches were inputted into Endnote and duplicates removed. Each reviewer conducted title and abstract screening independently. Full texts of potentially eligible articles were screened against specifically created eligibility checklists. The final articles for inclusion were cross-checked between all four reviewers and any conflicts discussed and resolved with input from the senior author (ML) if necessary. Where information from the paper was insufficient to determine eligibility, authors were contacted by email for additional information. If authors did not reply after two follow-up emails, the study was excluded. Figure 1 presents the PRISMA[14] flowchart.

Data extraction

For all included articles, data were extracted independently by two reviewers (PC, AR) using a purpose-designed data extraction form to collect information about: study design; setting; recruitment; participant characteristics; CFS/ME definition used for diagnosis; assessment of depression and anxiety; other outcomes; treatment and interventions provided; definition of response and treatment/intervention outcomes.

For peer review only

Quality assessment

PC and AR used Risk of Bias assessment tools[15, 16] to assess methodological quality of the included studies.

Data synthesis

We combined results from the included studies identified in the updated search with findings from the two previous systematic reviews[10, 11] to conduct a narrative synthesis[17], providing an overview of all longitudinal studies that have been evaluated in this clinical cohort since 1991 (when CFS/ME was scientifically defined). There was insufficient comparable data to conduct a meta-analysis as interventions were heterogeneous and a range of outcome measures were reported. For each of the new studies, the effects of interventions on outcomes using mean differences were compared.

Patient and public involvement

No patients were involved.

Ethics approval

This study did not involve human participants.

RESULTS

Studies included

For peer review only - http://bmjopen.bmj.com/site/about/guidelines.xhtml

For peer review only

In the updated search (2015-2020), a total of 625 and 415 references were found by database searching for the depression and anxiety searches, respectively. After full-text screening, both searches returned the same two eligible studies[18, 19]. One was an RCT[19], one was a retrospective observational cohort study[18]. The PRISMA[14] flowchart is in Figure 1.

[Figure 1 here]

The previous systematic reviews for depression[10] (search conducted in 2015) and anxiety[11] (search conducted in 2016) found 362 and 1274 references, respectively. After full-text screening, the depression search returned nine eligible studies (one RCT[20], and eight observational[21-28]), and the anxiety search returned nine eligible papers from eight studies (three RCTs[29-32], six observational studies[21, 23, 24, 27, 33, 34]). Four of the studies from these two searches were the same.

Therefore, in total, 16 eligible studies were included in the narrative synthesis review. Figure 2 shows a flowchart combining studies from this updated search with studies identified from previous reviews.

[Figure 2 here]

Quality assessment

For detailed reporting on the quality assessment of studies from the previous searches, please refer to our previous two reviews[10, 11]. In this paper we report on the quality assessment of the two new studies found in the updated search.

For peer review only - http://bmjopen.bmj.com/site/about/guidelines.xhtml

The RCT[19] was conducted by members of our CFS/ME research team (EC). The study has a low risk of bias from the concealed allocation randomisation process, minimal deviation from how interventions were intended to be delivered, and appropriate intention-to-treat analysis. Outcome measurement is biased because of self-reported measures, but this is standard for behavioural treatments. It is also biased due to loss to follow-up. In the control arm at 3 months, 13 of 49 (27%) were lost to follow-up and at the primary outcome of 6 months, 12 of 49 (24%) were not included in analysis. In the intervention arm 8 of 51 (16%) were lost to follow-up at 3 months and 7 of 51 (14%) were not included in primary analysis at 6 months. Although baseline characteristics between those who did and did not provide primary outcome data were similar, it is possible that missingness was related to the outcome.

The retrospective observational study[18] is also biased due to poor follow-up rates at any one time point (making comparison difficult), and no pre-published analysis plan. In the cohort, there are two samples; one with baseline data for anxiety and depression and one without. Follow-up questionnaires were mailed to all participants on a number of occasions between January 2008 and June 2011. This produced a range of follow-up time points (1-21 years) after illness onset, meaning some patients would not have had contact with the clinic for a long time when they were sent the questionnaire, so it is likely that both disease status and time since illness influenced outcome data. Of the 489 patients who were sent baseline questionnaires, 74% returned a follow-up questionnaire on at least one occasion (range one to seven). For the sample of 366 without baseline data for anxiety and depression, 76% returned a follow-up questionnaire on one occasion, whilst only 8% returned a questionnaire on more than one occasion. Outcome measures were also self-reported, and many participants did not complete all measures.

Participant and study characteristics

For peer review only - http://bmjopen.bmj.com/site/about/guidelines.xhtml

The two studies identified in the updated search were: an RCT evaluating the 'Lightning Process' intervention alongside 'specialist medical care' compared with 'specialist medical care' alone[19]; and an observational cohort study assessing 'routine specialist care' over a 20-year period[18]. Studies from the previous reviews included the following. Four RCTs evaluating: inpatient programmes with predominantly behavioural approaches[20, 30], an online CBT programme[31, 32], and intravenous gammaglobulin[29]; eight observational cohort studies evaluating: CBT[21, 27, 34], CBT with pharmacotherapy[26, 33], an anti-viral treatment[28], and an inpatient programme[25]; and two prospective observational community studies that did not assess a specified intervention[23, 24]. Follow-up times varied from immediately post-treatment to 21 years. Total number of participants included across all studies was 965. Most sample sizes were small but ranged between one and 418. Participant ages ranged between 11 and 18. Most studies were conducted across Europe (UK, Netherlands, Spain) and Australia. One was in Japan, one in the USA (Table 2).

None of the studies identified were specifically aimed at treating anxiety or depression in children with CFS/ME (all primary outcomes were measures of fatigue or recovery). Anxiety and/or depression were measured as secondary outcomes using a variety of self-report questionnaires including the Hospital Anxiety and Depression Scale (HADS)[35], Spence Children's Anxiety Scale (SCAS)[36], the State-Trait Anxiety Inventory for Children (STAIC)[37], the Multidimensional Anxiety Scale for Children (MASC)[38], Spielberger State Trait Anxiety Questionnaire (SSTAQ)[39], Beck Depression Inventory (BDI)[40], Children's Depression Inventory[41], the Birleson Depression Scale[42], and Zung's Self-rating depression scale[43]. One study used a diagnostic interview, the Development and Well-Being Assessment (DAWBA)[44]. Six studies (including the two identified in the updated review) measured both anxiety and depression; five measured depression only; and five anxiety only (Table 2).

For peer review only - http://bmjopen.bmj.com/site/about/guidelines.xhtml

Table 2: Participant and study characteristics

Author (year), country	Anxiety, depression or both?	Study design	Setting	Sample size		Mean age, years		Gender, Female %		CFS/ME diagnostic criteria	Primary Outcome	Measure of anxiety/ depression	Treatment specifically targeted to anxiety or depression?	Outcomes stratified by those with anxiety/ depression?	Intervention	Control	Length of follow up
				Control	Intervention /case	Control	Intervention /case	Control	Intervention /case								
**(a) Studies Identified in Updated Review**																	
**Rowe et al (2019) [18], Australia**	Both	Observational retrospective	Outpatient secondary care	N/A	418 (789 recruited but 366 did not have baseline questionnaire)	N/A	14.8	N/A	77%	CDC/Fukuda	Reported recovery‡ and duration of illness	STAI, BDI	No	No	**Routine specialist medical care** provided in the outpatient clinic. Described as a person-centred goal-oriented holistic program which targets educational, physical, social and emotional aspects of life.	N/A	Mean: 8 years; Range 1- 21 years
**Crawley et al (2018)[19], UK**	Both	RCT	Outpatient secondary care	49	51	14.5	14.7	78%	75%	NICE	SF-36 PFS at 6 months	SCAS, HADS	No	No	**Specialist medical care** (Based on NICE guidance) + **Lightning Process®** (3 x 4-hour sessions on consecutive days with groups of 2-5 young people. Theory sessions teach the stress response, how the mind and body interact and how thought processes can be either helpful or negative. Practical sessions involve participants identifying a goal (e.g. stand up for longer) and are given cognitive strategies.)	Specialist medical care only	3, 6, 12 months
**(b) Studies Identified in Previous Reviews**																	
**Henderson (2014)[28], USA**	Depression	Observational, retrospective, case-series	Outpatient secondary care	N/A	15 (14 at follow-up)	N/A	15.46	N/A	73%	CDC/Fukuda	Fatigue self-assessment scores (CFSI, FSS, FSI, MFSI)	CDI	No	Yes	Valacyclovir (antiviral) medication, initially 500mg BID, increasing after 2-3 weeks. Duration of treatment ranged from 3 to 60 months (mean 27.9 months).	N/A	Varied post-treatment
**Rimes et al (2014)[34], UK**	Anxiety	Observational case-control	Outpatient secondary care	36 healthy controls	49 (24 at follow-up)	15	14.9	58%	63%	CDC/Fukuda, Oxford/ Sharpe	School attendance	SCAS	No	No	CBT via telephone based guided self-help. 6 fortnightly sessions, 30mins duration	N/A	6 months
**Nijhof et al (2012[31], 2013[32]), Netherlands**	Anxiety	RCT	Outpatient secondary care	67 (63 at follow-up)	68 (64 at follow-up)	15.8	15.9	85%	79%	CDC/Fukuda	School attendance, absence of severe fatigue and normal physical functioning	STAIC	No	No	Internet delivered CBT consisting of psychoeducation and 21 modules, with parallel child and parent sessions. FITNET therapist individually tailored intervention and initially responded to emails weekly, decreasing to fortnightly. Mean treatment duration 26.2 weeks (SD 7.3).	Treatment as usual including CBT (66%), rehabilitation treatment (22%), physical treatment (mostly graded exercise therapy; (49%), or alternative treatment (24%)	2.5 years

For peer review only - http://bmjopen.bmj.com/site/about/guidelines.xhtml

**Lloyd et al (2012)[27], UK**	Both	Observational	Outpatient secondary care	N/A	63 (52 at follow-up)	N/A	Median 15	N/A	63%	Oxford/ Sharpe	Fatigue (Chalder Fatigue Questionnaire Total) and school attendance	SCAS, Birleson Depression Scale	No	No	CBT via telephone based guided self-help. 6 fortnightly sessions, 30mins duration	N/A	6 months
**Kawatani et al (2011)[26], Japan**	Depression	Observational	Outpatient secondary care	N/A	19	N/A	13.6	N/A	63%	Jason et al [45]	Chalder's Fatigue Scale	Zung self-rating depression scale	No	No	CBT (average of 5 sessions over 6 months) and pharmacotherapy (antidepressants, antihypotensives, hypnotic agents)	N/A	6 months
**Gordon, Knapman & Lubitz (2010)[20], Australia**	Depression	RCT	Inpatient secondary care	Aerobic group: 11	Resistance group: 11	Aerobic group: 16.2	Resistance group: 15.6	Not reported		CDC/Fukuda	Exercise tolerance (time to fatigue)	BDI	No	No	4 week inpatient programme including graded exercise therapy, psychological/psychiatric support, attendance at school.<br><br>Patients randomised to either graded aerobic exercise training or progressive resistance training programme for 5 days/week for 4 weeks. The graded aerobic training consisted of 20-40 minutes of stationary cycling and treadmill exercise. The progressive resistance training involved 16 exercises performed with single set, moderate load and high repetitions.		Post-treatment
**Gordon & Lubitz (2009)[25], Australia**	Depression	Observational	Inpatient secondary care	N/A	16	N/A	16	Not reported		CDC/Fukuda	Physical and physiological measures e.g. aerobic capacity (VO_2 peak), time to fatigue, physical component score of SF-36	BDI	No	No	4 week inpatient programme including graded exercise therapy, psychological/psychiatric support, attendance at school, recreation and leisure intervention.	N/A	Post-treatment
**Diaz Caneja et al (2007)[33], Spain**	Anxiety	Observational case study	Outpatient secondary care	N/A	1	N/A	15	N/A	100%	Oxford/ Sharpe	Self-reported fatigue, pain symptoms	MASC	No	No	CBT + fluoxetine (initially 10mg daily, increased after 1 week to 20 mg)	N/A	3 months
**Rimes (2007)[23], UK**	Both	Observational prospective	Community	N/A	1 case of CFS at time 1; 4 cases of CFS at time 2	N/A	13	Not reported		CDC/Fukuda	Incidence and prevalence of fatigue, chronic fatigue and CFS	DAWBA	No	No	None specifically stated or evaluated	N/A	4-6 months
**Van de Putte et al (2007)[24], Netherlands**	Both	Observational prospective	Community	N/A	40 at baseline, 36 at follow-up	N/A	16	N/A	78%	CDC/Fukuda	Fatigue	SSTAQ, CDI	No	No	None specifically stated or evaluated	N/A	18 months

For peer review only

**Wright et al (2005)[30], UK**	Anxiety	RCT	Outpatient secondary care	6 (5 at follow-up)	7 (6 at follow-up)	12.9		66%	57%	Oxford/ Sharpe	Global Health on Child Health Questionnaire	HADS	No	No	STAIRway to Health intervention is a structured rehabilitation programme including conceptualising CFS as having both physical and psychological components, formulating and addressing vicious cycles around activity, sleep, social isolation, physical deconditioning, and developing adaptive coping strategies whilst challenging negative and unhelpful attributions about illness and the future.	Pacing - focuses on limiting activity to the changing needs and responses of the body by avoiding overexertion and managing energy within an overall limit	1 year
**Denborough et al (2003)[22], Australia**	Depression	Observational	Inpatient secondary care	N/A	39 (19 at follow-up)	N/A	16.2	N/A	90%	CDC/Fukuda	Global assessment of functioning, Chronic Fatigue Illness Disability Scale, FSS	BDI	No	No	4 week inpatient programme, focused on graded exercise using hydrotherapy and physiotherapy.	N/A	6 months
**Chalder et al (2002)[21], UK**	Both	Observational	Outpatient secondary care	N/A	23	N/A	14.5	N/A	87%	Oxford/ Sharpe	The fatigue questionnaire, school attendance	HADS	No	No	CBT based rehabilitation programme. Up to 15 sessions, 1 hour duration.	N/A	6 months
**Rowe et al (1997)[29], Australia**	Anxiety	RCT	Outpatient secondary care	35	36	15.6	15.3	75%	58%	CDC/Fukuda	Functional score including school attendance, school work, social activity and physical activity	SSTAQ	No	No	3 monthly infusions of gammaglobulin	3 monthly infusions of placebo	3 and 6 months

Note: CDC classification criteria for CFS/ME, also known as Fukuda criteria; Oxford criteria, also known as Sharpe et al criteria; SCAS, Spence Children's Anxiety Scale; HADS, Hospital Anxiety and Depression Scale; STAI(C), State-Trait Anxiety Inventory (for children); BDI, Beck's Depression Inventory; CDI, Children's Depression Inventory; MASC, Multidimensional Anxiety Scale for Children; DAWBA, Development and Well-Being Assessment; SSTAQ, Spielberger State-Trait Anxiety Questionnaire; SF-36 PFS, Short-form-36 physical function subscale; CFSI, Chronic Fatigue Syndrome Symptom Inventory; FSS, Fatigue Severity Scale; FSI, Fatigue Symptom Inventory; MFSI, Multidimensional Fatigue Symptom Inventory-Short Form; Global rating was measured on multiple scales of functioning (incl. school/work, stamina, recovery, social and symptomatology) from 1-10, with 10 being "back to normal"; † qualitative feedback included: what was useful/helpful in treatment, their perceived effectiveness, and whether anything could have been handled better; ‡reported recovery was based on the question "Do you feel you are no longer suffering from CFS?" (yes/no).

For peer review only

Treatment approaches and their efficacy treating anxiety and/or depression in paediatric CFS/ME

Of the 16 studies: one study evaluated routine specialist outpatient care[18]; one evaluated the Lightening Process outpatient intervention[19]; one evaluated the 'STAIRway to health' outpatient intervention[30]; six evaluated various outpatient CBT programmes[21, 26, 27, 31-34]; two evaluated outpatient pharmacological interventions (antivirals[28] and gammaglobulins[29]); three evaluated inpatient programmes focussed on graded exercise therapy[20, 22, 25]; and two were epidemiological observational studies so were uninformative about interventions[23, 24].

There were common cognitive and behavioural elements across the behavioural and CBT programmes, including: behavioural strategies for a goal-oriented graded approach to increasing activity, often with the goal to return to full-time education or to commit to a regular activity; cognitive strategies to address the psychological implications of CFS/ME, illness-related beliefs and negative thoughts; and psychoeducation about the consequence of the illness and tools to navigate this. They varied in their intensity (e.g. inpatient treatment, consecutive daily four-hour outpatient sessions, and fortnightly 30-minute phone calls), duration of treatment (days to years), and modality (e.g. face-to-face, telephone, and online). The antiviral and gammaglobuin studies did not include these elements and were distinct from the other studies in their approach.

Table 3 summarises outcomes of depression and/or anxiety and other relevant findings for each included study from (a) the updated review, and (b) previous reviews. Below, we discuss the efficacy of the treatment approaches in the 14 studies which evaluated an intervention, by whether they were (1) an outpatient or (2) an inpatient programme.

For peer review only - http://bmjopen.bmj.com/site/about/guidelines.xhtml

Table 3: Summary of outcomes for symptoms of depression and anxiety and other relevant findings for included studies

Study	Measure of Depression and Anxiety	Pre treatment: depression, mean(SD)		Pre treatment: anxiety, mean(SD)		Post treatment: depression, mean(SD)		Post treatment: anxiety, mean(SD)		Statistical analysis of change in depression/anxiety symptomatology		Summary of other relevant findings
		Intervention	Control	Intervention /case	Control	Intervention /case	Control	Intervention /case	Control	Depression	Anxiety	
**(a) Studies Identified in Updated Review**												
**Rowe et al (2019)[18]**	BDI* (depression scale),<br><br>STAI* (anxiety scale)	13.8 (8.9)	N/A	88.9 (24.9)	N/A	N/A	N/A	N/A	N/A	No statistical change because post-treatment scores were not measured. Instead, mean baseline depression and anxiety scores were compared between those who reported recovery‡ and those who did not, using the student's t-test.		Overall, 46.5% reported recovery; participants who were followed for >10 years, 68% reported recovery<br><br>Mean duration of illness was 5 years
**Crawley et al (2018)[19]**	HADS* (depression and anxiety scales),<br><br>SCAS* (anxiety scale)	7.5 (3.1)	8.1 (4.4)	HADS:<br>8.8 (4.5)<br><br>SCAS:<br>29.8 (16.9)	HADS:<br>10.4 (4.4)<br><br>SCAS:<br>40.3 (20.1)	6 months:<br>4.2<br><br>12 months: 2.8	6 months: 5.9<br><br>12 months: 4.6	HADS<br>6 months: 6.1<br>12 months: 5.3<br><br>SCAS<br>6 months: 24.7<br>12 months: 19.6	HADS<br>6 months: 9.7<br>12 months: 8.3<br><br>SCAS<br>6 months: 37.4<br>12 months: 36.3	Adjusted difference in means† (95%CI, pvalue):<br><br>6 months:<br>-1.5 (-3.5 to 0.5, p=0.1)<br><br>12 months:<br>-1.8 (-3.4 to -0.1, p=0.04)	Adjusted difference in means† (95%CI, pvalue):<br><br>HADS at 6 months:<br>-3.5 (-5.6 to -1.5, p=0.001)<br><br>SCAS at 6 months:<br>-10.0 (-18.5 to -1.5, p=0.02)<br><br>HADS at 12 months:<br>-2.6 (-4.7 to -0.4, p=0.019);<br><br>SCAS at 12 months:<br>14.5 (-22.4 to -6.7, p<0.001)	At 6 months, participants allocated to LP in addition to SMC (intervention) had better physical function and fatigue at than those allocated to SMC (control).<br><br>At 12 months, participants allocated to LP in addition to SMC (intervention) had better fatigue and school attendance than those in SMC (control).<br><br>Adding LP to SMC is cost-effective.
**(b) Studies Identified in Previous Reviews**												
**Henderson (2014)[28]**	CDI	14 (2.83)<br><br>4 patients with mood disorder:16.8 (1.92)<br><br>11 patients without mood disorder:<br>12.73 (2.00)	N/A	N/A	N/A	Not reported	N/A	N/A	N/A	Not reported	N/A	All patients reported at least 80% self-rated improvement. Significant reduction in FSS, MSFI (all subscales).

For peer review only

**Rimes et al (2014)[34]**	SCAS	N/A	N/A	Cases: 22 (17)<br><br>Median 16.0 (interquartile range 9.0-34.0)	Controls:<br><br>Median 16.5 (interquartile range 8.0-22.8)	N/A	N/A	Not reported	N/A	N/A	T value (21)= 2.1. p=0.005	Adolescents with CFS had reduced cortisol excretion throughout the day compared to healthy controls.<br>There was significant improvement in school attendance after treatment from 24% to 49%.<br>There was reduction in fatigue after treatment, however the results were not significant.
**Nijhof et al (2012[31], 2013[32])**	STAIC	N/A	N/A	32.7 (8.8)	32.3 (8.0)	N/A	N/A	Not reported	N/A	N/A	Not reported	Intervention (FITNET) was significantly more effective than the control (usual care) at 6 months—full school attendance (50 [75%] vs 10 [16%], relative risk 4·8, 95% CI 2·7–8·9; p<0·0001), absence of severe fatigue (57 [85%] vs 17 [27%], 3·2, 2·1–4·9; p<0·0001), and normal physical functioning (52 [78%] vs 13 [20%], 3·8, 2·3–6·3; p<0·0001). The short-term effectiveness of FITNET was maintained at 2.5 years follow-up. At 2.5 years follow-up, usual care led to similar recovery rates, although progress had taken longer to make.<br><br>At 6 months additional analyses of main findings with adjustments for anxiety, depression, and primary outcomes, had no effects on the results.<br><br>When looking at factors related to recovery at 2.5 years, anxiety OR 1.01 (95% CI 0.96-1.06), P = 0.66
**Lloyd et al (2012)[27]**	Birleson Depression Scale; SCAS	Baseline mean 13.38 (4.76)<br><br>Pre-treatment mean 12.91 (5.57)	N/A	Baseline mean 22.84 (17.18)<br><br>Baseline median 16.0 (interquartile range 10.8-35.0)	N/A	Post-treatment: 10.98 (5.35)<br><br>3 months: 10.47 (5.87)<br><br>6 months: 9.22 (5.36)	N/A	6 months: 17.25 (3.06)	N/A	Multi-level modelling and Wald tests<br>Treatment effect estimate at 6 months: 3.69 (CI -5.17, -2.21), significance (two-tailed) <0.001, effect size 0.78.	Multi-level modelling and Wald tests<br>Treatment effect estimate at 6 months: 0.49, significance (two-tailed) 0.003, effect size 0.16	Significant improvement in fatigue and school attendance, with reductions in depression and impairment and increased adjustment at 6 months
**Kawatani et al (2011)[26]**	Zung self-rating depression scale	53.3 (6.7)	N/A	N/A	N/A	Not reported	N/A	N/A	N/A	Not reported	N/A	No significant change between baseline fatigue scores and fatigue scores 6 months follow-up. Significant improvement in performance status scores (self-reported impact on functioning).

For peer review only

**Gordon, Knapman & Lubitz (2010)[20]**	BDI	Resistance arm: 20.9 (11.3)	Aerobic arm: 16.4 (4.3)	N/A	N/A	Resistance arm: 14.2 (10.0)	Aerobic arm: 12.2 (6.7)	N/A	N/A	Resistance arm Difference -6.7 +/- 8.5 p=0.03<br><br>Aerobic arm Difference -4.2 +/- 4.8 p= 0.002	N/A	There was no control group. Significant improvement in BDI scores in both arms.
**Gordon & Lubitz (2009)[25]**	BDI	19.88 (8.62)	N/A	N/A	N/A	11.44 (10.98)	N/A	N/A	N/A	Paired t test p value 0.001, sig 0.008	N/A	Significant improvement in Fatigue Severity scores.
**Diaz Caneja et al (2007)[33]**	MASC	N/A	N/A	Not stated. Raised levels of social anxiety and physical symptoms of anxiety	N/A	N/A	N/A	Not stated although it is reported that anxiety improved	N/A	N/A	Not reported	Report of a moderate response to treatment with the young person tolerating more activity. She had resumed contact with her friends, and although she still complained of tiredness and pain, she was attending classes daily.
**Rimes (2007)[23]**	DAWBA	Only states "3 of 4 had at least 1 psychiatric diagnosis at baseline"	N/A	Only states "3 of 4 had at least 1 psychiatric diagnosis at baseline"	N/A	N/A	N/A	N/A	N/A	Not reported	Not reported	Of the 4 participants who developed CFS/ME over the follow-up period, 3 of 4 had at least 1 psychiatric diagnosis at baseline, 3 had reported being 'much more tired and worn out than usual over the last month' at time 1, 2 participants had frequent headaches at time 1, 1 also had sleep problems and post-exertional malaise at time 1.
**Van de Putte et al (2007)[24]**	CDI at baseline only; HADS (anxiety)	11.7(6.1)	N/A	36.9 (7.8)	N/A	Not stated	N/A	Not stated	N/A	Not reported	Not reported	47% of adolescents 'fully recovered' (below score that is mean plus 2 SD of subjective fatigue distribution in health adolescents).
**Wright et al (2005)[30]**	HADS (anxiety)	N/A	N/A	10.17 (3.71)	6.80 (3.56)	N/A	N/A	Post-treatment: 6.00 (3.63)	Post-treatment: 6.60 (4.73)	N/A	Analysis of covariance for anxiety, controlling for baseline score. Difference -1.60 (-8.31-5.10)<br>F 0.3 (df 1,8)<br>p=0.6	Activity (child and clinician rated) and school attendance improved markedly in the intervention (STAIRway) arm compared to little improvement in activity scores in the control (Pacing) arm, and a deterioration in school attendance. Global health (child and clinician rated) improved in both arms although more in the STAIRway arm than the pacing arm.

For peer review only

For peer review only - http://bmjopen.bmj.com/site/about/guidelines.xhtml

**Denborough et al (2003)[22]**	BDI	21	N/A	N/A	N/A	15	N/A	N/A	N/A	Improvement p<0.001 Maintained at 6 month follow-up (p<0.038)	N/A	On discharge, mean depression score significantly better than on admission. Also significant improvement in Chronic Fatigue Illness Disability score and significant decrease in FSS score (maintained at 6 months follow-up). Achenbach/Youth Self-Report scores improved significantly by discharge, but returned to above admission levels at 6 months.
**Chalder et al (2002)[21]**	HADS	8.4 (interquartile range 5.7-11)	N/A	HADS anxiety: median 7,<br><br>(interquartile range 6.7-9.7)	N/A	6 months:<br>3 (interquartile range 3-5)	N/A	6 months:<br>HADS anxiety: 0.5 (IQ range 0.5-9)	N/A	Wilcoxon signed ranks test - 3.33 (2 tailed significance 0.00)	Wilcoxon signed ranks test (significance 2 tailed) HADS anxiety: 2.02 (0.04)	Depression: The 20 participants who completed treatment had all returned to school at 6 months follow-up, with 19 of 20 attending full time. Depression significantly improved, as did social adjustment.<br><br>Anxiety: All 20 treatment completers returned to school at 6 months follow-up, with 95% attending full time. Depression significantly improved, as did social adjustment.
**Rowe et al (1997)[29]**	SSTAQ	N/A	N/A	Reported as 1 group:<br>Mean 46.2 (24.4)<br>SE 3.9<br>Range 0-98		N/A	N/A	6 months:<br>Mean 28.1 (25.0)<br>SE 5.9<br>Range 0-77		N/A	T value (df) 2.63 (56)<br>Sig p value 0.01	Significant mean functional improvement in both groups.

Note: *higher score=more symptoms, poorer function; † adjusted for age, gender, baseline outcome, SCAS and visual analogue scale; ‡reported recovery was based on the question "Do you feel you are no longer suffering from CFS?" (yes/no).

HADS, Hospital Anxiety and Depression Scale (score >8 indicates a diagnosis of depression); SCAS, Spence Children's Anxiety Scale ; BDI, Beck's Depression Inventory (score >20 indicates moderate depression); STAI(C), State-Trait Anxiety Inventory (for children); BDI, Beck's Depression Inventory; CDI, Children's Depression Inventory; MASC, Multidimensional Anxiety Scale for Children; DAWBA, Development and Well-Being Assessment; SSTAQ, Spielberger State-Trait Anxiety Questionnaire; SF-36 PFS, Short-form-36 physical function subscale; CFSI, Chronic Fatigue Syndrome Symptom Inventory; FSS, Fatigue Severity Scale; FSI, Fatigue Symptom Inventory; MFSI, Multidimensional Fatigue Symptom Inventory-Short Form; LP, Lightning Process; SMC, Specialist Medical Care

For peer review only

1. Outpatient programmes

The two new studies from this updated review evaluated two outpatient programmes. Crawley et al[19] compared adding the Lightening Process intervention (https://lightningprocess.com) to specialist care (recommended by NICE[1]), to specialist medical care alone. The Lightening Process is developed from osteopathy, life coaching and neurolinguistic programming and more than 250 children use it for their CFS/ME each year in the UK[46]. It is delivered in intensive three, four-hour sessions on consecutive days in small groups, with theory elements on the stress response, how the mind and body interact and how thought processes and language can be either helpful or negative, followed by practical sessions where participants identify an activity goal and are given cognitive strategies to attempt it. The study showed a significant reduction in adjusted difference in mean depressive and anxiety symptoms at 12 months (-1.8, p=0.04 for depression; -14.5, p<0.001 for anxiety) among participants allocated to the Lightening Process intervention (in addition to specialist medical care) arm than those allocated to the specialist medical care-only control. The Lightening Process was more effective than specialist medical care at reducing anxiety symptoms compared with depression (at both 6 and 12 months follow-up). Outcomes in this study were not stratified by those with depression or anxiety, so we cannot comment on other CFS/ME outcomes (such as fatigue or recovery) in context of comorbid depression or anxiety.

The other study identified in this updated review evaluated routine specialist care delivered at the authors' CFS/ME outpatient clinic in Australia[18]. Routine specialist care offers a "person-centered goal-oriented holistic programme" to "target educational, physical, social and emotional aspects of life". This includes symptom management (e.g. sleep, migraine, dizziness, nausea, orthostatic intolerance, concentration difficulties) and focussing on increasing activity and a commitment to something enjoyable outside the home on a regular basis. This study measured depressive and anxiety symptoms at baseline but not post-treatment, so we cannot comment on the effectiveness

For peer review only - http://bmjopen.bmj.com/site/about/guidelines.xhtml

of the intervention at reducing depression or anxiety. Instead, the study compared mean baseline depression and anxiety scores between those who had self-reported 'recovery', defined as answering "yes" to the question "Do you feel you are no longer suffering from CFS?" measured at a mean length of follow-up of 8 years (range 1-21). There was no difference in depression or anxiety at baseline between those who reported that they had recovered and those who had not i.e. depression nor anxiety were found to be associated with recovery.

As per our previous reviews[10,11], several studies have evaluated other outpatient programmes. Outpatient CBT interventions demonstrated inconsistent efficacy and varied in terms of delivery modality (family-focused; face-to-face; telephone; or internet-delivered modules with therapist e-consults), intensity (15 weekly, hourly therapist-led sessions; six fortnightly 30-minute telephone calls), duration of treatment (12 weeks to one year), and whether pharmacotherapy was offered alongside CBT (anti-depressants and anti-hypotensives). Three observational studies showed that face-to-face and telephone CBT resulted in improved depression, anxiety, functioning and social adjustment[21, 27, 34]. An RCT showed that participants who received internet-based CBT demonstrated improvement in fatigue and school attendance at 6-months follow up, compared to participants who received usual care[32]. However, the study did not measure anxiety at follow-up. Two studies that evaluated CBT alongside pharmacotherapy were uninformative as they either did not reassess mood at follow-up[26], or reported on only a single case-study[33]. In terms of behavioural approaches, the STAIRway to Health – an incremental rehabilitation intervention – showed greater improvement in anxiety levels, when compared with a 'pacing' intervention in an RCT[30]. Pharmacological studies showed insufficient evidence for improving anxiety or depressive symptoms with intravenous gammaglobulin infusions or vancyclovir respectively[28, 29]

2. *Inpatient programmes*

For peer review only - http://bmjopen.bmj.com/site/about/guidelines.xhtml

As per our previous review[10], three studies[20, 22, 25] including one RCT, evidenced an improvement in mood post-treatment with a 4-week inpatient behavioural programme focused on graded exercise (including physiotherapy, aerobic exercise and resistance training), which were maintained at 6-month follow-up in one study[22]). However: they did not measure anxiety symptoms; internalising problems at 6-months returned to pre-admission levels; two studies did not have follow-up data[20, 25]; all studies had small sample sizes; and the multicomponent intervention also included psychological therapy (with no further specified details about this). Therefore, these studies are uninformative for drawing conclusions about the efficacy of this behavioural intervention, or about what the key effective components of the approach may have been.

DISCUSSION

Our updated review of interventions for comorbid depression and/or anxiety in children with CFS/ME identified only two new studies published since 2015 (one of which was conducted by members of our own research team) exposing the lack of progress in this field. One study (an RCT) showed that adding the Lightening Process intervention to specialist medical care was more effective than specialist medical care alone at reducing both depressive and, to a greater extent, anxiety symptoms. The other study (an observational cohort evaluating routine specialist care) did not measure depression or anxiety at follow-up. Combined with our results from previous reviews, we identified 16 studies of 11 different interventions for paediatric CFS/ME since 1991 that include measures of anxiety and/or depression. Of these, six did not provide follow-up measurements of anxiety and/or depression post-intervention, and none of the interventions in the studies specifically targeted comorbid anxiety and/or depression. The results of this updated review do not appreciably alter what is already known from previous reviews, that there is insufficient evidence to conclude

For peer review only - http://bmjopen.bmj.com/site/about/guidelines.xhtml

what the best interventions are for treating anxiety and/or depression in paediatric CFS/ME patients.

Strengths of the updated review include the systematic approach, the use of four reviewers, contacting authors for sub-group data, and not limiting results to English language. The limitations are the lack of eligible studies and insufficient data available for a meta-analysis. Only two papers were eligible for inclusion, of which one did not provide sufficient follow-up data to comment on the treatment efficacy of the intervention on depression and anxiety. Neither intervention was specifically designed to measure the impact on depression and anxiety and therefore studies were inadequately powered to measure this. Studies were not stratified by those who met criteria for clinical diagnoses of depression/anxiety reducing our ability to analyse effectiveness. Furthermore, neither study used diagnostic interviews for anxiety and depression, relying instead on questionnaires. Whilst HADS[47], SCAS[48], and STAI[37] questionnaires are validated for use in adolescents, only the RCADS (Revised Children's Anxiety and Depression scale), which is derived from the SCAS, has been found to have sufficient discriminative accuracy against gold standard diagnostic interviews in paediatric CFS/ME populations[5].

In conjunction with our previous reviews, we show that currently the interventions with most evidence for improvement in anxiety and depressive symptoms in CFS/ME, when compared to other interventions, such as behavioural-only or pharmacological, is CBT[10, 11]. The 'Lightening Process' programme, 'STAIRway to Health' intervention, and a 4-week multicomponent inpatient rehabilitation programme show promising results for improving anxiety and/or depressive symptoms in single RCTs, but sample sizes are small and results have not been replicated. The mechanisms for why CBT could be effective are unclear because no study targeted anxiety and depression. Further, multi-component outpatient and inpatient interventions make it difficult to

For peer review only - http://bmjopen.bmj.com/site/about/guidelines.xhtml

identify the effective element of interventions. Our updated review does not further this debate because, whilst CBT is an element of 'specialist medical care' and 'routine specialist care' interventions in the new studies, we do not know how many participants received CBT or how it was delivered. Additionally, results are not stratified by those with anxiety and/or depression. Furthermore, the differences and similarities between the Lightening Process and CBT are also unclear[49]. It should also be noted that the draft NICE guideline (expected publication date August 2021: https://www.nice.org.uk/guidance/gid-ng10091/documents/draft-guideline) does not recommend the Lightning Process for management of CFS (although this is not specifically aimed at anxiety and depression).

Other cognitive and behavioural based approaches are being trialled in CFS/ME, but are limited in contributing to our understanding of their efficacy for anxiety and depressive symptoms in CFS/ME because of a failure to include paediatric CFS/ME populations or those diagnosed with CFS/ME using recognised criteria, or measure anxiety and depressive symptoms in the 20-30%[5, 6] of children that experience them. Three studies[50-52] were excluded from our review for these reasons. For example, studies evaluating Acceptance and Commitment Therapy[50] and Mindfulness-based therapies[51] show promising results in improving the physical health, symptom burden and 'emotional distress' in children with functional somatic syndromes including CFS/ME but were excluded from this review because data for adolescent participants with CFS/ME were aggregated with those with other somatic syndromes, and the studies only measured general wellbeing outcomes rather than specifically validated anxiety and/or depression outcomes.

There is a pressing need for more work in this area to identify efficacious treatments for anxiety and depressive symptoms in paediatric CFS/ME so they can be used in clinical practice. We call upon

For peer review only - http://bmjopen.bmj.com/site/about/guidelines.xhtml

researchers to undertake paediatric CFS/ME interventions studies and use validated, diagnostic outcome measures of anxiety and depression.

CONCLUSION

This updated review highlights both the paucity of intervention studies in children with CFS/ME since 1991 and the lack of forward movement in identifying effective treatments for paediatric CFS/ME and comorbid depression and anxiety over the last five years. The overall quality of the literature remains poor and calls for paediatric CFS/ME intervention studies to target anxiety and depression, measure outcomes with validated scales, or report outcomes in subsets of patients with clinical diagnoses of anxiety and depression, have not been met. Given that comorbid anxiety and depression in paediatric CFS/ME are associated with worse outcomes, unlikely to remit spontaneously without treatment, and can be incompatible with following standard CFS/ME treatment guidance, this needs to be addressed. Future research should: improve the quality of the literature by using validated scales (as well as analyse correlation between scales) and measure anxiety and/or depression as primary outcomes in large intervention studies of comorbid anxiety and/or depression in paediatric CFS/ME.

ACKNOWLEDGEMENTS

We would like to acknowledge the support from the CFS/ME teams at the Centre for Academic Child Health at the University of Bristol and the CFS/ME service at the Royal United Hospitals Bath NHS Foundation Trust.

AUTHOR CONTRIBUTIONS

For peer review only - http://bmjopen.bmj.com/site/about/guidelines.xhtml

ML and EC conceptualised this study. PC, AR, KD, and JB performed data collection, synthesis and interpretation. PC wrote the manuscript. All authors contributed to manuscript revisions, have read the final manuscript and approved it for publication. All authors agree to be accountable for all aspects of the work.

COMPETING INTERESTS STATEMENT

Professor Crawley acts as a non-paid medical advisor for the Sussex and Kent ME society.

FUNDING STATEMENT

Dr Loades is funded by the National Institute for Health Research (NIHR Doctoral Research Fellowship, DRF-2016-09-021). This report is independent research. The views expressed in this publication are those of the authors and not necessarily those of the NHS, NIHR or the Department of Health and Social Care.

DATA STATEMENT

Not applicable.

REFERENCES

1. NICE. Chronic fatigue syndrome/myalgic encephalomyelitis (or encephalopathy): diagnosis and management (CG53). 2007 [Accessed February 2020]. Available from: https://www.nice.org.uk/guidance/CG53.

For peer review only - http://bmjopen.bmj.com/site/about/guidelines.xhtml

2. Lim E, Ahn Y, Jang E, et al. Systematic review and meta-analysis of the prevalence of chronic fatigue syndrome/myalgic encephalomyelitis (CFS/ME). *J Transl Med*, 2020;18(100) doi:10.1186/s12967-020-02269-0

3. Crawley E and Sterne JA. Association between school absence and physical function in paediatric chronic fatigue syndrome/myalgic encephalopathy. *Arch Dis Child*, 2009;94(10):752-6 doi:10.1136/adc.2008.143537

4. Falk Hvidberg M, Brinth LS, Olesen AV, et al. The Health-Related Quality of Life for Patients with Myalgic Encephalomyelitis / Chronic Fatigue Syndrome (ME/CFS). *PloS One* 2015;10(7) doi:10.1371/journal.pone.0132421

5. Loades ME, Read R, Smith L, et al. How common are depression and anxiety in adolescents with chronic fatigue syndrome (CFS) and how should we screen for these mental health co-morbidities? A clinical cohort study. *Eur Child Adolesc Psychiatry* 2020 doi:10.1007/s00787-020-01646-w

6. Loades ME, Rimes K, Ali S, et al. The presence of co-morbid mental health problems in a cohort of adolescents with chronic fatigue syndrome. *Clin Child Psychol Psychiatry* 2018;23(3): 398-408 doi:10.1177/1359104517736357

7. Sadler K, Vizard T, Ford T, et al. Mental Health of Children and Young People in England, 2017: Trends and characteristics. [Accessed February 2020]. Available from: https://digital.nhs.uk/data-and-information/publications/statistical/mental-health-of-children-and-young-people-in-england/2017/2017

8. Bould H, Collin S, Lewis G, et al. Depression in paediatric chronic fatigue syndrome. *Arch Dis Child* 2013;98(6):425-8 doi:10.1136/archdischild-2012-303396

9. Loades ME, Rimes K, Ali S, et al. Depressive symptoms in adolescents with chronic fatigue syndrome (CFS): Are rates higher than in controls and do depressive

For peer review only - http://bmjopen.bmj.com/site/about/guidelines.xhtml

symptoms affect outcome? *Clin Child Psychol Psychiatry* 2019;24(3):580-592 doi:10.1177/1359104519838

10. Loades ME, Sheils EA and Crawley E. Treatment for paediatric chronic fatigue syndrome or myalgic encephalomyelitis (CFS/ME) and comorbid depression: a systematic review. *BMJ Open* 2016;6(10) doi:10.1136/bmjopen-2016-012271

11. Stoll S, Crawley E, Richards V, et al. What treatments work for anxiety in children with chronic fatigue syndrome/myalgic encephalomyelitis (CFS/ME)? Systematic review. *BMJ open* 2017;7(9) doi:10.1136/bmjopen-2016-015481

12. Fukuda K, Straus SE, Hickie I et al., The chronic fatigue syndrome: a comprehensive approach to its definition and study. International Chronic Fatigue Syndrome Study Group. *Ann Intern Med*, 1994;121(12):953-9 10.7326/0003-4819-121-12-199412150-00009

13. Sharpe MC, Archard LC, Banatvala JE, et al. A report--chronic fatigue syndrome: guidelines for research. *J R Soc Med* 1991;84(2):118-21.

14. Moher D, Liberati A, Tetzlaff J, et al. Preferred reporting items for systematic reviews and meta-analyses: the PRISMA statement. *PLoS Med* 2009;6(7) doi:10.1136/bmj.b2535

15. Sterne J, Hernan M, Reeves B, et al. ROBINS-I: a tool for assessing risk of bias in non-randomised studies of interventions. BMJ 2016;355 doi:10.1136/bmj.i4919

16. Sterne J, Savović J, Page M, et al. RoB 2: a revised tool for assessing risk of bias in randomised trials. *BMJ* 2019;366 doi:10.1136/bmj.l4898

17. Campbell M, McKenzie J, Sowden A, et al. Synthesis without meta-analysis (SWiM) in systematic reviews: reporting guideline. *BMJ* 2020;368 doi:10.1136/bmj.l6890

For peer review only - http://bmjopen.bmj.com/site/about/guidelines.xhtml

18. Rowe K. Long Term Follow up of Young People With Chronic Fatigue Syndrome Attending a Pediatric Outpatient Service. *Frontiers in pediatrics* 2019;7(21) doi:10.3389/fped.2019.00021

19. Crawley E, Gaunt D, Garfield K, et al. Clinical and cost-effectiveness of the Lightning Process in addition to specialist medical care for paediatric chronic fatigue syndrome: randomised controlled trial. *Arch Dis Child* 2018;103(2):155-164 doi:10.1136/archdischild-2017-313375

20. Gordon BA, Knapman LM, and Lubitz L. Graduated exercise training and progressive resistance training in adolescents with chronic fatigue syndrome: a randomized controlled pilot study. *Clinical Rehabilitation* 2010;24(12):1072-1079 doi: 10.1177/0269215510371429

21. Chalder T, Tong J, and Deary V. Family cognitive behaviour therapy for chronic fatigue syndrome: an uncontrolled study. *Arch Dis Child* 2002;86(2):95-97 doi: 10.1136/adc.86.2.95

22. Denborough P, Kinsella S, Stevens J, et al. Evaluation of a Multidisciplinary Inpatient Rehabilitation Programme for Adolescents With Chronic Fatigue Syndrome. *Australas Psychiatry* 2003;11(3):319-324 doi: 10.1046/j.1440-1665.2003.00559.x

23. Rimes K, Goodman R, Hotopf M, et al. Incidence, Prognosis, and Risk Factors for Fatigue and Chronic Fatigue Syndrome in Adolescents: A Prospective Community Study. *Pediatrics* 2007;119(3):e603 doi: 10.1542/peds.2006-2231

24. van de Putte EM, Engelbert RH, Kuis W, et al. Alexithymia in adolescents with chronic fatigue syndrome. *J Psychosom Res* 2007;63(4):377-80 doi: 10.1016/j.jpsychores.2007.07.009

For peer review only

25. Gordon B and Lubitz L. Promising outcomes of an adolescent chronic fatigue syndrome inpatient programme. *J Paediatr Child Health* 2009;45(5):286-90 doi: 10.1111/j.1440-1754.2009.01493.x

26. Kawatani J, Mizuno K, Shiraishi S, et al. Cognitive dysfunction and mental fatigue in childhood chronic fatigue syndrome - a 6-month follow-up study. *Brain and Development* 2011;33(10):832-841 doi: 10.1016/j.braindev.2010.12.009

27. Lloyd S, Chalder T, Sallis H, et al. Telephone-based guided self-help for adolescents with chronic fatigue syndrome: A non-randomised cohort study. *Behaviour Research and Therapy* 2012;50(5):304-312 doi: 10.1016/j.brat.2012.02.014

28. Henderson TA. Valacyclovir treatment of chronic fatigue in adolescents. *Adv Mind Body Med* 2014;28(1):4-14

29. Rowe KS. Double-blind randomized controlled trial to assess the efficacy of intravenous gammaglobulin for the management of chronic fatigue syndrome in adolescents. *J Psychiatr Res* 1997;31(1):133-47 doi: 10.1016/s0022-3956(96)00047-7

30. Wright B, Ashby B, Beverley D, et al. A feasibility study comparing two treatment approaches for chronic fatigue syndrome in adolescents. *Arch Dis Child* 2005;90(4):369-372 doi: 10.1136/adc.2003.046649

31. Nijhof SL, Bleijenberg G, Uiterwaal CS, et al. Effectiveness of internet-based cognitive behavioural treatment for adolescents with chronic fatigue syndrome (FITNET): a randomised controlled trial. Lancet 2012;379(9824):1412-8 doi: 10.1016/s0140-6736(12)60025-7

32. Nijhof SL, Priesterbach LP, Uiterwaal CS, et al. Internet-Based Therapy for Adolescents With Chronic Fatigue Syndrome: Long-term Follow-up. *Pediatrics* 2013;131(6):e1788-e1795 doi: 10.1542/peds.2012-2007

For peer review only - http://bmjopen.bmj.com/site/about/guidelines.xhtml

33. Díaz-Caneja Greciano A, Rodríguez Sosa JT, Aguilera Albesa S, et al. Chronic fatigue syndrome in a 15-year-old girl. *Anales de Pediatria* 2007;67(1):74-77 doi: 10.1157/13108085

34. Rimes KA, Papadopoulos A, Cleare AJ, et al. Cortisol output in adolescents with chronic fatigue syndrome: Pilot study on the comparison with healthy adolescents and change after cognitive behavioural guided self-help treatment. *J Psychosom Res* 2014;77(5):409-414 doi: 10.1016/j.jpsychores.2014.08.018

35. Zigmond AS and Snaith RP. The hospital anxiety and depression scale. Acta Psychiatr Scand 1983;67(6):361-70 doi:10.1111/j.1600-0447.1983.tb09716.x

36. Spence SH, Barrett PM and Turner CM. Psychometric properties of the Spence Children's Anxiety Scale with young adolescents. J Anxiety Disord 2003;17(6):605-625 doi:10.1016/s0887-6185(02)00236-0

37. Spielberger CD. Manual for the state trait anxiety inventory for children. Palo Alto: Consulting Psychologists Press 1973.

38. March JS, Parker JD, Sullivan K, et al. The Multidimensional Anxiety Scale for Children (MASC): Factor Structure, Reliability, and Validity. *J Am Acad of Child & Adolesc Psychiatry* 1997;36(4):554-565 doi: 10.1097/00004583-199704000-00019

39. Spielberger C. Self-evaluation questionnaire state trait anxiety inventory. Palo Alto, CA: Consulting Psychologists Press. 1977

40. Beck AT, Steer RA and Carbin MG. Psychometric properties of the Beck Depression Inventory: Twenty-five years of evaluation. Clin Psychol Rev 1988;8(1):77-100 doi:10.1016/0272-7358(88)90050-5

For peer review only - http://bmjopen.bmj.com/site/about/guidelines.xhtml

41. Saylor CF, Finch A, Spirito A, et al. The children's depression inventory: a systematic evaluation of psychometric properties. *J Consult Clin Psychol* 1984;52(6):955-67 doi: 10.1037//0022-006x.52.6.955

42. Birleson P, Hudson I, Buchanan D, et al. Clinical Evaluation of a Self-rating Scale for Deprressive Disorder in Childhood (Depression Self-Rating Scale). *J Child Psychol Psychiatry* 1987;28(1):43-60 doi: 10.1111/j.1469-7610.1987.tb00651.x

43. Zung W. A self-rating depression scale. *Arch Gen Psychiatry* 1965;12:63-70 doi: 10.1001/archpsyc.1965.01720310065008

44. Goodman R, Ford T, Richards H, et al. The Development and Well-Being Assessment: description and initial validation of an integrated assessment of child and adolescent psychopathology. *J Child Psychol Psychiatry* 2000;41(5): 645-55

45. Jason LA, Jordan K, Miike T, et al. A Pediatric Case Definition for Myalgic Encephalomyelitis and Chronic Fatigue Syndrome. *Journal of Chronic Fatigue Syndrome* 2006;13(2-3):1-44 doi: 10.1300/J092v13n02_01

46. Reme SE, Archer N, and Chalder T. Experiences of young people who have undergone the Lightning Process to treat chronic fatigue syndrome/myalgic encephalomyelitis--a qualitative study. *Br J Health Psychol* 2013;18(3):508-25 doi: 10.1111/j.2044-8287.2012.02093.x

47. White D, Leach C, Sims R, et al. Validation of the Hospital Anxiety and Depression Scale for use with adolescents. Br J Psychiatry 1999;(175):452-454 doi:10.1192/bjp.175.5.452

48. Orgilés M, Fernández-Martínez I, Guillén-Riquelme A, et al. A systematic review of the factor structure and reliability of the Spence Children's Anxiety Scale. J Affect Disord 2016(190):333-340 doi:10.1016/j.jad.2015.09.055

For peer review only - http://bmjopen.bmj.com/site/about/guidelines.xhtml

49. Anderson E, Loades M, Starbuck J, et al. CBT repackaged or a novel treatment? The Lightning Process® compared with specialist medical care for paediatric Chronic Fatigue Syndrome. *Biomedicine, Health & Behavior* 2021;1-20 doi: 10.1080/21641846.2021.1935373

50. Kallesøe K, Schröder A, Wicksell R, et al. Feasibility of group-based acceptance and commitment therapy for adolescents (AHEAD) with multiple functional somatic syndromes: a pilot study. BMC Psychiatry 2020;20(1):457 doi:10.1186/s12888-020-02862-z

51. Ali A, Weiss T, Dutton A, et al. Mindfulness-Based Stress Reduction for Adolescents with Functional Somatic Syndromes: A Pilot Cohort Study. J Pediatr 2017;183:184-190 doi:10.1016/j.jpeds.2016.12.053

52. Kluck BN, Junghans-Rutelonis AN, Jones AE, et al. Adolescent Chronic Fatigue and Orthostatic Intolerance. Clinical Pediatrics 2017;56(1):85-89 doi:10.1177/0009922816644730

FIGURES AND TABLES LEGENDS

Figure 1: Flow chart for studies included in the systematic review; based on PRISMA guidelines

Figure 2: Flow chart of studies combined from updated review and previous reviews

Table 1: Inclusion criteria

Table 2: Participant and study characteristics

Table 3: Summary of outcomes for symptoms of depression and anxiety and other relevant findings for included studies

For peer review only - http://bmjopen.bmj.com/site/about/guidelines.xhtml

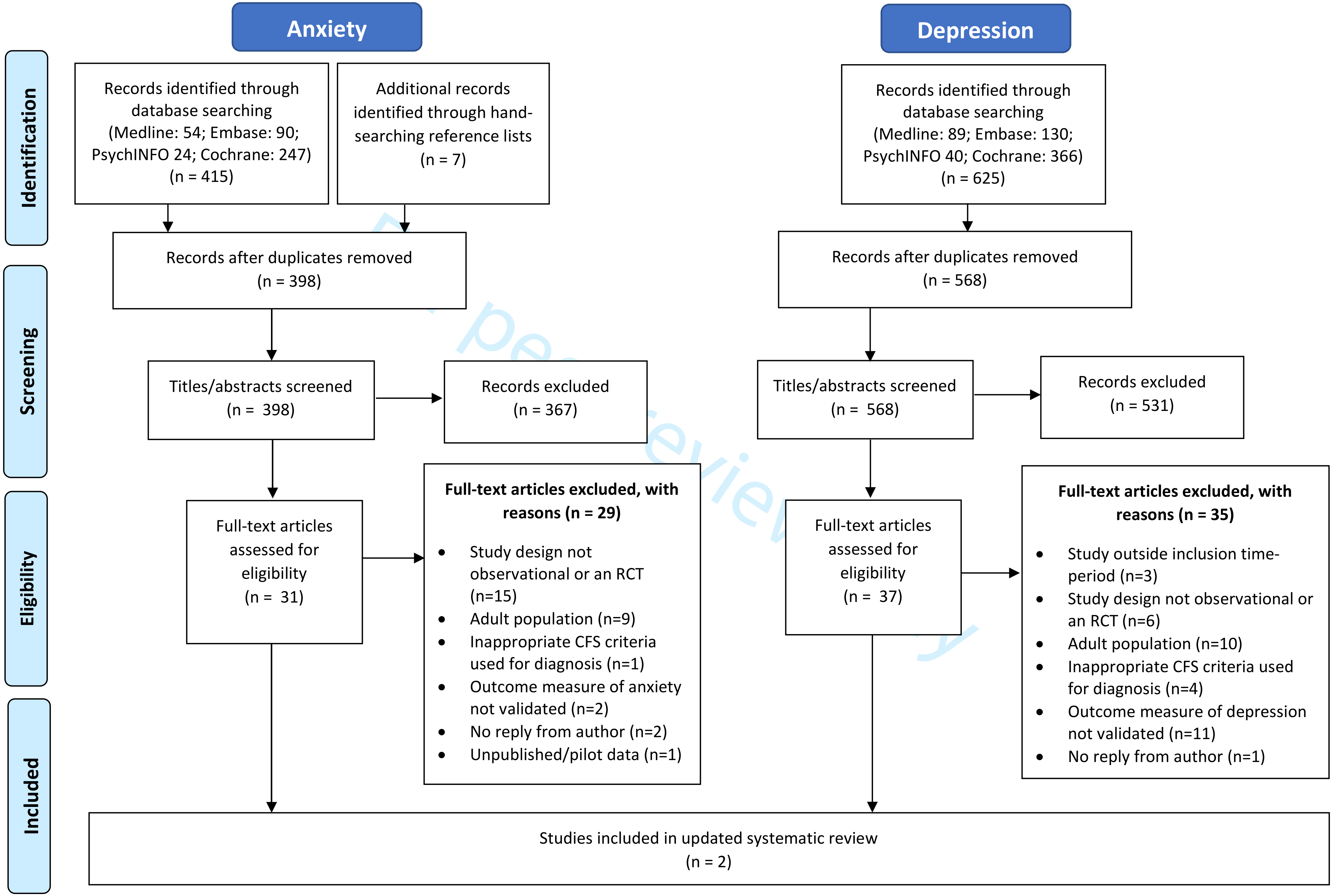

Key: CFS, chronic fatigue syndrome; ME, myalgic encephalomyelitis; PRISMA, Preferred Reporting Items for Systematic Reviews and Meta-Analyses; RCT, randomised controlled trials.

For peer review only - http://bmjopen.bmj.com/site/about/guidelines.xhtml

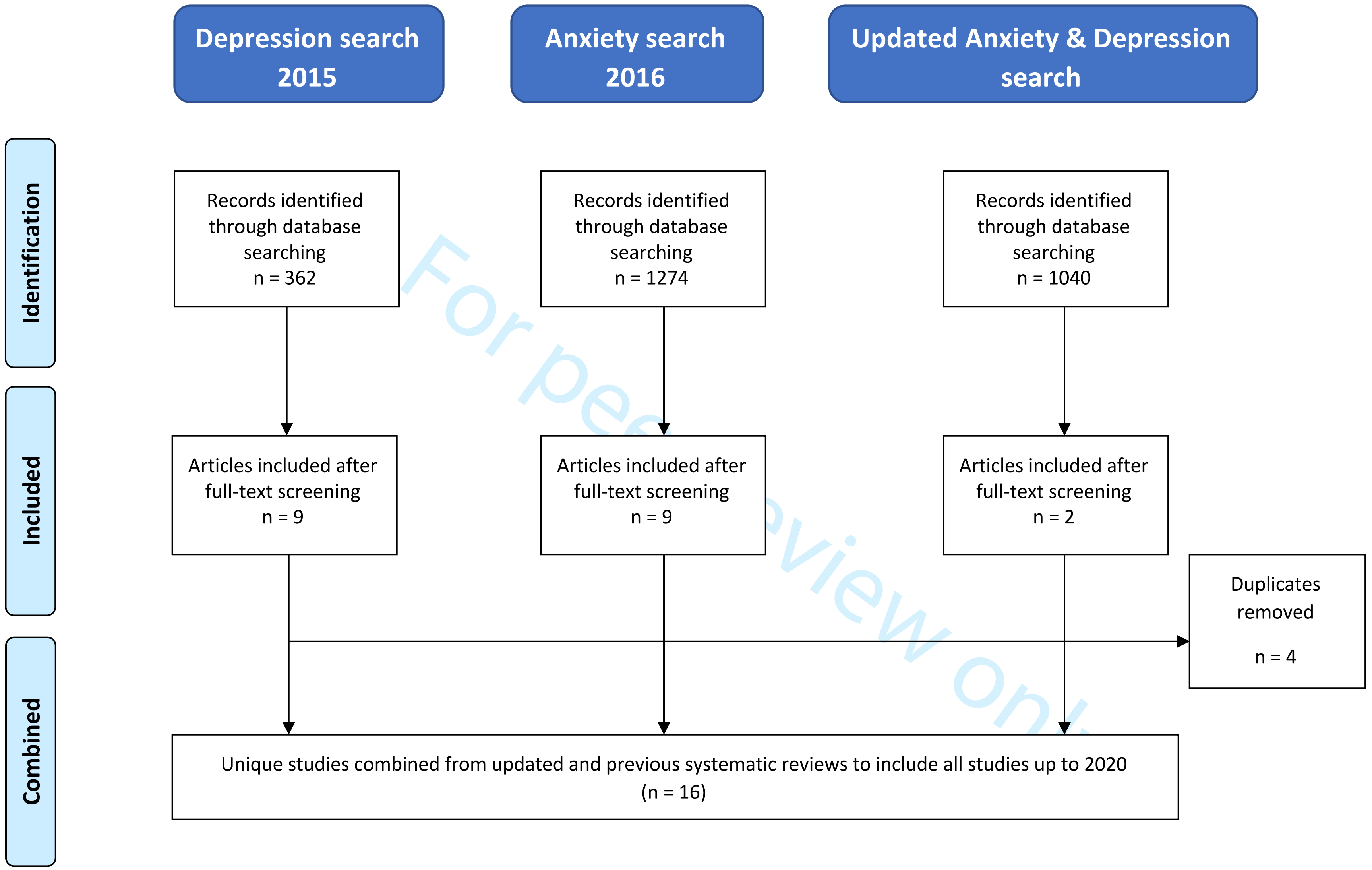
Depression search 2015
Anxiety search 2016
Updated Anxiety & Depression search
Identification
Included
Combined
Records identified through database searching n = 362
Records identified through database searching n = 1274
Records identified through database searching n = 1040
Articles included after full-text screening n = 9
Articles included after full-text screening n = 9
Articles included after full-text screening n = 2
Duplicates removed n = 4
Unique studies combined from updated and previous systematic reviews to include all studies up to 2020 (n = 16)

Search Strategies

Search strategy for Anxiety searches:

1. (adolesc* or preadolesc* or pre-adolesc* or boy* or girl* or child* or infan* or preschool* or pre-school* or juvenil* or minor* or pe?diatri* or pubescen* or pre-pubescen* or prepubescen* or puberty or teen* or young* or youth* or school* or high-school* or highschool* or sibling* or schoolchild* or school child* or children).tw.
2. exp Adolescent/ or exp Child/ or exp Child, Preschool/ or exp Infant/ or exp Minors/ or exp Pediatrics/
3. 1 or 2
4. Chronic Fatigue Syndrome.tw
5. myalgic encephal*.tw.
6. chronic fatigue syndrome*.mp.
7. myalgic encephal*.mp.
8. anxiety disorder/
9. exp anxiety disorder
10. exp obsessive-compulsive disorder
11. exp panic
12. anxi*.tw
13. generali#ed anxiety disorder.tw
14. obsessive compulsive.tw
15. OCD.tw
16. Phobia*.tw
17. Social anxiety.tw
18. Separation anxiety.tw
19. Panic.tw

For peer review only - http://bmjopen.bmj.com/site/about/guidelines.xhtml

20. exp Chronic Fatigue Syndrome/
21. exp Anxiety Disorders/ or exp Social Phobia/ or exp Panic Disorder/ or exp Anxiety/ or exp Social Anxiety
22. exp Separation Anxiety Disorder/ or Separation Anxiety/
23. exp Generalized Anxiety Disorder
24. exp Obsessive Compulsive Disorder
25. exp Phobias/
26. 4 or 5 or 6 or 7 or 20
27. 8 or 9 or 10 or 11 or 12 or 13 or 14 or 15 or 16 or 17 or 18 or 19 or 21 or 22 or 23 or 24 or 25
28. 3 and 26 and 27
29. Limit 28 to yr="2016-current"

Search strategy for Depression searches:

1. (adolesc* or preadolesc* or pre-adolesc* or boy* or girl* or child* or infan* or preschool* or pre-school* or juvenil* or minor* or pe?diatri* or pubescen* or pre-pubescen* or prepubescen* or puberty or teen* or young* or youth* or school* or high-school* or highschool* or sibling* or schoolchild* or school child* or children).tw.
2. exp Adolescent/ or exp Child/ or exp Child, Preschool/ or exp Infant/ or exp Minors/ or exp Pediatrics/
3. 1 or 2
4. chronic fatigue syndrome*.mp.
5. exp Chronic Fatigue Syndrome
6. Chronic Fatigue Syndrome.tw
7. myalgic encephal*.mp.
8. myalgic encephal*.tw.

For peer review only - http://bmjopen.bmj.com/site/about/guidelines.xhtml

9. 4 or 5 or 6 or 7 or 8
10. depressive disorder.mp.
11. exp depression/
12. depress*.tw
13. dysthymi*.tw
14. exp adjustment disorders/
15. adjustment disorder* .mp.
16. low mood.tw.
17. 10 or 11 or 12 or 14 or 14 or 15 or 16
18. 3 and 9 and 17
19. Limit 18 to yr = “2015 – current”

For peer review only

For peer review only - http://bmjopen.bmj.com/site/about/guidelines.xhtml

PRISMA 2009 Checklist

Section/topic	#	Checklist item	Reported on page #
**TITLE**			
Title	1	Identify the report as a systematic review, meta-analysis, or both.	1
**ABSTRACT**			
Structured summary	2	Provide a structured summary including, as applicable: background; objectives; data sources; study eligibility criteria, participants, and interventions; study appraisal and synthesis methods; results; limitations; conclusions and implications of key findings; systematic review registration number.	2-3
**INTRODUCTION**			
Rationale	3	Describe the rationale for the review in the context of what is already known.	4-5
Objectives	4	Provide an explicit statement of questions being addressed with reference to participants, interventions, comparisons, outcomes, and study design (PICOS).	5
**METHODS**			
Protocol and registration	5	Indicate if a review protocol exists, if and where it can be accessed (e.g., Web address), and, if available, provide registration information including registration number.	3
Eligibility criteria	6	Specify study characteristics (e.g., PICOS, length of follow-up) and report characteristics (e.g., years considered, language, publication status) used as criteria for eligibility, giving rationale.	Table 1 page 6
Information sources	7	Describe all information sources (e.g., databases with dates of coverage, contact with study authors to identify additional studies) in the search and date last searched.	5-6
Search	8	Present full electronic search strategy for at least one database, including any limits used, such that it could be repeated.	Supplementary
Study selection	9	State the process for selecting studies (i.e., screening, eligibility, included in systematic review, and, if applicable, included in the meta-analysis).	7
Data collection process	10	Describe method of data extraction from reports (e.g., piloted forms, independently, in duplicate) and any processes for obtaining and confirming data from investigators.	7
Data items	11	List and define all variables for which data were sought (e.g., PICOS, funding sources) and any assumptions and simplifications made.	6
Risk of bias in individual studies	12	Describe methods used for assessing risk of bias of individual studies (including specification of whether this was done at the study or outcome level), and how this information is to be used in any data synthesis.	8-9
Summary measures	13	State the principal summary measures (e.g., risk ratio, difference in means).	7

For peer review only

PRISMA 2009 Checklist

Synthesis of results	14	Describe the methods of handling data and combining results of studies, if done, including measures of consistency (e.g., I^2) for each meta-analysis.	N/A

Section/topic	#	Checklist item	Reported on page #
Risk of bias across studies	15	Specify any assessment of risk of bias that may affect the cumulative evidence (e.g., publication bias, selective reporting within studies).	9
Additional analyses	16	Describe methods of additional analyses (e.g., sensitivity or subgroup analyses, meta-regression), if done, indicating which were pre-specified.	N/A
**RESULTS**			
Study selection	17	Give numbers of studies screened, assessed for eligibility, and included in the review, with reasons for exclusions at each stage, ideally with a flow diagram.	8
Study characteristics	18	For each study, present characteristics for which data were extracted (e.g., study size, PICOS, follow-up period) and provide the citations.	9-10and Table 2
Risk of bias within studies	19	Present data on risk of bias of each study and, if available, any outcome level assessment (see item 12).	8-9
Results of individual studies	20	For all outcomes considered (benefits or harms), present, for each study: (a) simple summary data for each intervention group (b) effect estimates and confidence intervals, ideally with a forest plot.	Table 4
Synthesis of results	21	Present results of each meta-analysis done, including confidence intervals and measures of consistency.	N/A
Risk of bias across studies	22	Present results of any assessment of risk of bias across studies (see Item 15).	8-9
Additional analysis	23	Give results of additional analyses, if done (e.g., sensitivity or subgroup analyses, meta-regression [see Item 16]).	N/A
**DISCUSSION**			
Summary of evidence	24	Summarize the main findings including the strength of evidence for each main outcome; consider their relevance to key groups (e.g., healthcare providers, users, and policy makers).	17-18
Limitations	25	Discuss limitations at study and outcome level (e.g., risk of bias), and at review-level (e.g., incomplete retrieval of identified research, reporting bias).	17
Conclusions	26	Provide a general interpretation of the results in the context of other evidence, and implications for future research.	17-18
**FUNDING**			
Funding	27	Describe sources of funding for the systematic review and other support (e.g., supply of data); role of funders for the systematic review.	20

For peer review only

PRISMA 2009 Checklist

From: Moher D, Liberati A, Tetzlaff J, Altman DG, The PRISMA Group (2009). Preferred Reporting Items for Systematic Reviews and Meta-Analyses: The PRISMA Statement. PLoS Med 6(7): e1000097. doi:10.1371/journal.pmed1000097

For more information, visit: **www.prisma-statement.org**.

For peer review only

For peer review only - http://bmjopen.bmj.com/site/about/guidelines.xhtml

BMJ Open

What treatments work for anxiety and depression in children and adolescents with Chronic Fatigue Syndrome? An updated systematic review

Journal:	*BMJ Open*
Manuscript ID	bmjopen-2021-051358.R2
Article Type:	Original research
Date Submitted by the Author:	16-Nov-2021
Complete List of Authors:	Clery, Philippa; University of Bristol, Centre for Academic Child Health<br>Royston, Alexander; University of Bristol, Centre for Academic Child Health<br>Driver, Katie; University of Bristol, Centre for Academic Child Health<br>Bailey, Jasmine; University of Bristol, Centre for Academic Child Health<br>Crawley, Esther; University of Bristol, Centre for Academic Child Health; Royal United Hospitals Bath NHS Foundation Trust, Paediatric Chronic Fatigue Syndrome Specialist Service<br>Loades, Maria; University of Bristol, Centre for Academic Child Health; University of Bath, Department of Psychology
**Primary Subject Heading**:	Paediatrics
Secondary Subject Heading:	Paediatrics
Keywords:	PAEDIATRICS, Anxiety disorders < PSYCHIATRY, Depression & mood disorders < PSYCHIATRY, MENTAL HEALTH, Child & adolescent psychiatry < PSYCHIATRY

SCHOLARONE™
Manuscripts

I, the Submitting Author has the right to grant and does grant on behalf of all authors of the Work (as defined in the below author licence), an exclusive licence and/or a non-exclusive licence for contributions from authors who are: i) UK Crown employees; ii) where BMJ has agreed a CC-BY licence shall apply, and/or iii) in accordance with the terms applicable for US Federal Government officers or employees acting as part of their official duties; on a worldwide, perpetual, irrevocable, royalty-free basis to BMJ Publishing Group Ltd ("BMJ") its licensees and where the relevant Journal is co-owned by BMJ to the co-owners of the Journal, to publish the Work in this journal and any other BMJ products and to exploit all rights, as set out in our licence.

The Submitting Author accepts and understands that any supply made under these terms is made by BMJ to the Submitting Author unless you are acting as an employee on behalf of your employer or a postgraduate student of an affiliated institution which is paying any applicable article publishing charge ("APC") for Open Access articles. Where the Submitting Author wishes to make the Work available on an Open Access basis (and intends to pay the relevant APC), the terms of reuse of such Open Access shall be governed by a Creative Commons licence – details of these licences and which Creative Commons licence will apply to this Work are set out in our licence referred to above.

Other than as permitted in any relevant BMJ Author's Self Archiving Policies, I confirm this Work has not been accepted for publication elsewhere, is not being considered for publication elsewhere and does not duplicate material already published. I confirm all authors consent to publication of this Work and authorise the granting of this licence.

For peer review only

What treatments work for anxiety and depression in children and adolescents with Chronic Fatigue Syndrome? An updated systematic review

Philippa Clery[1], Alexander Royston[1], Katie Driver[1], Jasmine Bailey[1], Esther Crawley[1,2], Maria Loades[1,3]

[1] Centre for Academic Child Health, Bristol Medical School, Bristol, UK

[2] Paediatric Chronic Fatigue Syndrome Specialist Service, Royal United Hospitals Bath NHS Foundation Trust, Bath, UK

[3] Department of Psychology, University of Bath, Bath, UK

Corresponding author:

Dr Philippa Clery

Centre for Academic Child Health, 1-5 Whiteladies Road, Bristol, BS8 1NU, UK.

Philippa.clery@bristol.ac.uk

Word count: 3624

For peer review only - http://bmjopen.bmj.com/site/about/guidelines.xhtml

ABSTRACT

Objectives

Children with Chronic Fatigue Syndrome/Myalgic Encephalomyelitis (CFS/ME) experience a higher prevalence of depression and anxiety compared to age-matched controls. Our previous systematic reviews in 2015/16 found little evidence for effective treatment for children with CFS/ME with comorbid depression and/or anxiety. This review updates these findings.

Design

A systematic review. We searched Cochrane library, Medline, Embase and PsychINFO databases from 2015-2020. We combined the updated results with our previous reviews in a narrative synthesis.

Participants

Inclusion criteria: <18 years old; diagnosed with CFS/ME (using Centre for Disease Control, National Institute for Health and Care Excellence, or Oxford criteria); validated measures of depression and/or anxiety.

Interventions

Observational studies or randomised controlled trials.

Comparison

Any or none.

Outcomes

Studies with outcome measures of anxiety, depression, or fatigue.

Results

For peer review only

For peer review only - http://bmjopen.bmj.com/site/about/guidelines.xhtml

The updated review identified two studies. This brings the total number of paediatric CFS/ME studies with a measure of anxiety and/or depression since 1991 to 16. None of the studies specifically targeted depression, nor anxiety. One new study showed the Lightning Process (in addition to specialist care) was more effective at reducing depressive and anxiety symptoms compared to specialist care alone. Previous studies evaluated cognitive behavioural therapy (CBT); pharmacological interventions; and behavioural approaches. CBT-type interventions had most evidence for improving comorbid anxiety and/or depressive symptoms but varied in delivery and modality. Other interventions showed promise but studies were small and have not been replicated.

Conclusion

Very few paediatric CFS/ME intervention studies have been conducted. This review update does not significantly add to what is known from previous reviews. The evidence is of poor quality and insufficient to conclude which interventions are effective at treating comorbid anxiety and/or depression in paediatric CFS/ME.

Trial registration number

Reviews are registered on the Prospective Register of Systematic Review Protocols:

http://www.crd.york.ac.uk/PROSPERO/display_record.asp?ID=CRD42016043488 ;

http://www.crd.york.ac.uk/PROSPERO/display_record.asp?ID=CRD42015016813.

Key words

Paediatric, CFS/ME, chronic fatigue syndrome, anxiety, depression

ARTICLE SUMMARY

Strengths and limitations of study

For peer review only

For peer review only - http://bmjopen.bmj.com/site/about/guidelines.xhtml

- This review used a systematic approach to identify updated evidence for treatment approaches for comorbid anxiety and/or depression in paediatric CFS/ME, and combined it with previous review results to provide a comprehensive synthesis of all evidence.
- Non-English language articles were included.
- Authors were contacted and sub-group data obtained when available.
- Grey literature and unpublished material was not included.
- There was insufficient data to carry out a meta-analysis.

INTRODUCTION

Chronic fatigue syndrome (CFS)/myalgic encephalomyelitis (ME) is a common but poorly understood condition causing disabling fatigue, malaise, myalgia, sleep difficulties, and problems concentrating[1]. In children and adolescents (henceforth referred to as children), prevalence is estimated at 0.55% (95%CI 0.22-1.35) across community, primary care and hospital populations[2]. CFS/ME has long-term impacts on children's physical, cognitive, emotional and social functioning[3, 4].

Children with CFS/ME suffer from higher rates of both depression and anxiety than age-matched population samples. The prevalence estimates of comorbid depression and anxiety are 20%[5] and 29%[6], respectively, compared to 2.1% and 7.2%[7] in adolescents without CFS/ME. In those attending a specialist CFS/ME service, 61% who meet diagnostic criteria for depression also have an anxiety disorder[5]. Having comorbid depression and/or anxiety is associated with less favourable outcomes and may impact on engaging with treatment. Comorbid depression in paediatric CFS/ME is associated with greater functional disability, worse fatigue and more pain compared with those without depression[8, 9]. Low mood, anergia and anhedonia could be barriers to motivation to

For peer review only - http://bmjopen.bmj.com/site/about/guidelines.xhtml

For peer review only

engage in behavioural treatment approaches and Cognitive Behavioural Therapy-for-fatigue (CBT-f). Depressive symptoms are therefore likely to require tailored treatment[9]. The impact of anxiety on outcomes is less clear. Given that most children with CFS/ME who have anxiety also have depression[5], it is important to explore treatments for both.

Despite the high prevalence of comorbid mental health problems, there is little evidence about the effectiveness of treatments. Our two previous systematic reviews looking at depression and anxiety outcomes in existing CFS/ME intervention studies found that no specifically adapted treatments had been trialled to target depression and anxiety in paediatric CFS/ME[10, 11]. Although CBT-f and a multicomponent inpatient programme showed promise in reducing depressive[10] and anxiety[11] symptoms, there was no consistent treatment approach for children with CFS/ME and comorbid depression or anxiety. Since conducting these reviews in 2015/16, further intervention studies may have been published. It is important and timely to review the current evidence to provide an update on what treatments should be offered to this population. Further, it is important to consider anxiety and depression together given their overlap, whereas our previous reviews considered them separately.

We conducted an updated systematic review by synthesizing the evidence regarding treatments for paediatric CFS/ME and comorbid depression and anxiety since 2015. We combined these findings with results from our previous systematic reviews (1991-2015) to give an overview of all interventions evaluated since 1991 (when CFS/ME was scientifically defined). Specifically, we aimed to address the following:

1. What treatment approaches are there for depression and anxiety in children with CFS/ME?

For peer review only - http://bmjopen.bmj.com/site/about/guidelines.xhtml

2. What is known about the treatment efficacy of these approaches for treating depression and anxiety in CFS/ME? Do different approaches have different outcomes?

METHODS

Data sources and search strategy

We conducted searches on Medline, Embase, PsychINFO and Cochrane Library databases. We used the same search strategies from the previous systematic reviews (registered on Prospero: CRD42015016813; CRD42016043488) to repeat the depression and anxiety searches separately. Searches were designed with input from an information specialist to include the concepts: paediatric; CFS/ME; anxiety and depression (search strategies are in supplementary material). We updated the searches from when they had last been run (February 2015 for depression search and July 2016 for anxiety search) up until September 2020. The two searches were carried out by different reviewer teams: anxiety search (PC, AR); depression search (KD, JB). Grey literature was not searched. Reference lists of articles for full-text screening were hand-searched.

For peer review only

Inclusion and exclusion Criteria

Studies were included if they met inclusion criteria (Table 1).

Table 1: Inclusion criteria

	Anxiety Review	Depression Review
**Participants**	1. Children <18 years of age<br>2. Diagnosed with CFS/ME defined using one of these criteria:<br>CDC aka Fukuda[12]<br>NICE[1]<br>Oxford aka Sharpe[13]	

**Interventions**	Observational cohort studies<br>Any study with intervention – e.g., observational clinical cohorts, clinical trials, etc.	
**Baseline measure**	Validated assessment of anxiety	Validated assessment of depression
**Outcome measure**	**Either** an anxiety **and/or** fatigue measure on psychometrically validated assessments or validated diagnostic interviews.	**Either** a depression **and/or** fatigue measure on psychometrically validated assessments or validated diagnostic interviews.
**Language**	Non-English language papers were considered for inclusion.	

Study selection

Articles returned from database searches were inputted into Endnote and duplicates removed. Each reviewer conducted title and abstract screening independently. Full texts of potentially eligible articles were screened against specifically created eligibility checklists. The final articles for inclusion were cross-checked between all four reviewers and any conflicts discussed and resolved with input from the senior author (ML) if necessary. Where information from the paper was insufficient to determine eligibility, authors were contacted by email for additional information. If authors did not reply after two follow-up emails, the study was excluded. Figure 1 presents the PRISMA[14] flowchart.

Data extraction

For all included articles, data were extracted independently by two reviewers (PC, AR) using a purpose-designed data extraction form to collect information about: study design; setting; recruitment; participant characteristics; CFS/ME definition used for diagnosis; assessment of depression and anxiety; other outcomes; treatment and interventions provided; definition of response and treatment/intervention outcomes.

For peer review only

Quality assessment

PC and AR used Risk of Bias assessment tools[15, 16] to assess methodological quality of the included studies.

Data synthesis

We combined results from the included studies identified in the updated search with findings from the two previous systematic reviews[10, 11] to conduct a narrative synthesis[17], providing an overview of all longitudinal studies that have been evaluated in this clinical cohort since 1991 (when CFS/ME was scientifically defined). There was insufficient comparable data to conduct a meta-analysis as interventions were heterogeneous and a range of outcome measures were reported. For each of the new studies, the effects of interventions on outcomes using mean differences were compared.

Patient and public involvement

No patients were involved.

Ethics approval

This study did not involve human participants.

RESULTS

Studies included

For peer review only - http://bmjopen.bmj.com/site/about/guidelines.xhtml

In the updated search (2015-2020), a total of 625 and 415 references were found by database searching for the depression and anxiety searches, respectively. After full-text screening, both searches returned the same two eligible studies[18, 19]. One was an RCT[19], one was a retrospective observational cohort study[18]. The PRISMA[14] flowchart is in Figure 1.

[Figure 1 here]

The previous systematic reviews for depression[10] (search conducted in 2015) and anxiety[11] (search conducted in 2016) found 362 and 1274 references, respectively. After full-text screening, the depression search returned nine eligible studies (one RCT[20], and eight observational[21-28]), and the anxiety search returned nine eligible papers from eight studies (three RCTs[29-32], six observational studies[21, 23, 24, 27, 33, 34]). Four of the studies from these two searches were the same.

Therefore, in total, 16 eligible studies were included in the narrative synthesis review. Figure 2 shows a flowchart combining studies from this updated search with studies identified from previous reviews.

[Figure 2 here]

Quality assessment

Of the total 16 studies in this review, ten were observational and six were RCTs. Of the observational studies, five had an overall risk of bias as “unclear”, and five had “high” risk of bias (as defined by the Cochrane risk of bias scale, ROBINS-I[15]). Of the RCTs, all six had an overall rating of “low” risk of bias (as defined by the Cochrane risk of bias scale (ROB-2[16]). See supplementary material for the quality assessment table. For detailed reporting on the quality assessment of studies from the

For peer review only - http://bmjopen.bmj.com/site/about/guidelines.xhtml

For peer review only

previous searches, please refer to our previous two reviews[10, 11]. In this paper we report in detail on the quality assessment of the two new studies found in the updated search.

The RCT[19] was conducted by members of our CFS/ME research team (EC). The study has a low risk of bias from the concealed allocation randomisation process, minimal deviation from how interventions were intended to be delivered, and appropriate intention-to-treat analysis. Outcome measurement is biased because of self-reported measures, but this is standard for behavioural treatments. It is also biased due to loss to follow-up. In the control arm at 3 months, 13 of 49 (27%) were lost to follow-up and at the primary outcome of 6 months, 12 of 49 (24%) were not included in analysis. In the intervention arm 8 of 51 (16%) were lost to follow-up at 3 months and 7 of 51 (14%) were not included in primary analysis at 6 months. Although baseline characteristics between those who did and did not provide primary outcome data were similar, it is possible that missingness was related to the outcome.

The retrospective observational study[18] is also biased due to poor follow-up rates at any one time point (making comparison difficult), and no pre-published analysis plan. In the cohort, there are two samples; one with baseline data for anxiety and depression and one without. Follow-up questionnaires were mailed to all participants on a number of occasions between January 2008 and June 2011. This produced a range of follow-up time points (1-21 years) after illness onset, meaning some patients would not have had contact with the clinic for a long time when they were sent the questionnaire, so it is likely that both disease status and time since illness influenced outcome data. Of the 489 patients who were sent baseline questionnaires, 74% returned a follow-up questionnaire on at least one occasion (range one to seven). For the sample of 366 without baseline data for anxiety and depression, 76% returned a follow-up questionnaire on one occasion, whilst only 8%

For peer review only

For peer review only - http://bmjopen.bmj.com/site/about/guidelines.xhtml

returned a questionnaire on more than one occasion. Outcome measures were also self-reported, and many participants did not complete all measures.

Participant and study characteristics

The two studies identified in the updated search were: an RCT evaluating the 'Lightning Process' intervention alongside 'specialist medical care' compared with 'specialist medical care' alone[19]; and an observational cohort study assessing 'routine specialist care' over a 20-year period[18]. Studies from the previous reviews included the following. Four RCTs evaluating: inpatient programmes with predominantly behavioural approaches[20, 30], an online CBT programme[31, 32], and intravenous gammaglobulin[29]; eight observational cohort studies evaluating: CBT[21, 27, 34], CBT with pharmacotherapy[26, 33], an anti-viral treatment[28], and an inpatient programme[25]; and two prospective observational community studies that did not assess a specified intervention[23, 24]. Follow-up times varied from immediately post-treatment to 21 years. Total number of participants included across all studies was 965. Most sample sizes were small but ranged between one and 418. Participant ages ranged between 11 and 18. Most studies were conducted across Europe (UK, Netherlands, Spain) and Australia. One was in Japan, one in the USA (Table 2).

None of the studies identified were specifically aimed at treating anxiety or depression in children with CFS/ME (all primary outcomes were measures of fatigue or recovery). Anxiety and/or depression were measured as secondary outcomes using a variety of self-report questionnaires including the Hospital Anxiety and Depression Scale (HADS)[35], Spence Children's Anxiety Scale (SCAS)[36], the State-Trait Anxiety Inventory for Children (STAIC)[37], the Multidimensional Anxiety Scale for Children (MASC)[38], Spielberger State Trait Anxiety Questionnaire (SSTAQ)[39], Beck Depression Inventory (BDI)[40], Children's Depression Inventory[41], the Birleson Depression

For peer review only - http://bmjopen.bmj.com/site/about/guidelines.xhtml

Scale[42], and Zung's Self-rating depression scale[43]. One study used a diagnostic interview, the Development and Well-Being Assessment (DAWBA)[44]. Six studies (including the two identified in the updated review) measured both anxiety and depression; five measured depression only; and five anxiety only (Table 2).

For peer review only

Table 2: Participant and study characteristics

Author (year), country	Anxiety, depression or both?	Study design	Setting	Sample size		Mean age, years		Gender, Female %		CFS/ME diagnostic criteria	Primary Outcome	Measure of anxiety/ depression	Treatment specifically targeted to anxiety or depression?	Outcomes stratified by those with anxiety/ depression?	Intervention	Control	Length of follow up
				Control	Intervention /case	Control	Intervention /case	Control	Intervention /case								
**(a) Studies Identified in Updated Review**																	
**Rowe et al (2019) [18], Australia**	Both	Observational retrospective	Outpatient secondary care	N/A	418 (789 recruited but 366 did not have baseline questionnaire)	N/A	14.8	N/A	77%	CDC/Fukuda	Reported recovery‡ and duration of illness	STAI, BDI	No	No	**Routine specialist medical care** provided in the outpatient clinic. Described as a person-centred goal-oriented holistic program which targets educational, physical, social and emotional aspects of life.	N/A	Mean: 8 years; Range 1- 21 years
**Crawley et al (2018)[19], UK**	Both	RCT	Outpatient secondary care	49	51	14.5	14.7	78%	75%	NICE	SF-36 PFS at 6 months	SCAS, HADS	No	No	**Specialist medical care** (Based on NICE guidance) + **Lightning Process®** (3 x 4-hour sessions on consecutive days with groups of 2-5 young people. Theory sessions teach the stress response, how the mind and body interact and how thought processes can be either helpful or negative. Practical sessions involve participants identifying a goal (e.g. stand up for longer) and are given cognitive strategies.)	Specialist medical care only	3, 6, 12 months
**(b) Studies Identified in Previous Reviews**																	
**Henderson (2014)[28], USA**	Depression	Observational, retrospective, case-series	Outpatient secondary care	N/A	15 (14 at follow-up)	N/A	15.46	N/A	73%	CDC/Fukuda	Fatigue self-assessment scores (CFSI, FSS, FSI, MFSI)	CDI	No	Yes	Valacyclovir (antiviral) medication, initially 500mg BID, increasing after 2-3 weeks. Duration of treatment ranged from 3 to 60 months (mean 27.9 months).	N/A	Varied post-treatment
**Rimes et al (2014)[34], UK**	Anxiety	Observational case-control	Outpatient secondary care	36 healthy controls	49 (24 at follow-up)	15	14.9	58%	63%	CDC/Fukuda, Oxford/ Sharpe	School attendance	SCAS	No	No	CBT via telephone based guided self-help. 6 fortnightly sessions, 30mins duration	N/A	6 months
**Nijhof et al (2012[31], 2013[32]), Netherlands**	Anxiety	Both RCTs	Outpatient secondary care	67 (63 at follow-up)	68 (64 at follow-up)	15.8	15.9	85%	79%	CDC/Fukuda	School attendance, absence of severe fatigue and normal physical functioning	STAIC	No	No	Internet delivered CBT consisting of psychoeducation and 21 modules, with parallel child and parent sessions. FITNET therapist individually tailored intervention and initially responded to emails weekly, decreasing to fortnightly. Mean treatment duration 26.2 weeks (SD 7.3).	Treatment as usual including CBT (66%), rehabilitation treatment (22%), physical treatment (mostly graded exercise therapy; (49%), or alternative treatment (24%)	2.5 years

For peer review only

**Lloyd et al (2012)[27], UK**	Both	Observational	Outpatient secondary care	N/A	63 (52 at follow-up)	N/A	Median 15	N/A	63%	Oxford/ Sharpe	Fatigue (Chalder Fatigue Questionnaire Total) and school attendance	SCAS, Birleson Depression Scale	No	No	CBT via telephone based guided self-help. 6 fortnightly sessions, 30mins duration	N/A	6 months
**Kawatani et al (2011)[26], Japan**	Depression	Observational	Outpatient secondary care	N/A	19	N/A	13.6	N/A	63%	Jason et al [45]	Chalder's Fatigue Scale	Zung self-rating depression scale	No	No	CBT (average of 5 sessions over 6 months) and pharmacotherapy (antidepressants, antihypotensives, hypnotic agents)	N/A	6 months
**Gordon, Knapman & Lubitz (2010)[20], Australia**	Depression	RCT	Inpatient secondary care	Aerobic group: 11	Resistance group: 11	Aerobic group: 16.2	Resistance group: 15.6	Not reported		CDC/Fukuda	Exercise tolerance (time to fatigue)	BDI	No	No	4 week inpatient programme including graded exercise therapy, psychological/psychiatric support, attendance at school.<br><br>Patients randomised to either graded aerobic exercise training or progressive resistance training programme for 5 days/week for 4 weeks. The graded aerobic training consisted of 20-40 minutes of stationary cycling and treadmill exercise. The progressive resistance training involved 16 exercises performed with single set, moderate load and high repetitions.		Post-treatment
**Gordon & Lubitz (2009)[25], Australia**	Depression	Observational	Inpatient secondary care	N/A	16	N/A	16	Not reported		CDC/Fukuda	Physical and physiological measures e.g. aerobic capacity (VO_2 peak), time to fatigue, physical component score of SF-36	BDI	No	No	4 week inpatient programme including graded exercise therapy, psychological/psychiatric support, attendance at school, recreation and leisure intervention.	N/A	Post-treatment
**Diaz Caneja et al (2007)[33], Spain**	Anxiety	Observational case study	Outpatient secondary care	N/A	1	N/A	15	N/A	100%	Oxford/ Sharpe	Self-reported fatigue, pain symptoms	MASC	No	No	CBT + fluoxetine (initially 10mg daily, increased after 1 week to 20 mg)	N/A	3 months
**Rimes (2007)[23], UK**	Both	Observational prospective	Community	N/A	1 case of CFS at time 1; 4 cases of CFS at time 2	N/A	13	Not reported		CDC/Fukuda	Incidence and prevalence of fatigue, chronic fatigue and CFS	DAWBA	No	No	None specifically stated or evaluated	N/A	4-6 months
**Van de Putte et al (2007)[24], Netherlands**	Both	Observational prospective	Community	N/A	40 at baseline, 36 at follow-up	N/A	16	N/A	78%	CDC/Fukuda	Fatigue	SSTAQ, CDI	No	No	None specifically stated or evaluated	N/A	18 months

For peer review only

**Wright et al (2005)[30], UK**	Anxiety	RCT	Outpatient secondary care	6 (5 at follow-up)	7 (6 at follow-up)	12.9		66%	57%	Oxford/ Sharpe	Global Health on Child Health Questionnaire	HADS	No	No	STAIRway to Health intervention is a structured rehabilitation programme including conceptualising CFS as having both physical and psychological components, formulating and addressing vicious cycles around activity, sleep, social isolation, physical deconditioning, and developing adaptive coping strategies whilst challenging negative and unhelpful attributions about illness and the future.	Pacing - focuses on limiting activity to the changing needs and responses of the body by avoiding overexertion and managing energy within an overall limit	1 year
**Denborough et al (2003)[22], Australia**	Depression	Observational	Inpatient secondary care	N/A	39 (19 at follow-up)	N/A	16.2	N/A	90%	CDC/Fukuda	Global assessment of functioning, Chronic Fatigue Illness Disability Scale, FSS	BDI	No	No	4 week inpatient programme, focused on graded exercise using hydrotherapy and physiotherapy.	N/A	6 months
**Chalder et al (2002)[21], UK**	Both	Observational	Outpatient secondary care	N/A	23	N/A	14.5	N/A	87%	Oxford/ Sharpe	The fatigue questionnaire, school attendance	HADS	No	No	CBT based rehabilitation programme. Up to 15 sessions, 1 hour duration.	N/A	6 months
**Rowe et al (1997)[29], Australia**	Anxiety	RCT	Outpatient secondary care	35	36	15.6	15.3	75%	58%	CDC/Fukuda	Functional score including school attendance, school work, social activity and physical activity	SSTAQ	No	No	3 monthly infusions of gammaglobulin	3 monthly infusions of placebo	3 and 6 months

Note: CDC classification criteria for CFS/ME, also known as Fukuda criteria; Oxford criteria, also known as Sharpe et al criteria; SCAS, Spence Children's Anxiety Scale; HADS, Hospital Anxiety and Depression Scale; STAI(C), State-Trait Anxiety Inventory (for children); BDI, Beck's Depression Inventory; CDI, Children's Depression Inventory; MASC, Multidimensional Anxiety Scale for Children; DAWBA, Development and Well-Being Assessment; SSTAQ, Spielberger State-Trait Anxiety Questionnaire; SF-36 PFS, Short-form-36 physical function subscale; CFSI, Chronic Fatigue Syndrome Symptom Inventory; FSS, Fatigue Severity Scale; FSI, Fatigue Symptom Inventory; MFSI, Multidimensional Fatigue Symptom Inventory-Short Form; Global rating was measured on multiple scales of functioning (incl. school/work, stamina, recovery, social and symptomatology) from 1-10, with 10 being "back to normal"; † qualitative feedback included: what was useful/helpful in treatment, their perceived effectiveness, and whether anything could have been handled better; ‡reported recovery was based on the question "Do you feel you are no longer suffering from CFS?" (yes/no).

For peer review only

Treatment approaches and their efficacy treating anxiety and/or depression in paediatric CFS/ME

Of the 16 studies: one study evaluated routine specialist outpatient care[18]; one evaluated the Lightening Process outpatient intervention[19]; one evaluated the 'STAIRway to health' outpatient intervention[30]; six evaluated various outpatient CBT programmes[21, 26, 27, 31-34]; two evaluated outpatient pharmacological interventions (antivirals[28] and gammaglobulins[29]); three evaluated inpatient programmes focussed on graded exercise therapy[20, 22, 25]; and two were epidemiological observational studies so were uninformative about interventions[23, 24].

There were common cognitive and behavioural elements across the behavioural and CBT programmes, including: behavioural strategies for a goal-oriented graded approach to increasing activity, often with the goal to return to full-time education or to commit to a regular activity; cognitive strategies to address the psychological implications of CFS/ME, illness-related beliefs and negative thoughts; and psychoeducation about the consequence of the illness and tools to navigate this. They varied in their intensity (e.g. inpatient treatment, consecutive daily four-hour outpatient sessions, and fortnightly 30-minute phone calls), duration of treatment (days to years), and modality (e.g. face-to-face, telephone, and online). The antiviral and gammaglobuin studies did not include these elements and were distinct from the other studies in their approach.

Table 3 summarises outcomes of depression and/or anxiety and other relevant findings for each included study from (a) the updated review, and (b) previous reviews. Below, we discuss the efficacy of the treatment approaches in the 14 studies which evaluated an intervention, by whether they were (1) an outpatient or (2) an inpatient programme.

For peer review only - http://bmjopen.bmj.com/site/about/guidelines.xhtml

Table 3: Summary of outcomes for symptoms of depression and anxiety and other relevant findings for included studies

Study	Measure of Depression and Anxiety	Pre treatment: depression, mean(SD)		Pre treatment: anxiety, mean(SD)		Post treatment: depression, mean(SD)		Post treatment: anxiety, mean(SD)		Statistical analysis of change in depression/anxiety symptomatology		Summary of other relevant findings
		Intervention	Control	Intervention /case	Control	Intervention /case	Control	Intervention /case	Control	Depression	Anxiety	
**(a) Studies Identified in Updated Review**												
**Rowe et al (2019)[18]**	BDI* (depression scale),<br><br>STAI* (anxiety scale)	13.8 (8.9)	N/A	88.9 (24.9)	N/A	N/A	N/A	N/A	N/A	No statistical change because post-treatment scores were not measured. Instead, mean baseline depression and anxiety scores were compared between those who reported recovery‡ and those who did not, using the student's t-test.		Overall, 46.5% reported recovery; participants who were followed for >10 years, 68% reported recovery<br><br>Mean duration of illness was 5 years
**Crawley et al (2018)[19]**	HADS* (depression and anxiety scales),<br><br>SCAS* (anxiety scale)	7.5 (3.1)	8.1 (4.4)	HADS:<br>8.8 (4.5)<br><br>SCAS:<br>29.8 (16.9)	HADS:<br>10.4 (4.4)<br><br>SCAS:<br>40.3 (20.1)	6 months:<br>4.2<br><br>12 months: 2.8	6 months: 5.9<br><br>12 months: 4.6	HADS<br>6 months: 6.1<br>12 months: 5.3<br><br>SCAS<br>6 months: 24.7<br>12 months: 19.6	HADS<br>6 months: 9.7<br>12 months: 8.3<br><br>SCAS<br>6 months: 37.4<br>12 months: 36.3	Adjusted difference in means† (95%CI, pvalue):<br><br>6 months:<br>-1.5 (-3.5 to 0.5, p=0.1)<br><br>12 months:<br>-1.8 (-3.4 to -0.1, p=0.04)	Adjusted difference in means† (95%CI, pvalue):<br><br>HADS at 6 months:<br>-3.5 (-5.6 to -1.5, p=0.001)<br><br>SCAS at 6 months:<br>-10.0 (-18.5 to -1.5, p=0.02)<br><br>HADS at 12 months:<br>-2.6 (-4.7 to -0.4, p=0.019);<br><br>SCAS at 12 months:<br>14.5 (-22.4 to -6.7, p<0.001)	At 6 months, participants allocated to LP in addition to SMC (intervention) had better physical function and fatigue at than those allocated to SMC (control).<br><br>At 12 months, participants allocated to LP in addition to SMC (intervention) had better fatigue and school attendance than those in SMC (control).<br><br>Adding LP to SMC is cost-effective.
**(b) Studies Identified in Previous Reviews**												
**Henderson (2014)[28]**	CDI	14 (2.83)<br><br>4 patients with mood disorder:16.8 (1.92)<br><br>11 patients without mood disorder:<br>12.73 (2.00)	N/A	N/A	N/A	Not reported	N/A	N/A	N/A	Not reported	N/A	All patients reported at least 80% self-rated improvement. Significant reduction in FSS, MSFI (all subscales).

For peer review only

For peer review only - http://bmjopen.bmj.com/site/about/guidelines.xhtml

**Rimes et al (2014)[34]**	SCAS	N/A	N/A	Cases: 22 (17)<br><br>Median 16.0 (interquartile range 9.0-34.0)	Controls:<br><br>Median 16.5 (interquartile range 8.0-22.8)	N/A	N/A	Not reported	N/A	N/A	T value (21)= 2.1. p=0.005	Adolescents with CFS had reduced cortisol excretion throughout the day compared to healthy controls.<br>There was significant improvement in school attendance after treatment from 24% to 49%.<br>There was reduction in fatigue after treatment, however the results were not significant.
**Nijhof et al (2012[31], 2013[32])**	STAIC	N/A	N/A	32.7 (8.8)	32.3 (8.0)	N/A	N/A	Not reported	N/A	N/A	Not reported	Intervention (FITNET) was significantly more effective than the control (usual care) at 6 months—full school attendance (50 [75%] vs 10 [16%], relative risk 4·8, 95% CI 2·7–8·9; p<0·0001), absence of severe fatigue (57 [85%] vs 17 [27%], 3·2, 2·1–4·9; p<0·0001), and normal physical functioning (52 [78%] vs 13 [20%], 3·8, 2·3–6·3; p<0·0001). The short-term effectiveness of FITNET was maintained at 2.5 years follow-up. At 2.5 years follow-up, usual care led to similar recovery rates, although progress had taken longer to make.<br><br>At 6 months additional analyses of main findings with adjustments for anxiety, depression, and primary outcomes, had no effects on the results.<br><br>When looking at factors related to recovery at 2.5 years, anxiety OR 1.01 (95% CI 0.96-1.06), P = 0.66
**Lloyd et al (2012)[27]**	Birleson Depression Scale; SCAS	Baseline mean 13.38 (4.76)<br><br>Pre-treatment mean 12.91 (5.57)	N/A	Baseline mean 22.84 (17.18)<br><br>Baseline median 16.0 (interquartile range 10.8-35.0)	N/A	Post-treatment: 10.98 (5.35)<br><br>3 months: 10.47 (5.87)<br><br>6 months: 9.22 (5.36)	N/A	6 months: 17.25 (3.06)	N/A	Multi-level modelling and Wald tests<br>Treatment effect estimate at 6 months: 3.69 (CI -5.17, -2.21), significance (two-tailed) <0.001, effect size 0.78.	Multi-level modelling and Wald tests<br>Treatment effect estimate at 6 months: 0.49, significance (two-tailed) 0.003, effect size 0.16	Significant improvement in fatigue and school attendance, with reductions in depression and impairment and increased adjustment at 6 months
**Kawatani et al (2011)[26]**	Zung self-rating depression scale	53.3 (6.7)	N/A	N/A	N/A	Not reported	N/A	N/A	N/A	Not reported	N/A	No significant change between baseline fatigue scores and fatigue scores 6 months follow-up. Significant improvement in performance status scores (self-reported impact on functioning).

For peer review only - http://bmjopen.bmj.com/site/about/guidelines.xhtml

**Gordon, Knapman & Lubitz (2010)[20]**	BDI	Resistance arm: 20.9 (11.3)	Aerobic arm: 16.4 (4.3)	N/A	N/A	Resistance arm: 14.2 (10.0)	Aerobic arm: 12.2 (6.7)	N/A	N/A	Resistance arm<br>Difference -6.7 +/- 8.5<br>p=0.03<br><br>Aerobic arm<br>Difference -4.2 +/- 4.8<br>p= 0.002	N/A	There was no control group. Significant improvement in BDI scores in both arms.
**Gordon & Lubitz (2009)[25]**	BDI	19.88 (8.62)	N/A	N/A	N/A	11.44 (10.98)	N/A	N/A	N/A	Paired t test p value 0.001, sig 0.008	N/A	Significant improvement in Fatigue Severity scores.
**Diaz Caneja et al (2007)[33]**	MASC	N/A	N/A	Not stated. Raised levels of social anxiety and physical symptoms of anxiety	N/A	N/A	N/A	Not stated although it is reported that anxiety improved	N/A	N/A	Not reported	Report of a moderate response to treatment with the young person tolerating more activity. She had resumed contact with her friends, and although she still complained of tiredness and pain, she was attending classes daily.
**Rimes (2007)[23]**	DAWBA	Only states "3 of 4 had at least 1 psychiatric diagnosis at baseline"	N/A	Only states "3 of 4 had at least 1 psychiatric diagnosis at baseline"	N/A	N/A	N/A	N/A	N/A	Not reported	Not reported	Of the 4 participants who developed CFS/ME over the follow-up period, 3 of 4 had at least 1 psychiatric diagnosis at baseline, 3 had reported being ‘much more tired and worn out than usual over the last month’ at time 1, 2 participants had frequent headaches at time 1, 1 also had sleep problems and post-exertional malaise at time 1.
**Van de Putte et al (2007)[24]**	CDI at baseline only; HADS (anxiety)	11.7(6.1)	N/A	36.9 (7.8)	N/A	Not stated	N/A	Not stated	N/A	Not reported	Not reported	47% of adolescents ‘fully recovered’ (below score that is mean plus 2 SD of subjective fatigue distribution in health adolescents).
**Wright et al (2005)[30]**	HADS (anxiety)	N/A	N/A	10.17 (3.71)	6.80 (3.56)	N/A	N/A	Post-treatment: 6.00 (3.63)	Post-treatment: 6.60 (4.73)	N/A	Analysis of covariance for anxiety, controlling for baseline score. Difference -1.60 (-8.31-5.10)<br>F 0.3 (df 1,8)<br>p=0.6	Activity (child and clinician rated) and school attendance improved markedly in the intervention (STAIRway) arm compared to little improvement in activity scores in the control (Pacing) arm, and a deterioration in school attendance. Global health (child and clinician rated) improved in both arms although more in the STAIRway arm than the pacing arm.

For peer review only

**Denborough et al (2003)[22]**	BDI	21	N/A	N/A	N/A	15	N/A	N/A	N/A	Improvement p<0.001 Maintained at 6 month follow-up (p<0.038)	N/A	On discharge, mean depression score significantly better than on admission. Also significant improvement in Chronic Fatigue Illness Disability score and significant decrease in FSS score (maintained at 6 months follow-up). Achenbach/Youth Self-Report scores improved significantly by discharge, but returned to above admission levels at 6 months.
**Chalder et al (2002)[21]**	HADS	8.4 (interquartile range 5.7-11)	N/A	HADS anxiety: median 7,<br><br>(interquartile range 6.7-9.7)	N/A	6 months:<br>3 (interquartile range 3-5)	N/A	6 months:<br>HADS anxiety:<br>0.5 (IQ range 0.5-9)	N/A	Wilcoxon signed ranks test - 3.33 (2 tailed significance 0.00)	Wilcoxon signed ranks test (significance 2 tailed) HADS anxiety: 2.02 (0.04)	Depression: The 20 participants who completed treatment had all returned to school at 6 months follow-up, with 19 of 20 attending full time. Depression significantly improved, as did social adjustment.<br><br>Anxiety: All 20 treatment completers returned to school at 6 months follow-up, with 95% attending full time. Depression significantly improved, as did social adjustment.
**Rowe et al (1997)[29]**	SSTAQ	N/A	N/A	Reported as 1 group:<br>Mean 46.2 (24.4)<br>SE 3.9<br>Range 0-98		N/A	N/A	6 months:<br>Mean 28.1 (25.0)<br>SE 5.9<br>Range 0-77		N/A	T value (df) 2.63 (56)<br>Sig p value 0.01	Significant mean functional improvement in both groups.

Note: *higher score=more symptoms, poorer function; † adjusted for age, gender, baseline outcome, SCAS and visual analogue scale; ‡reported recovery was based on the question "Do you feel you are no longer suffering from CFS?" (yes/no).

HADS, Hospital Anxiety and Depression Scale (score >8 indicates a diagnosis of depression); SCAS, Spence Children's Anxiety Scale ; BDI, Beck's Depression Inventory (score >20 indicates moderate depression); STAI(C), State-Trait Anxiety Inventory (for children); BDI, Beck's Depression Inventory; CDI, Children's Depression Inventory; MASC, Multidimensional Anxiety Scale for Children; DAWBA, Development and Well-Being Assessment; SSTAQ, Spielberger State-Trait Anxiety Questionnaire; SF-36 PFS, Short-form-36 physical function subscale; CFSI, Chronic Fatigue Syndrome Symptom Inventory; FSS, Fatigue Severity Scale; FSI, Fatigue Symptom Inventory; MFSI, Multidimensional Fatigue Symptom Inventory-Short Form; LP, Lightning Process; SMC, Specialist Medical Care

For peer review only

1. *Outpatient programmes*

The two new studies from this updated review evaluated two outpatient programmes. Crawley et al[19] compared adding the Lightening Process intervention (https://lightningprocess.com) to specialist care (recommended by NICE[1]), to specialist medical care alone. The Lightening Process is developed from osteopathy, life coaching and neurolinguistic programming and more than 250 children use it for their CFS/ME each year in the UK[46]. It is delivered in intensive three, four-hour sessions on consecutive days in small groups, with theory elements on the stress response, how the mind and body interact and how thought processes and language can be either helpful or negative, followed by practical sessions where participants identify an activity goal and are given cognitive strategies to attempt it. The study showed a significant reduction in adjusted difference in mean depressive and anxiety symptoms at 12 months (-1.8, p=0.04 for depression; -14.5, p<0.001 for anxiety) among participants allocated to the Lightening Process intervention (in addition to specialist medical care) arm than those allocated to the specialist medical care-only control. The Lightening Process was more effective than specialist medical care at reducing anxiety symptoms compared with depression (at both 6 and 12 months follow-up). Outcomes in this study were not stratified by those with depression or anxiety, so we cannot comment on other CFS/ME outcomes (such as fatigue or recovery) in context of comorbid depression or anxiety.

The other study identified in this updated review evaluated routine specialist care delivered at the authors' CFS/ME outpatient clinic in Australia[18]. Routine specialist care offers a "person-centered goal-oriented holistic programme" to "target educational, physical, social and emotional aspects of life". This includes symptom management (e.g. sleep, migraine, dizziness, nausea, orthostatic intolerance, concentration difficulties) and focussing on increasing activity and a commitment to something enjoyable outside the home on a regular basis. This study measured depressive and anxiety symptoms at baseline but not post-treatment, so we cannot comment on the effectiveness

For peer review only - http://bmjopen.bmj.com/site/about/guidelines.xhtml

of the intervention at reducing depression or anxiety. Instead, the study compared mean baseline depression and anxiety scores between those who had self-reported 'recovery', defined as answering "yes" to the question "Do you feel you are no longer suffering from CFS?" measured at a mean length of follow-up of 8 years (range 1-21). There was no difference in depression or anxiety at baseline between those who reported that they had recovered and those who had not i.e. depression nor anxiety were found to be associated with recovery.

As per our previous reviews[10,11], several studies have evaluated other outpatient programmes. Outpatient CBT interventions demonstrated inconsistent efficacy and varied in terms of delivery modality (family-focused; face-to-face; telephone; or internet-delivered modules with therapist e-consults), intensity (15 weekly, hourly therapist-led sessions; six fortnightly 30-minute telephone calls), duration of treatment (12 weeks to one year), and whether pharmacotherapy was offered alongside CBT (anti-depressants and anti-hypotensives). Three observational studies showed that face-to-face and telephone CBT resulted in improved depression, anxiety, functioning and social adjustment[21, 27, 34]. An RCT showed that participants who received internet-based CBT demonstrated improvement in fatigue and school attendance at 6-months follow up, compared to participants who received usual care[32]. However, the study did not measure anxiety at follow-up. Two studies that evaluated CBT alongside pharmacotherapy were uninformative as they either did not reassess mood at follow-up[26], or reported on only a single case-study[33]. In terms of behavioural approaches, the STAIRway to Health – an incremental rehabilitation intervention – showed greater improvement in anxiety levels, when compared with a 'pacing' intervention in an RCT[30]. Pharmacological studies showed insufficient evidence for improving anxiety or depressive symptoms with intravenous gammaglobulin infusions or vancyclovir respectively[28, 29]

2. *Inpatient programmes*

For peer review only - http://bmjopen.bmj.com/site/about/guidelines.xhtml

As per our previous review[10], three studies[20, 22, 25] including one RCT, evidenced an improvement in mood post-treatment with a 4-week inpatient behavioural programme focused on graded exercise (including physiotherapy, aerobic exercise and resistance training), which were maintained at 6-month follow-up in one study[22]). However: they did not measure anxiety symptoms; internalising problems at 6-months returned to pre-admission levels; two studies did not have follow-up data[20, 25]; all studies had small sample sizes; and the multicomponent intervention also included psychological therapy (with no further specified details about this). Therefore, these studies are uninformative for drawing conclusions about the efficacy of this behavioural intervention, or about what the key effective components of the approach may have been.

DISCUSSION

Our updated review of interventions for comorbid depression and/or anxiety in children with CFS/ME identified only two new studies published since 2015 (one of which was conducted by members of our own research team) exposing the lack of progress in this field. One study (an RCT) showed that adding the Lightening Process intervention to specialist medical care was more effective than specialist medical care alone at reducing both depressive and, to a greater extent, anxiety symptoms. The other study (an observational cohort evaluating routine specialist care) did not measure depression or anxiety at follow-up. Combined with our results from previous reviews, we identified 16 studies of 11 different interventions for paediatric CFS/ME since 1991 that include measures of anxiety and/or depression. Of these, six did not provide follow-up measurements of anxiety and/or depression post-intervention, and none of the interventions in the studies specifically targeted comorbid anxiety and/or depression. The results of this updated review do not appreciably alter what is already known from previous reviews, that there is insufficient evidence to conclude

For peer review only - http://bmjopen.bmj.com/site/about/guidelines.xhtml

what the best interventions are for treating anxiety and/or depression in paediatric CFS/ME patients.

Strengths of the updated review include the systematic approach, the use of four reviewers, contacting authors for sub-group data, and not limiting results to English language. The limitations are the lack of eligible studies and insufficient data available for a meta-analysis. Only two papers were eligible for inclusion, of which one did not provide sufficient follow-up data to comment on the treatment efficacy of the intervention on depression and anxiety. Neither intervention was specifically designed to measure the impact on depression and anxiety and therefore studies were inadequately powered to measure this. Studies were not stratified by those who met criteria for clinical diagnoses of depression/anxiety reducing our ability to analyse effectiveness. Furthermore, neither study used diagnostic interviews for anxiety and depression, relying instead on questionnaires. Whilst HADS[47], SCAS[48], and STAI[37] questionnaires are validated for use in adolescents, only the RCADS (Revised Children's Anxiety and Depression scale), which is derived from the SCAS, has been found to have sufficient discriminative accuracy against gold standard diagnostic interviews in paediatric CFS/ME populations[5].

In conjunction with our previous reviews, we show that currently the interventions with most evidence for improvement in anxiety and depressive symptoms in CFS/ME, when compared to other interventions, such as behavioural-only or pharmacological, is CBT[10, 11]. The 'Lightening Process' programme, 'STAIRway to Health' intervention, and a 4-week multicomponent inpatient rehabilitation programme show promising results for improving anxiety and/or depressive symptoms in single RCTs, but sample sizes are small and results have not been replicated. The mechanisms for why CBT could be effective are unclear because no study targeted anxiety and depression. Further, multi-component outpatient and inpatient interventions make it difficult to

For peer review only - http://bmjopen.bmj.com/site/about/guidelines.xhtml

identify the effective element of interventions. Our updated review does not further this debate because, whilst CBT is an element of 'specialist medical care' and 'routine specialist care' interventions in the new studies, we do not know how many participants received CBT or how it was delivered. Additionally, results are not stratified by those with anxiety and/or depression. Furthermore, the differences and similarities between the Lightening Process and CBT are also unclear[49]. It should also be noted that the draft NICE guideline (expected publication date August 2021: https://www.nice.org.uk/guidance/gid-ng10091/documents/draft-guideline) does not recommend the Lightning Process for management of CFS (although this is not specifically aimed at anxiety and depression).

Other cognitive and behavioural based approaches are being trialled in CFS/ME, but are limited in contributing to our understanding of their efficacy for anxiety and depressive symptoms in CFS/ME because of a failure to include paediatric CFS/ME populations or those diagnosed with CFS/ME using recognised criteria, or measure anxiety and depressive symptoms in the 20-30%[5, 6] of children that experience them. Three studies[50-52] were excluded from our review for these reasons. For example, studies evaluating Acceptance and Commitment Therapy[50] and Mindfulness-based therapies[51] show promising results in improving the physical health, symptom burden and 'emotional distress' in children with functional somatic syndromes including CFS/ME but were excluded from this review because data for adolescent participants with CFS/ME were aggregated with those with other somatic syndromes, and the studies only measured general wellbeing outcomes rather than specifically validated anxiety and/or depression outcomes.

There is a pressing need for more work in this area to identify efficacious treatments for anxiety and depressive symptoms in paediatric CFS/ME so they can be used in clinical practice. We call upon

For peer review only - http://bmjopen.bmj.com/site/about/guidelines.xhtml

researchers to undertake paediatric CFS/ME interventions studies and use validated, diagnostic outcome measures of anxiety and depression.

CONCLUSION

This updated review highlights both the paucity of intervention studies in children with CFS/ME since 1991 and the lack of forward movement in identifying effective treatments for paediatric CFS/ME and comorbid depression and anxiety over the last five years. The overall quality of the literature remains poor and calls for paediatric CFS/ME intervention studies to target anxiety and depression, measure outcomes with validated scales, or report outcomes in subsets of patients with clinical diagnoses of anxiety and depression, have not been met. Given that comorbid anxiety and depression in paediatric CFS/ME are associated with worse outcomes, unlikely to remit spontaneously without treatment, and can be incompatible with following standard CFS/ME treatment guidance, this needs to be addressed. Future research should: improve the quality of the literature by using validated scales (as well as analyse correlation between scales) and measure anxiety and/or depression as primary outcomes in large intervention studies of comorbid anxiety and/or depression in paediatric CFS/ME.

ACKNOWLEDGEMENTS

We would like to acknowledge the support from the CFS/ME teams at the Centre for Academic Child Health at the University of Bristol and the CFS/ME service at the Royal United Hospitals Bath NHS Foundation Trust.

AUTHOR CONTRIBUTIONS

For peer review only - http://bmjopen.bmj.com/site/about/guidelines.xhtml

ML and EC conceptualised this study. PC, AR, KD, and JB performed data collection, synthesis and interpretation. PC wrote the manuscript. All authors contributed to manuscript revisions, have read the final manuscript and approved it for publication. All authors agree to be accountable for all aspects of the work.

COMPETING INTERESTS STATEMENT

Professor Crawley acts as a non-paid medical advisor for the Sussex and Kent ME society.

FUNDING STATEMENT

Dr Loades is funded by the National Institute for Health Research (NIHR Doctoral Research Fellowship, DRF-2016-09-021). This report is independent research. The views expressed in this publication are those of the authors and not necessarily those of the NHS, NIHR or the Department of Health and Social Care.

DATA STATEMENT

Not applicable.

REFERENCES

1. NICE. Chronic fatigue syndrome/myalgic encephalomyelitis (or encephalopathy): diagnosis and management (CG53). 2007 [Accessed February 2020]. Available from: https://www.nice.org.uk/guidance/CG53.

For peer review only

For peer review only - http://bmjopen.bmj.com/site/about/guidelines.xhtml

2. Lim E, Ahn Y, Jang E, et al. Systematic review and meta-analysis of the prevalence of chronic fatigue syndrome/myalgic encephalomyelitis (CFS/ME). *J Transl Med*, 2020;18(100) doi:10.1186/s12967-020-02269-0

3. Crawley E and Sterne JA. Association between school absence and physical function in paediatric chronic fatigue syndrome/myalgic encephalopathy. *Arch Dis Child*, 2009;94(10):752-6 doi:10.1136/adc.2008.143537

4. Falk Hvidberg M, Brinth LS, Olesen AV, et al. The Health-Related Quality of Life for Patients with Myalgic Encephalomyelitis / Chronic Fatigue Syndrome (ME/CFS). *PloS One* 2015;10(7) doi:10.1371/journal.pone.0132421

5. Loades ME, Read R, Smith L, et al. How common are depression and anxiety in adolescents with chronic fatigue syndrome (CFS) and how should we screen for these mental health co-morbidities? A clinical cohort study. *Eur Child Adolesc Psychiatry* 2020 doi:10.1007/s00787-020-01646-w

6. Loades ME, Rimes K, Ali S, et al. The presence of co-morbid mental health problems in a cohort of adolescents with chronic fatigue syndrome. *Clin Child Psychol Psychiatry* 2018;23(3): 398-408 doi:10.1177/1359104517736357

7. Sadler K, Vizard T, Ford T, et al. Mental Health of Children and Young People in England, 2017: Trends and characteristics. [Accessed February 2020]. Available from: https://digital.nhs.uk/data-and-information/publications/statistical/mental-health-of-children-and-young-people-in-england/2017/2017

8. Bould H, Collin S, Lewis G, et al. Depression in paediatric chronic fatigue syndrome. *Arch Dis Child* 2013;98(6):425-8 doi:10.1136/archdischild-2012-303396

9. Loades ME, Rimes K, Ali S, et al. Depressive symptoms in adolescents with chronic fatigue syndrome (CFS): Are rates higher than in controls and do depressive

For peer review only - http://bmjopen.bmj.com/site/about/guidelines.xhtml

symptoms affect outcome? *Clin Child Psychol Psychiatry* 2019;24(3):580-592 doi:10.1177/1359104519838

10. Loades ME, Sheils EA and Crawley E. Treatment for paediatric chronic fatigue syndrome or myalgic encephalomyelitis (CFS/ME) and comorbid depression: a systematic review. *BMJ Open* 2016;6(10) doi:10.1136/bmjopen-2016-012271
11. Stoll S, Crawley E, Richards V, et al. What treatments work for anxiety in children with chronic fatigue syndrome/myalgic encephalomyelitis (CFS/ME)? Systematic review. *BMJ open* 2017;7(9) doi:10.1136/bmjopen-2016-015481
12. Fukuda K, Straus SE, Hickie I et al., The chronic fatigue syndrome: a comprehensive approach to its definition and study. International Chronic Fatigue Syndrome Study Group. *Ann Intern Med*, 1994;121(12):953-9 10.7326/0003-4819-121-12-199412150-00009
13. Sharpe MC, Archard LC, Banatvala JE, et al. A report--chronic fatigue syndrome: guidelines for research. *J R Soc Med* 1991;84(2):118-21.
14. Moher D, Liberati A, Tetzlaff J, et al. Preferred reporting items for systematic reviews and meta-analyses: the PRISMA statement. *PLoS Med* 2009;6(7) doi:10.1136/bmj.b2535
15. Sterne J, Hernan M, Reeves B, et al. ROBINS-I: a tool for assessing risk of bias in non-randomised studies of interventions. BMJ 2016;355 doi:10.1136/bmj.i4919
16. Sterne J, Savović J, Page M, et al. RoB 2: a revised tool for assessing risk of bias in randomised trials. *BMJ* 2019;366 doi:10.1136/bmj.l4898
17. Campbell M, McKenzie J, Sowden A, et al. Synthesis without meta-analysis (SWiM) in systematic reviews: reporting guideline. *BMJ* 2020;368 doi:10.1136/bmj.l6890

For peer review only - http://bmjopen.bmj.com/site/about/guidelines.xhtml

18. Rowe K. Long Term Follow up of Young People With Chronic Fatigue Syndrome Attending a Pediatric Outpatient Service. *Frontiers in pediatrics* 2019;7(21) doi:10.3389/fped.2019.00021

19. Crawley E, Gaunt D, Garfield K, et al. Clinical and cost-effectiveness of the Lightning Process in addition to specialist medical care for paediatric chronic fatigue syndrome: randomised controlled trial. *Arch Dis Child* 2018;103(2):155-164 doi:10.1136/archdischild-2017-313375

20. Gordon BA, Knapman LM, and Lubitz L. Graduated exercise training and progressive resistance training in adolescents with chronic fatigue syndrome: a randomized controlled pilot study. *Clinical Rehabilitation* 2010;24(12):1072-1079 doi: 10.1177/0269215510371429

21. Chalder T, Tong J, and Deary V. Family cognitive behaviour therapy for chronic fatigue syndrome: an uncontrolled study. *Arch Dis Child* 2002;86(2):95-97 doi: 10.1136/adc.86.2.95

22. Denborough P, Kinsella S, Stevens J, et al. Evaluation of a Multidisciplinary Inpatient Rehabilitation Programme for Adolescents With Chronic Fatigue Syndrome. *Australas Psychiatry* 2003;11(3):319-324 doi: 10.1046/j.1440-1665.2003.00559.x

23. Rimes K, Goodman R, Hotopf M, et al. Incidence, Prognosis, and Risk Factors for Fatigue and Chronic Fatigue Syndrome in Adolescents: A Prospective Community Study. *Pediatrics* 2007;119(3):e603 doi: 10.1542/peds.2006-2231

24. van de Putte EM, Engelbert RH, Kuis W, et al. Alexithymia in adolescents with chronic fatigue syndrome. *J Psychosom Res* 2007;63(4):377-80 doi: 10.1016/j.jpsychores.2007.07.009

For peer review only - http://bmjopen.bmj.com/site/about/guidelines.xhtml

25. Gordon B and Lubitz L. Promising outcomes of an adolescent chronic fatigue syndrome inpatient programme. *J Paediatr Child Health* 2009;45(5):286-90 doi: 10.1111/j.1440-1754.2009.01493.x

26. Kawatani J, Mizuno K, Shiraishi S, et al. Cognitive dysfunction and mental fatigue in childhood chronic fatigue syndrome - a 6-month follow-up study. *Brain and Development* 2011;33(10):832-841 doi: 10.1016/j.braindev.2010.12.009

27. Lloyd S, Chalder T, Sallis H, et al. Telephone-based guided self-help for adolescents with chronic fatigue syndrome: A non-randomised cohort study. *Behaviour Research and Therapy* 2012;50(5):304-312 doi: 10.1016/j.brat.2012.02.014

28. Henderson TA. Valacyclovir treatment of chronic fatigue in adolescents. *Adv Mind Body Med* 2014;28(1):4-14

29. Rowe KS. Double-blind randomized controlled trial to assess the efficacy of intravenous gammaglobulin for the management of chronic fatigue syndrome in adolescents. *J Psychiatr Res* 1997;31(1):133-47 doi: 10.1016/s0022-3956(96)00047-7

30. Wright B, Ashby B, Beverley D, et al. A feasibility study comparing two treatment approaches for chronic fatigue syndrome in adolescents. *Arch Dis Child* 2005;90(4):369-372 doi: 10.1136/adc.2003.046649

31. Nijhof SL, Bleijenberg G, Uiterwaal CS, et al. Effectiveness of internet-based cognitive behavioural treatment for adolescents with chronic fatigue syndrome (FITNET): a randomised controlled trial. Lancet 2012;379(9824):1412-8 doi: 10.1016/s0140-6736(12)60025-7

32. Nijhof SL, Priesterbach LP, Uiterwaal CS, et al. Internet-Based Therapy for Adolescents With Chronic Fatigue Syndrome: Long-term Follow-up. *Pediatrics* 2013;131(6):e1788-e1795 doi: 10.1542/peds.2012-2007

For peer review only - http://bmjopen.bmj.com/site/about/guidelines.xhtml

33. Díaz-Caneja Greciano A, Rodríguez Sosa JT, Aguilera Albesa S, et al. Chronic fatigue syndrome in a 15-year-old girl. *Anales de Pediatria* 2007;67(1):74-77 doi: 10.1157/13108085

34. Rimes KA, Papadopoulos A, Cleare AJ, et al. Cortisol output in adolescents with chronic fatigue syndrome: Pilot study on the comparison with healthy adolescents and change after cognitive behavioural guided self-help treatment. *J Psychosom Res* 2014;77(5):409-414 doi: 10.1016/j.jpsychores.2014.08.018

35. Zigmond AS and Snaith RP. The hospital anxiety and depression scale. Acta Psychiatr Scand 1983;67(6):361-70 doi:10.1111/j.1600-0447.1983.tb09716.x

36. Spence SH, Barrett PM and Turner CM. Psychometric properties of the Spence Children's Anxiety Scale with young adolescents. J Anxiety Disord 2003;17(6):605-625 doi:10.1016/s0887-6185(02)00236-0

37. Spielberger CD. Manual for the state trait anxiety inventory for children. Palo Alto: Consulting Psychologists Press 1973.

38. March JS, Parker JD, Sullivan K, et al. The Multidimensional Anxiety Scale for Children (MASC): Factor Structure, Reliability, and Validity. *J Am Acad of Child & Adolesc Psychiatry* 1997;36(4):554-565 doi: 10.1097/00004583-199704000-00019

39. Spielberger C. Self-evaluation questionnaire state trait anxiety inventory. Palo Alto, CA: Consulting Psychologists Press. 1977

40. Beck AT, Steer RA and Carbin MG. Psychometric properties of the Beck Depression Inventory: Twenty-five years of evaluation. Clin Psychol Rev 1988;8(1):77-100 doi:10.1016/0272-7358(88)90050-5

For peer review only - http://bmjopen.bmj.com/site/about/guidelines.xhtml

41. Saylor CF, Finch A, Spirito A, et al. The children's depression inventory: a systematic evaluation of psychometric properties. *J Consult Clin Psychol* 1984;52(6):955-67 doi: 10.1037//0022-006x.52.6.955

42. Birleson P, Hudson I, Buchanan D, et al. Clinical Evaluation of a Self-rating Scale for Deprressive Disorder in Childhood (Depression Self-Rating Scale). *J Child Psychol Psychiatry* 1987;28(1):43-60 doi: 10.1111/j.1469-7610.1987.tb00651.x

43. Zung W. A self-rating depression scale. *Arch Gen Psychiatry* 1965;12:63-70 doi: 10.1001/archpsyc.1965.01720310065008

44. Goodman R, Ford T, Richards H, et al. The Development and Well-Being Assessment: description and initial validation of an integrated assessment of child and adolescent psychopathology. *J Child Psychol Psychiatry* 2000;41(5): 645-55

45. Jason LA, Jordan K, Miike T, et al. A Pediatric Case Definition for Myalgic Encephalomyelitis and Chronic Fatigue Syndrome. *Journal of Chronic Fatigue Syndrome* 2006;13(2-3):1-44 doi: 10.1300/J092v13n02_01

46. Reme SE, Archer N, and Chalder T. Experiences of young people who have undergone the Lightning Process to treat chronic fatigue syndrome/myalgic encephalomyelitis--a qualitative study. *Br J Health Psychol* 2013;18(3):508-25 doi: 10.1111/j.2044-8287.2012.02093.x

47. White D, Leach C, Sims R, et al. Validation of the Hospital Anxiety and Depression Scale for use with adolescents. Br J Psychiatry 1999;(175):452-454 doi:10.1192/bjp.175.5.452

48. Orgilés M, Fernández-Martínez I, Guillén-Riquelme A, et al. A systematic review of the factor structure and reliability of the Spence Children's Anxiety Scale. J Affect Disord 2016(190):333-340 doi:10.1016/j.jad.2015.09.055

For peer review only - http://bmjopen.bmj.com/site/about/guidelines.xhtml

49. Anderson E, Loades M, Starbuck J, et al. CBT repackaged or a novel treatment? The Lightning Process® compared with specialist medical care for paediatric Chronic Fatigue Syndrome. *Biomedicine, Health & Behavior* 2021;1-20 doi: 10.1080/21641846.2021.1935373

50. Kallesøe K, Schröder A, Wicksell R, et al. Feasibility of group-based acceptance and commitment therapy for adolescents (AHEAD) with multiple functional somatic syndromes: a pilot study. BMC Psychiatry 2020;20(1):457 doi:10.1186/s12888-020-02862-z

51. Ali A, Weiss T, Dutton A, et al. Mindfulness-Based Stress Reduction for Adolescents with Functional Somatic Syndromes: A Pilot Cohort Study. J Pediatr 2017;183:184-190 doi:10.1016/j.jpeds.2016.12.053

52. Kluck BN, Junghans-Rutelonis AN, Jones AE, et al. Adolescent Chronic Fatigue and Orthostatic Intolerance. Clinical Pediatrics 2017;56(1):85-89 doi:10.1177/0009922816644730

FIGURES AND TABLES LEGENDS

Figure 1: Flow chart for studies included in the systematic review; based on PRISMA guidelines

Figure 2: Flow chart of studies combined from updated review and previous reviews

Table 1: Inclusion criteria

Table 2: Participant and study characteristics

Table 3: Summary of outcomes for symptoms of depression and anxiety and other relevant findings for included studies

For peer review only - http://bmjopen.bmj.com/site/about/guidelines.xhtml

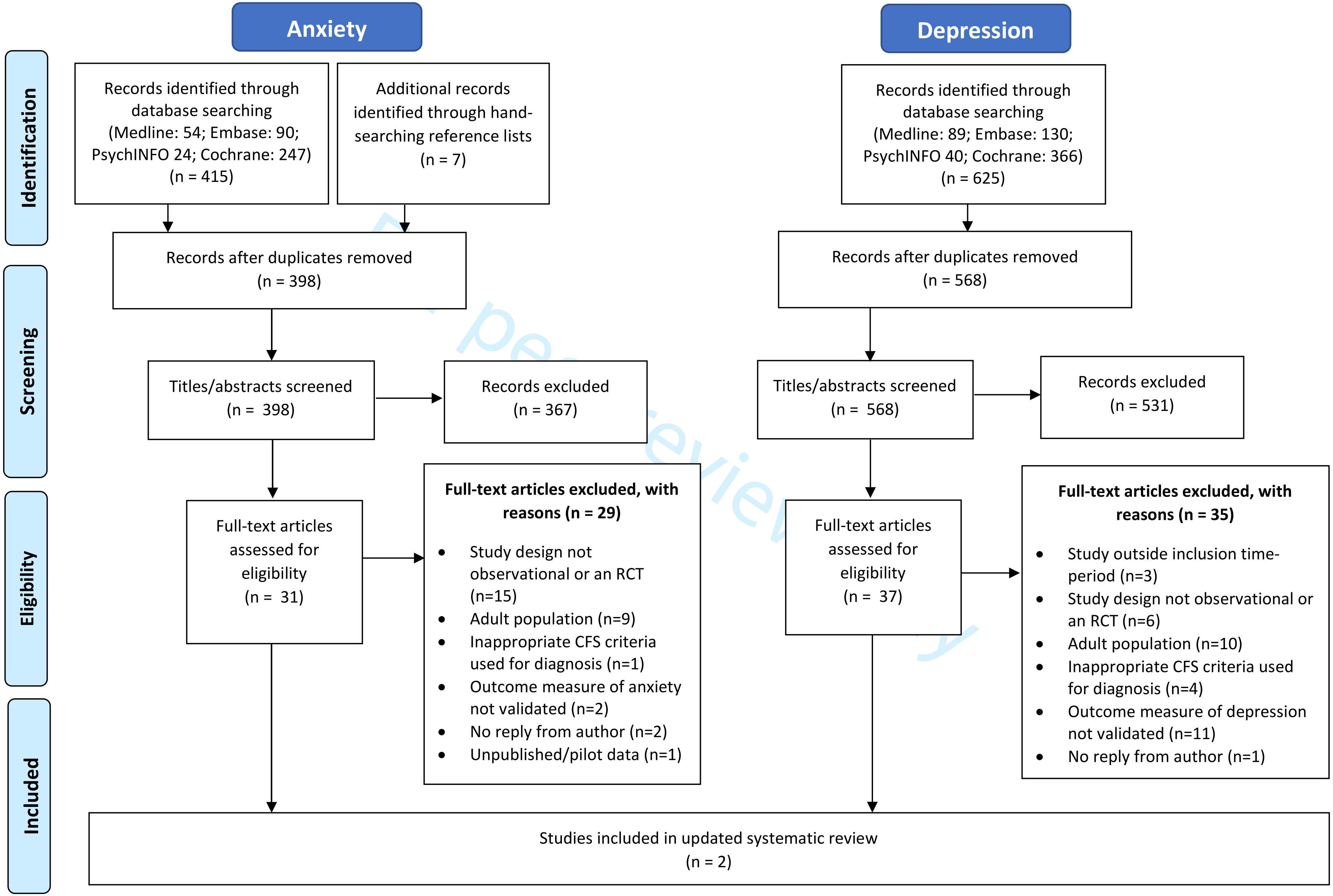

Key: CFS, chronic fatigue syndrome; ME, myalgic encephalomyelitis; PRISMA, Preferred Reporting Items for Systematic Reviews and Meta-Analyses; RCT, randomised controlled trials.

For peer review only - http://bmjopen.bmj.com/site/about/guidelines.xhtml

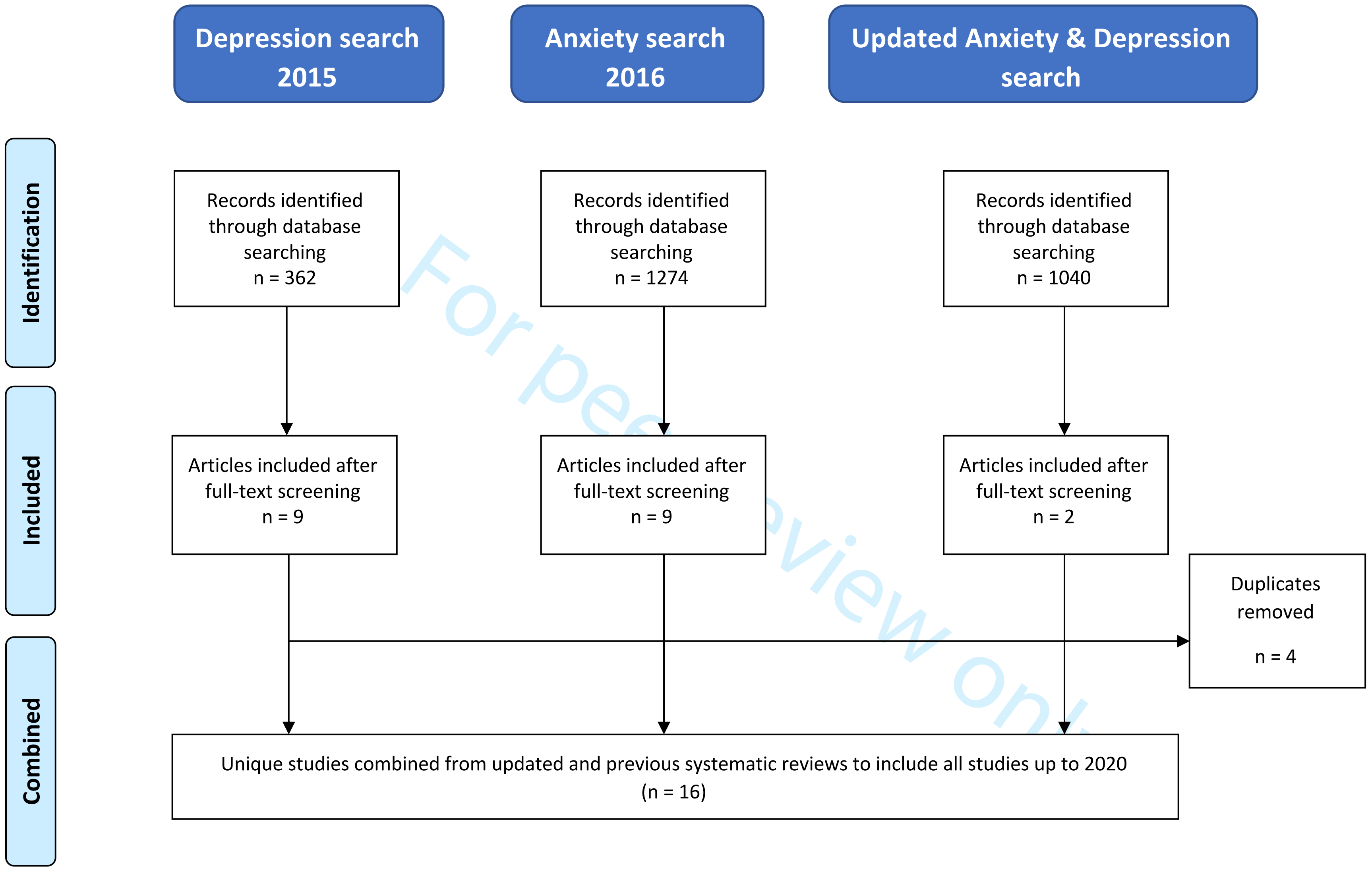
Depression search 2015
Anxiety search 2016
Updated Anxiety & Depression search
Identification
Included
Combined
Records identified through database searching n = 362
Records identified through database searching n = 1274
Records identified through database searching n = 1040
Articles included after full-text screening n = 9
Articles included after full-text screening n = 9
Articles included after full-text screening n = 2
Duplicates removed n = 4
Unique studies combined from updated and previous systematic reviews to include all studies up to 2020 (n = 16)

Supplementary Material

Appendix 1: Search Strategies

Search strategy for Anxiety searches:

1. (adolesc* or preadolesc* or pre-adolesc* or boy* or girl* or child* or infan* or preschool* or pre-school* or juvenil* or minor* or pe?diatri* or pubescen* or pre-pubescen* or prepubescen* or puberty or teen* or young* or youth* or school* or high-school* or highschool* or sibling* or schoolchild* or school child* or children).tw.
2. exp Adolescent/ or exp Child/ or exp Child, Preschool/ or exp Infant/ or exp Minors/ or exp Pediatrics/
3. 1 or 2
4. Chronic Fatigue Syndrome.tw
5. myalgic encephal*.tw.
6. chronic fatigue syndrome*.mp.
7. myalgic encephal*.mp.
8. anxiety disorder/
9. exp anxiety disorder
10. exp obsessive-compulsive disorder
11. exp panic
12. anxi*.tw
13. generali#ed anxiety disorder.tw
14. obsessive compulsive.tw
15. OCD.tw
16. Phobia*.tw
17. Social anxiety.tw
18. Separation anxiety.tw

For peer review only

For peer review only - http://bmjopen.bmj.com/site/about/guidelines.xhtml

19. Panic.tw
20. exp Chronic Fatigue Syndrome/
21. exp Anxiety Disorders/ or exp Social Phobia/ or exp Panic Disorder/ or exp Anxiety/ or exp Social Anxiety
22. exp Separation Anxiety Disorder/ or Separation Anxiety/
23. exp Generalized Anxiety Disorder
24. exp Obsessive Compulsive Disorder
25. exp Phobias/
26. 4 or 5 or 6 or 7 or 20
27. 8 or 9 or 10 or 11 or 12 or 13 or 14 or 15 or 16 or 17 or 18 or 19 or 21 or 22 or 23 or 24 or 25
28. 3 and 26 and 27
29. Limit 28 to yr="2016-current"

Search strategy for Depression searches:

1. (adolesc* or preadolesc* or pre-adolesc* or boy* or girl* or child* or infan* or preschool* or pre-school* or juvenil* or minor* or pe?diatri* or pubescen* or pre-pubescen* or prepubescen* or puberty or teen* or young* or youth* or school* or high-school* or highschool* or sibling* or schoolchild* or school child* or children).tw.
2. exp Adolescent/ or exp Child/ or exp Child, Preschool/ or exp Infant/ or exp Minors/ or exp Pediatrics/
3. 1 or 2
4. chronic fatigue syndrome*.mp.
5. exp Chronic Fatigue Syndrome
6. Chronic Fatigue Syndrome.tw
7. myalgic encephal*.mp.

For peer review only

For peer review only - http://bmjopen.bmj.com/site/about/guidelines.xhtml

8. myalgic encephal*.tw.

9. 4 or 5 or 6 or 7 or 8

10. depressive disorder.mp.

11. exp depression/

12. depress*.tw

13. dysthymi*.tw

14. exp adjustment disorders/

15. adjustment disorder* .mp.

16. low mood.tw.

17. 10 or 11 or 12 or 14 or 14 or 15 or 16

18. 3 and 9 and 17

19. Limit 18 to yr = “2015 – current

For peer review only - http://bmjopen.bmj.com/site/about/guidelines.xhtml

Appendix 2: Quality Assessment

Supplementary Table 1: Quality Assessment of all studies included in this updated review, using Cochrane Risk of Bias scales ROBINS-I and RoB-2

(a) Observational Studies

Authors (year)	Did the study address a clearly focused issue? Was this the outcome of interest to this review?	Was the cohort recruited in an acceptable way?	Was the exposure accurately measured to minimise bias?	Was the outcome accurately measured to minimise bias?	Confounding factors?	Follow-up of subjects complete enough and long enough?	Overall Rating using Cochrane risk of bias scale ROBINS-I (low/unclear/high)
**Chalder et al (2002)**	Yes, No.	Yes	Yes	Yes	Can't tell	Can't tell, yes	Unclear
**Diaz-Caneja et al (2007)**	Can't tell, No	Can't tell	Can't tell	Can't tell	Yes	Yes, no	High
**Lloyd et al (2012); Rimes et al (2014)**	Yes, No	Yes	Yes	Yes	Can't tell	Can't tell, yes	Unclear
**Rimes et al (2007)**	Yes, No	Yes	Yes	Yes	Can't tell	Can't tell, yes	Unclear
**Van de Putte et al (2007)**	Yes, No	Yes	Yes	Yes	Can't tell	Can't tell, yes	Unclear
**Kawatani et al (2011)**	Yes, No	Yes (Case control)	Yes	Yes	Can't tell	No, Yes	High
**Gordon and Lubitz (2009)**	Yes, No	Yes (Case series)	No	No	Can't tell	Yes, No	High*
**Henderson (2014)**	Yes, No	No (Case series)	No	No	Can't tell	No, Yes	High*
**Denborough et al (2003)**	Yes, No	Yes (Case series)	No	Yes	Can't tell	Yes, Yes	High*
**Rowe (2019)**	No, No	No	Yes	No	No	Yes, Yes	Unclear

(b) Randomised controlled trials

Authors (year)	Did the trial address a clearly	Was the assignment of	Were patients, healthcare	Were the groups	Aside from the experimental	Were all of the patients who	Overall Rating using Cochrane

For peer review only - http://bmjopen.bmj.com/site/about/guidelines.xhtml

	focused issue? Was this the outcome of interest to this review?	patients to treatments randomised?	professionals and research staff blinded?	similar at the start of the trial?	investigation, were the groups treated equally?	entered the trial properly accounted for at its conclusion?	risk of bias scale RoB 2 (low/unclear/high)
**Nijhof et al (2012); Nijhof et al (2013)**	Yes, no	Yes	No	Yes	Yes	Can't tell	Low
**Rowe (1997)**	Yes, no	Yes	Yes	Yes	Yes	Yes	Low
**Wright et al (2005)**	Yes. no	Yes	No	Yes	Yes	Can't tell	Low
**Gordon et al (2010)**	Yes, no	Yes	No (pts), No (HCPs), Yes (assessors)	Yes	Yes	Yes	Low
**Crawley et al (2018)**	Yes, no	Yes	No	Yes	Yes	Yes	Low

For peer review only

For peer review only - http://bmjopen.bmj.com/site/about/guidelines.xhtml

For peer review only

For peer review only - http://bmjopen.bmj.com/site/about/guidelines.xhtml

PRISMA 2009 Checklist

Section/topic	#	Checklist item	Reported on page #
**TITLE**			
Title	1	Identify the report as a systematic review, meta-analysis, or both.	1
**ABSTRACT**			
Structured summary	2	Provide a structured summary including, as applicable: background; objectives; data sources; study eligibility criteria, participants, and interventions; study appraisal and synthesis methods; results; limitations; conclusions and implications of key findings; systematic review registration number.	2-3
**INTRODUCTION**			
Rationale	3	Describe the rationale for the review in the context of what is already known.	4-5
Objectives	4	Provide an explicit statement of questions being addressed with reference to participants, interventions, comparisons, outcomes, and study design (PICOS).	5
**METHODS**			
Protocol and registration	5	Indicate if a review protocol exists, if and where it can be accessed (e.g., Web address), and, if available, provide registration information including registration number.	3
Eligibility criteria	6	Specify study characteristics (e.g., PICOS, length of follow-up) and report characteristics (e.g., years considered, language, publication status) used as criteria for eligibility, giving rationale.	Table 1 page 6
Information sources	7	Describe all information sources (e.g., databases with dates of coverage, contact with study authors to identify additional studies) in the search and date last searched.	5-6
Search	8	Present full electronic search strategy for at least one database, including any limits used, such that it could be repeated.	Supplementary
Study selection	9	State the process for selecting studies (i.e., screening, eligibility, included in systematic review, and, if applicable, included in the meta-analysis).	7
Data collection process	10	Describe method of data extraction from reports (e.g., piloted forms, independently, in duplicate) and any processes for obtaining and confirming data from investigators.	7
Data items	11	List and define all variables for which data were sought (e.g., PICOS, funding sources) and any assumptions and simplifications made.	6
Risk of bias in individual studies	12	Describe methods used for assessing risk of bias of individual studies (including specification of whether this was done at the study or outcome level), and how this information is to be used in any data synthesis.	8-9
Summary measures	13	State the principal summary measures (e.g., risk ratio, difference in means).	7

For peer review only

PRISMA 2009 Checklist

Synthesis of results	14	Describe the methods of handling data and combining results of studies, if done, including measures of consistency (e.g., I^2) for each meta-analysis.	N/A

Section/topic	#	Checklist item	Reported on page #
Risk of bias across studies	15	Specify any assessment of risk of bias that may affect the cumulative evidence (e.g., publication bias, selective reporting within studies).	9
Additional analyses	16	Describe methods of additional analyses (e.g., sensitivity or subgroup analyses, meta-regression), if done, indicating which were pre-specified.	N/A
**RESULTS**			
Study selection	17	Give numbers of studies screened, assessed for eligibility, and included in the review, with reasons for exclusions at each stage, ideally with a flow diagram.	8
Study characteristics	18	For each study, present characteristics for which data were extracted (e.g., study size, PICOS, follow-up period) and provide the citations.	9-10and Table 2
Risk of bias within studies	19	Present data on risk of bias of each study and, if available, any outcome level assessment (see item 12).	8-9
Results of individual studies	20	For all outcomes considered (benefits or harms), present, for each study: (a) simple summary data for each intervention group (b) effect estimates and confidence intervals, ideally with a forest plot.	Table 4
Synthesis of results	21	Present results of each meta-analysis done, including confidence intervals and measures of consistency.	N/A
Risk of bias across studies	22	Present results of any assessment of risk of bias across studies (see Item 15).	8-9
Additional analysis	23	Give results of additional analyses, if done (e.g., sensitivity or subgroup analyses, meta-regression [see Item 16]).	N/A
**DISCUSSION**			
Summary of evidence	24	Summarize the main findings including the strength of evidence for each main outcome; consider their relevance to key groups (e.g., healthcare providers, users, and policy makers).	17-18
Limitations	25	Discuss limitations at study and outcome level (e.g., risk of bias), and at review-level (e.g., incomplete retrieval of identified research, reporting bias).	17
Conclusions	26	Provide a general interpretation of the results in the context of other evidence, and implications for future research.	17-18
**FUNDING**			
Funding	27	Describe sources of funding for the systematic review and other support (e.g., supply of data); role of funders for the systematic review.	20

For peer review only

PRISMA 2009 Checklist

From: Moher D, Liberati A, Tetzlaff J, Altman DG, The PRISMA Group (2009). Preferred Reporting Items for Systematic Reviews and Meta-Analyses: The PRISMA Statement. PLoS Med 6(7): e1000097. doi:10.1371/journal.pmed1000097

For more information, visit: **www.prisma-statement.org**.

For peer review only

For peer review only - http://bmjopen.bmj.com/site/about/guidelines.xhtml
